# Surface‐Sensitive Fractioning of Flowing Colloidal Suspensions Sedimented at a Photochemically Active Wall

**DOI:** 10.1002/smll.202500012

**Published:** 2025-05-12

**Authors:** Daniela Vasquez‐Muñoz, Mihail Nicola Popescu, Anjali Sharma, Fabian Rohne, Isabel Meier, Phillip Ortner, Sarah Loebner, James Robert Benson, Nino Lomadze, Stephan Eickelmann, Svetlana Santer, Marek Bekir

**Affiliations:** ^1^ Institute for Physics and Astronomy Karl‐Liebknecht‐Str. 24/25 14476 Potsdam Germany; ^2^ Física Teórica Universidad de Sevilla Apdo. 1065 Sevilla 41080 Spain; ^3^ International Centre of Biodynamics 1B Intrarea Portocalelor Bucharest 060101 Romania

**Keywords:** azobenzene containing surfactant, chemical activity, fractioning, light‐driven diffusio‐osmosis, microfluidics, microparticle hovering, phoresis

## Abstract

A colloidal particle exposed to nonequilibrium inhomogeneities in the chemical‐composition of the solution responds by phoresis with a magnitude dictated by its surface properties (adsorption of solutes relative to the solvent) and the strength of the inhomogeneity gradient. Fractioning by surface‐properties for a suspension of sedimented colloidal particles flowing within a microfluidic device can then be achieved by exposing it to chemical‐composition gradients normal to the wall. One such method is developed by employing a wall imbibed with a photo‐sensitive surfactant; under suitable illumination, it undergoes reversible isomerization and turns the wall into a source of chemical inhomogeneities by isomers‐exchange with the solution. Proof‐of‐concept experiments, complemented by theoretical modeling, demonstrate the feasibility of the proposed method in several examples of particles mixtures. The results highlight its potential in terms of high performance, simple technical requirements, and suitability for dealing with equally‐sized—but surface different—particles.

## Graphical abstract

A straightforward technique for fractionating sedimented, same‐sized colloidal particles based on small differences in their surface properties is described. This microfluidic technology method exploits the velocity differentials within a shear‐flow via inducing a phoretic lift above the bottom porous polymer interface that is capable of light‐induced release of a stored photosensitive surfactant.

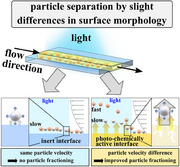

## 1 Introduction

Separation of microparticles is an important technological problem, not only because of classical applications (e.g., removal of pollutants in environmental engineering, separation of valuables from tailings in the mining industry) but also because the colloids are turning into building blocks for many modern applications related to, e.g., biochemical analysis and clinical medicine,^[^ ^1^, ^2^, ^3^ ^]^ or assessment of water quality.^[^ ^4^, ^5^, ^6^ ^]^ Additional, but not exhaustive, examples are their use: in electronic devices such as TV screens or computer monitors;^[^ ^7^, ^8^ ^]^ in medicine, as drug‐carriers ^[^ ^9^, ^10^, ^11^, ^12^ ^]^ or labeling tools;^[^ ^13^, ^14^, ^15^ ^]^ as carrier particles in heterogenous catalysis;^[^ ^16^, ^17^, ^18^ ^]^ as precursor for fabrication of concrete,^[^ ^19^ ^]^ etc.

Currently, there is plethora of separation/sorting methods, at the small (laboratory).^[^ ^20^, ^21^, ^22^, ^23^, ^24^ ^]^ and the large (industry) scale,^[^ ^25^, ^26^, ^27^ ^]^ but with many of them targeting the particle size, or the core (bulk) magnetic,^[^ ^28^, ^29^, ^30^, ^31^ ^]^ or dielectric properties,^[^ ^32^, ^33^, ^34^, ^35^, ^36^, ^37^ ^]^ as the class parameter. On the other hand, many of the advanced technologies based on colloids require particles with very specific, well defined surface properties. This leads to a need for sorting of similarly‐sized particles, having similar core material, by theirs surface properties in order to achieve, e.g., a purification of unwanted side reactions, or a narrowing of the charge distribution of polymeric particles.^[^ ^38^ ^]^ Yet, with the notable exception of the widely employed electrophoretic‐type separation by surface‐charge,^[^ ^32^, ^39^, ^40^ ^]^ such surface‐properties sensitive separation methods, with very good selectivity and versatility, are less developed for particles in the sub millimeter scale.^[^ ^41^, ^42^, ^43^ ^]^

By targeting the interfacial properties of particles, like in the studies in, e.g., Refs.,^[^ ^44^, ^45^, ^46^ ^]^ it should be possible to achieve selective sorting not only by interfacial charge (charged vs neutral, anionic vs cationic), but also by, e.g., differences in surface morphology (rough and/or porous vs compact interfaces) or in surface tension (adsorption potential) of the particle‐suspension interface. Along this line of research, we have recently shown that fractionation of microparticles (by retention time along a microfluidic channel flow), as function of particles surface porosity, or material‐nature of the particle, or by the surface functionalization, can be achieved by turning them chemically active.^[^ ^47^, ^48^ ^]^ At the core of the method is a photosensitive surfactant, whose light‐induced reversible photoisomerization triggers a chemical activity of the particles in the form of a steady exchange of isomers between its surface and the surrounding suspension. This chemical activity triggers phoretic/osmotic flows for of a variety of hard^[^ ^49^, ^50^ ^]^ and soft^[^ ^51^, ^52^ ^]^ microparticles, a mechanism reported as *local*‐light driven diffusioosmosis (*l*‐LDDO).^[^ ^49^, ^53^, ^54^, ^55^ ^]^ For spherical particles, the *l*‐LDDO can be exploited by distorting the spherical symmetry of the inhomogeneities induced by the chemical activity of the spherical particle through the proximity of a wall, as done for the fractionation method described in Ref.[47], in order to achieve a hovering of micro sized (1–30 µm in diameter) particles at various distances from the wall. When additionally exposed to a pressure driven fluid flow, the variations in the flow velocity with the distance from the wall ensure that differently hovered particles acquire a different drift velocity.^[^ ^47^, ^56^, ^57^ ^]^ Since particles with different interfacial properties, which have dissimilar chemical activities (owing to absorbing different amounts of surfactant, but also to different rates of the isomerization reaction) and different phoretic mobilities (response to gradients in the chemical composition of the solution), will hover at different heights, a differential in their drift velocity emerges, and thus fractionation along the microfluidic channel is achieved.^[^ ^47^ ^]^

Although the method is very promising, it relies on the capacity of the particles to adsorb enough surfactant (in order to exhibit a sufficiently strong chemical activity), which in general is not the case; moreover, the dependence of the hovering on both the local activity and the surface properties complicates a separation “solely by surface properties”. Motivated by these aspects, we show here that such limitations can be elegantly bypassed by shifting the chemical activity from the particle to the wall of the channel. This removes the requirement that the particles adsorb sufficient surfactant, but we continue to exploit their surface‐properties sensitive, robust phoretic response in order to achieve differential hovering and thus fractionate the flowing suspension. (We note that chemically active walls that repel colloids (leading to the creation of an “exclusion zone”) are already known in literature,^[^ ^58^, ^59^, ^60^ ^]^ one of the most famous examples being Nafion (a perfluorinated polymer composed of deprotonable functional moieties) and its derivates. This works via a “proton‐exchange” mechanism and can make exclusions zone up to hundreds of micrometers in thickness. However, this activity decays over time, until the Nafion‐fluid interface is fully deprotonated.^[^ ^59^ ^]^

In this paper we report on the development of such a surface‐properties sensitive separation method based on this active‐wall strategy. Our active wall consists of a thick, porous polymer layer (poly (4‐vynilpyridine‐co‐butyl methacrylate), commercial with ≈90% 4‐vinylpyridine) coated on a glass support. The polymer layer is capable of absorbing huge amounts of the azobenzene‐containing photosensitive surfactant in *trans* isomeric state, and, when illuminated,^[^ ^61^, ^62^ ^]^ it expels into the solution the *cis*‐converted (which are more hydrophilic) isomers. This establishes the necessary chemical activity for the hovering of sedimented microparticles. Any intrinsic activity of the particles, which may have adsorbed surfactant themselves, contributes to the net hovering through a superposition of effects from both the wall and the particle activity. Consequently, we introduce a dual phoretic activity originating from both the particle and the active wall. Both activities are induced by a single light source and influence the overall hovering height. This leads to a highly sensitive differential in drift velocity for particles that are chemically similar but differ slightly in their surface properties. As a result, fractionation based on velocity differences can be achieved. Through proof‐of‐concept experiments, supported by theoretical considerations, we demonstrate the potential of this method for separating a wide range of binary mixtures of microparticles that are of similar size but exhibit slight variations in interfacial properties.

## 2 Results and Discussion

The chemically active wall is made by using a glassy, transparent, rigid, non‐water‐soluble polymer, poly (4‐vinylpyridine‐co‐butyl methacrylate), in the following abbreviated as PVPBMA, which can be loaded with large amounts of the azobenzene‐containing photosensitive surfactant. Photosensitive surfactants can be released on demand via light illumination, for instance with UV (365 nm) light (and other wavelengths, too), and the release maintains a steady rate over several hours. The details regarding the making of the spin‐coated PVPBMA wall, as well as the thorough characterization of the PVPMA film, of its surfactant absorption, and of the kinetics and the temporal extent of the surfactant release upon illumination are provided in the Section S1 (Supporting Information).

Furthermore, the wettability of PVPBMA layer can be altered upon loading/releasing photosensitive surfactant molecules into/from the polymer matrix interior. Basically, the PVPBMA changes from strong hydrophobic into strong hydrophilic, when *trans*‐isomer surfactant is adsorbed in the polymer matrix or become strong hydrophobic again when the *cis* surfactant is released during UV illumination. Thus, the PVPBMA (originally hydrophobic) become on *cis*‐release hydrophobic again. The interface properties have been measured from contact angle measurement, which are displayed in detail in the Supporting Information, Section S.1.2.3. When exposed to light of a suitable wavelength, for example *λ* = 365, 455, and 490 nm as used in the following, the PVPBMA interface releases *cis*‐isomers into the surrounding solution (while in‐taking *trans*‐isomers from the solution). The isomer release causes a chemically active surface with a steady isomer‐exchange rate (after a few seconds transient following the turning‐on of the light exposure).

Accordingly, we expect that, when the system is illuminated in one of the wavelengths above, the colloidal particles sedimented at such a (azobenzene surfactant loaded) PVPBMA‐coated glass wall will exhibit a phoretically‐induced vertical displacement from their sedimentation equilibrium to a new (“hovering”) position, where the sedimentation velocity is balanced by the elevation‐dependent phoretic velocity (see the details in the Theoretical Modeling Section). This expectation is verified in two ways.

**Figure 1:**
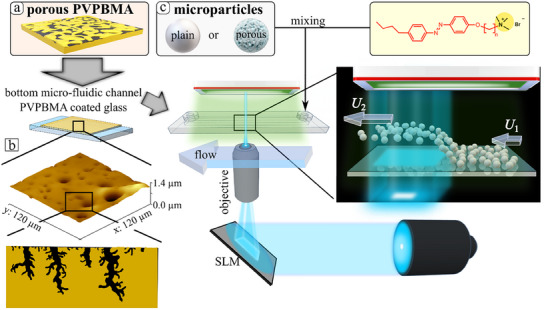
Illustration (not at scale) of the experimental setup. a) Schematic representation of a porous PVPBMA interface which absorbs the azobenzene containing surfactant. b) AFM height image of PVPBMA in 3D view together with a possible scheme of the pore structure (bottom). c) Dispersion of compact and porous microparticles and solution of azobenzene containing surfactant are injected into the rectangular microfluidic chamber. Yellow rectangle displays the chemical structure of azobenzene‐containing trimethyl‐ammonium bromide surfactant (full name 6‐[4‐(4‐Hexylphenylazo)‐phenoxyl‐butyl‐trimethylammoniumbromide, C_4_‐Azo‐OC_6_ TMAB). Once particles fully sediment at the bottom layer (PVPBMA or glass as reference) a pressure‐driven fluid flow is switched on. The motion and trajectory of passively transported particles is recorded via video microscopy. The “plain” label indicates compact (nonporous) particles. In the region under illumination (the blue shades), the wall is active due to the photoisomerization of the surfactant and the consequent exchange *cis* out–*trans* in with the solution; once in this region, the particles experience the phoretic hovering and, thus, a change in their drift velocity by the ambient flow.

The first one is by flowing a mixture of compact and porous, respectively, silica particles of equal size (diameter *D* ≈ 3‐4 µm) dispersed in the aqueous solution of the photosensitive surfactant through a microfluidic channel of rectangular shape (height = 0.54 mm, width = 3.8 mm) with the bottom wall either of glass or surfactant‐loaded chemically active PVPBMA coated glass (**Figure** **1**a). As shown in Figure 1a, the experimental setup is such that only a rectangular area of ≈230 µm × 270 µm is illuminated by collimating the light beam, and the velocity of particles moving within the objective view‐area (Figure 1b,c) can be determined by video tracking. Since under a pressure driven shear flow the sedimented particles experience a translational velocity that is approximately proportional to the shear rate *S* (here *S* ≈ 15 s^−1^, fixed by the volumetric flow rate of 150 µL min^−1^ through the channel; this was kept the same in all the microfluidic flow experiments discussed in this paper) and the height *h* of the particle's center above the wall (at the no‐slip wall the flow velocity being zero) the eventual changes in the observed drift velocity of a particle upon turning on the light can be directly interpreted as changes in the hovering elevation.

**Figure 2:**
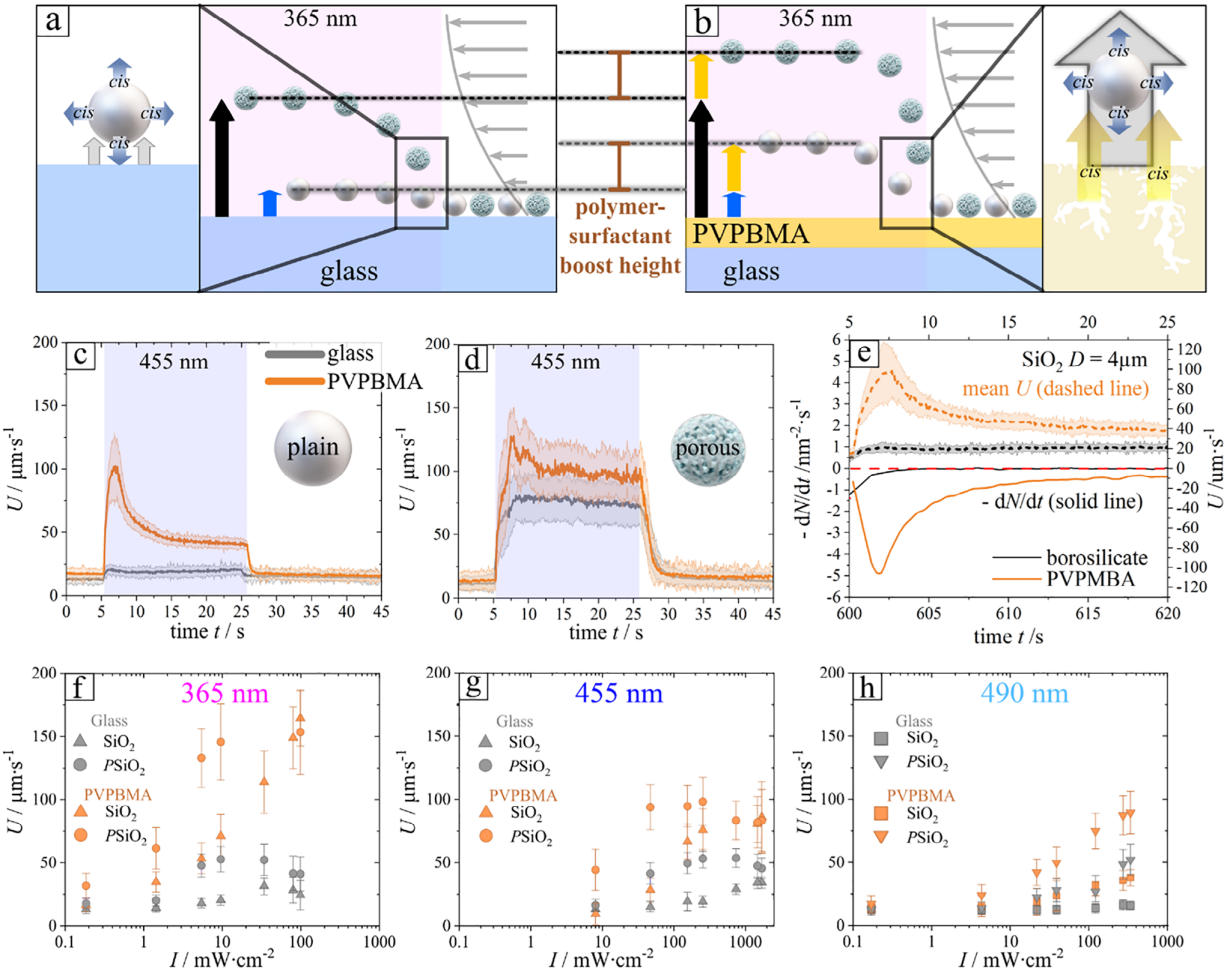
a,b) Scheme of the velocity‐boosting effect on compact and porous SiO_2_ microparticles sedimented at glass and polymer interface, respectively. Blue and black arrows represent the lift of compact and porous silica microparticles by LDDO mechanism, respectively, while yellow arrow represents the additional lift boosting from polymer surface. c,d) Mean drift velocity (line) and corresponding standard deviation (filled transparent area) as a function of time for compact (c) and porous (d) SiO_2_ microparticles. e) Release rate of surfactant molecules (orange solid line) and mean velocity of compact SiO_2_ particles on polymer (orange dashed line) and glass (black solid line = release rate, black dashed line = mean velocity) surface. A clear correlation between the release of surfactant by the wall and the increase in the drift velocity is noticeable. The blue rectangles indicate the illuminated (*λ* = 455 nm) region, while the time stamps refer to the moment when the recording started (5 s before the light is turned on). f–h) Mean drift velocity of particles under illumination (after 15 s of illumination, *t* = 20–25 s) displayed as a function of the light intensity at *λ* = 365 nm (f); *λ* = 455 nm (g) and *λ* = 490 nm (h) for compact (triangles) and porous (circles) SiO_2_ microparticles above a glass (gray symbols) or a PVPBMA polymer (orange symbols) wall, respectively. The means are computed over an average sample size of *n* particles per frame of d) glass wall: *n* = 209 ± 11, PVPBMA wall: *n* = 189 ± 11; e) glass wall: *n* = 158 ± 18, PVPBMA wall: *n* = 123 ± 23. or the data in (f–h) the corresponding average sample size per frame is summarized in Table S3 (Supporting Information). Measurements were done by illuminating only a specific region.

The corresponding drift velocities under the pressure driven shear flow for compact silica particles with and without illumination with blue light (455 nm) are shown in **Figure** **2**c (the light is on during the time interval *t* = 5–25s) for a PVPBMA wall (see also Video S1, Supporting Information) and a glass wall (see also Video S2, Supporting Information). Clearly, there are very weak changes in the velocity of the compact particles when the glass wall is used, and significant ones in the presence of the PVPBMA wall. In the former case, these are the result of the fact that the particles are themselves weakly active, as noted in the Section 1, a case analyzed in detail in Ref. [47]. In the latter case, one observes a transient spike in velocity, which increases from a 10–15 µm s^−1^ in the absence of illumination to ≈100 µm s^−1^ during the first 3 s of illumination, followed by a decrease toward a steady state mean velocity of ≈50 µm s^−1^. These trends clearly correlate with the similar features (spike and decrease to a steady state, Figure 2d) observed in the study of the release rate of *cis* isomers (see Figure 2e; Section S1.2, Supporting Information), which supports the view that the changes in the velocity are due to a phoretic response by the particle to the chemical activity of the PVPBMA wall.

The theoretical modeling of chemical activity of planar interface is supported from experimental data and can be better visualized from data displayed in Figure 2e. For that the releasing rate of molecules d*N*/dt is plotted (solid line, Figure 2e) in the initial 20 s of illumination and compared from mean velocity (dotted line) for PVPBMA and glass interface. Velocity gain and releasing rate show the temporal maximum at ≈2.5 s after UV illumination and adjust to a smaller value with increasing time. This is because the first initial rapid burst of *cis*‐isomer from the outer interface temporarily hovers the particles stronger, where a subsequent slower release rate from pores and polymer matrix maintain an “almost steady state” of effective hovering of compact (∼nonporous) particles in comparison to glass interface. This is maintained as long as the isomer is adsorbed in the polymer during illumination. However, the total release of all isomers requires several hours (Figure S2g, Supporting Information). Thus, for the short time frame of illumination (≈20 s), the activity is steady and significant, thus the boosted height has a pronounced impact on the motion velocity.

We performed further similar experiments, at the same light intensity, with porous silica colloids, (Figure 2d; Video S3 and Video S4, Supporting Information), which are by themselves chemically active when illuminated (see Ref. [47]). The results differ from those in the case of compact particles only in that also in the glass wall case a pronounced velocity gain, from ≈12.5 µm to ≈75 µm s^−1^ is observed, in agreement with the previous report of a significant “self‐activity” of the particles (Video S4, Supporting Information).^[^ ^47^ ^]^ For the PVPBMA wall one observes a transient spike in the velocity to ≈125 µm s^−1^ followed by a decrease toward a steady state mean velocity of ≈100 µm s^−1^. These features perfectly agree with the intuitive expectation of a superposition of phoretic responses (to the self‐activity and to the wall‐activity) leading to a larger hoovering elevation, and thus larger drift velocity in the pressure‐driven microfluidic flow, then in the cases in which either the particle activity (compact vs porous particle) or the wall activity (glass vs PVPBMA) are missing.

Furthermore, a detailed statistical analysis of the data (Figure 2c,d) is provided in the Section S3.3 (Supporting Information). The drift velocity distribution for compact particles (Figure S14, Supporting Information) shows a narrow Gaussian profile in the steady state (time range 15–25 s) for both the active (PVPBMA) and inactive (glass) planar substrates. In contrast, porous particles exhibit a broader, right‐skewed distribution, presumably due to the significant variation in the porosity (∼effective surface area) of each individual microparticle (Figure S15). This variation results in a distribution of levitation heights, with each particle having its own phoretic strength, thereby producing a broader distribution of drift velocities. Interestingly, the drift velocity distribution for both the active and inactive interfaces in the steady state (time range 10–25 s) remains within the same range and exhibits a similar profile. However, the mean, minimum, and maximum values for the active interface are shifted toward higher velocities. We conclude that the active interface does not significantly alter the distribution, but instead shifts the mean drift velocity to higher values. This shift is attributed to the uniform release of isomers from the active interface, with the variation in levitation stemming solely from the distribution of phoretic strengths among the particles. These findings are in reasonable agreement with the experimental data shown in Figures S14 and S15 (Supporting Information).

Additionally, we observe a spatial ordering during illumination visualized as a time series of Voronoi diagrams^[^ ^54^, ^63^ ^]^ in Video S5 (Supporting Information) for compact (SiO_2_) and in Video S6 (Supporting Information) for porous (PSiO_2_) particles and also visualized as a snapshot series displayed in Figures S16 and S17 (Supporting Information). The Voronoi diagrams exhibit the pronounced ordering of particles from active interface only then, if the particles are inactive. The active particles already show a strong natural lateral displacement strength.^[^ ^54^ ^]^ Thus one cannot clarify if the ordering is only source from the bottom interface or from the particles itself. Data in Figure S17 (Supporting Information) clearly show that for glass and PVPBMA under illumination porous particles strongly separate from each other.

In order to verify the robustness of the findings with respect to variations in the wavelength and the intensity of the illumination, we have studied the changes in the velocities for the same system described above at three wavelengths (*λ* = 365, 455, 490 nm) and for four decades of variations in the light intensity. The results summarized in the Figure 2f–h show that in all cases an increase in the illumination intensity yields to an increase in the velocity gain, and that always the drift velocities under illumination are faster when using the PVPBMA wall than when using the glass wall. (The changes in velocity toward the large intensity end show a saturation tendency, which is expected since the rate of chemical activity of the polymer or of the particles must be bounded, if not by other factors, then at least because there is maximum capacity of loading *trans*‐isomer for the photoconversion to proceed). As discussed in Supporting Information, this correlates with the photo‐isomerization kinetics, which, within the range of light intensities studied, is proportional to the intensity. The largest changes in the drift velocity, thus the largest elevations, occur in the case of illumination with UV light (*λ* = 365), with a gradual decrease in the magnitude of the changes as the wavelength is changed toward the larger values (compare panels (f) to (g) of Figure 2). This result also correlates well with the values of the *trans*‐*cis* isomerization rate constant (see the details in Section S1.2.1, Supporting Information). Therefore, the changes in the drift velocities, which reflect changes in the elevation above the wall, indicate larger elevations for increased rate of chemical activity of the particle and/or the wall, a trend consistent with a scenario of phoretic response of the particle to the chemical activity of the wall.

**Figure 3:**
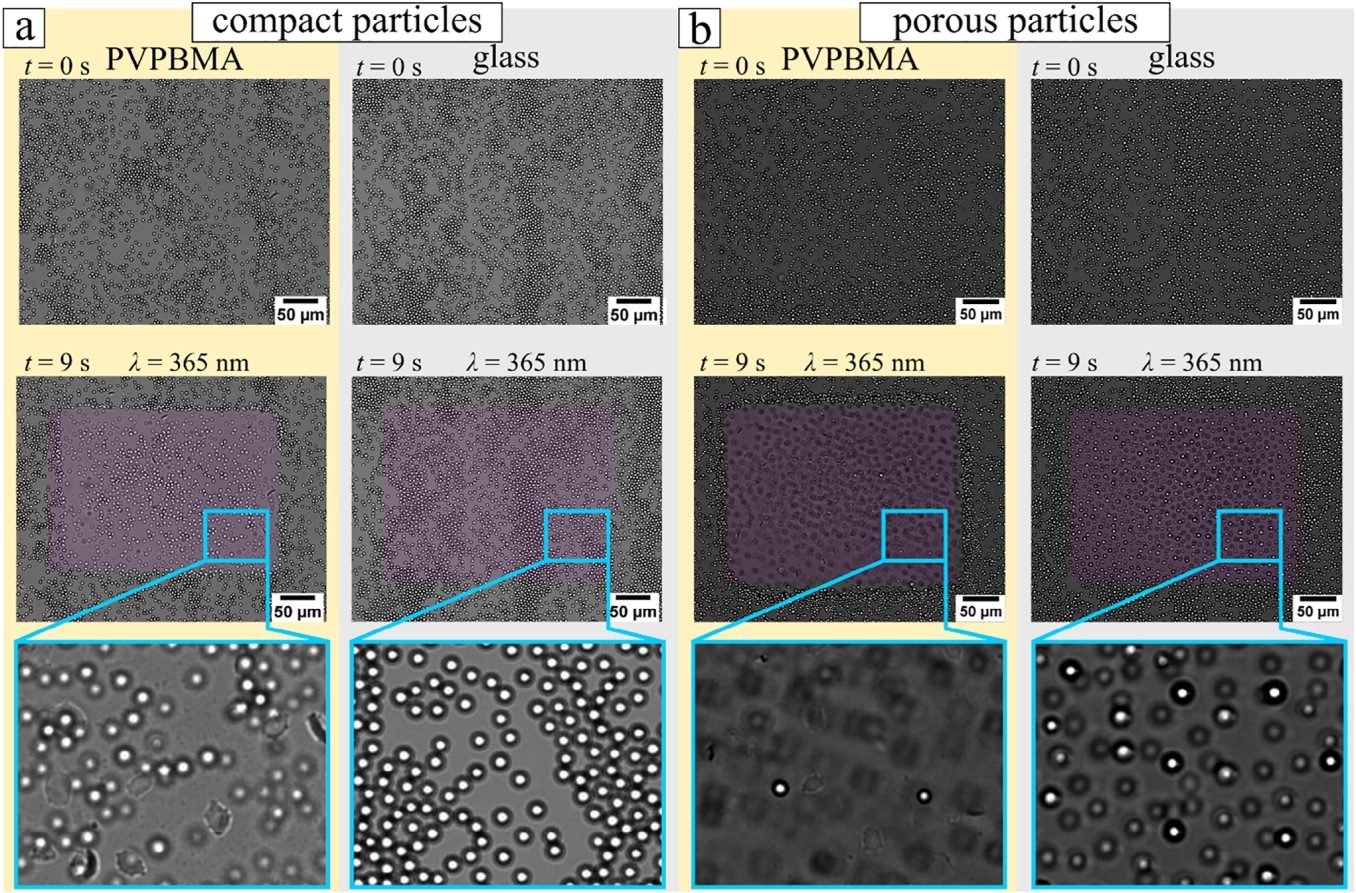
Snapshots (from Videos S7 and S8, Supporting Information) of optical view of the compact (of size *D* = 4 µm) and porous particles (of size *D* = 3 µm) before illumination (*t* = 0 s) and at time *t* = 9 s after the modulated light spot with moderate intensity (*λ* = 365 nm, *I* = 10 mW cm^−2^) was turned on. The focal plane is maintained at the *t* = 0 position, and thus the elevation of the particles in the rectangular‐shaped illuminated area results in out‐of‐focus imaging. The external flow is switched off. Measurements were done by illuminating only a specific region.

The second way of assessing the validity of the hypothesized change in the elevation of the particles above the chemically active wall upon illumination is by direct video microscopy observation of the defocusing of the images of particles when the system is visualized in the absence of external flow while illuminating only a part of the surface. The results when using compact and porous particles and illumination with *λ* =  365 nm at intensity *I* =  10 mW∙cm^−2^ are shown in **Figure** **3** (see also Video S7 (Supporting Information), for compact particles of size *D* = 4 µm, Video S8 (Supporting Information), for particles of size *D* = 3 µm, and Video S9 (Supporting Information), for particles of size *D* = 5 µm) when above a glass and a PVPBMA wall, respectively. It is clear that a stronger diffraction pattern smearing emerges when the PVPBMA wall is used. In conjunction with the fact that there is no external flow, these results then unequivocally attribute the change in elevation to the chemical activity of the particle (from the elevation above the glass wall) and to the wall activity, when comparing the elevations under light in the cases when either the glass or the PVPBMA wall, respectively, are used. Furthermore, the lack of elevation of the compact particles above the glass clearly demonstrates that they are only very weakly active, and thus their elevation above the PVPBMA wall can be unequivocally attributed to their response to the chemical activity of the wall.

The two components (particle and wall activities) contributing to the elevation and hovering of the particles in the presence of illumination can be rationalized and qualitatively described by using a simple model of chemical activity and phoretic response, as previously done in Ref. [47]. Since the self‐phoresis induced by the chemical activity of the particles has been analyzed in detail in Ref. [47, 50], here we focus on the response to the activity of the wall and leave the details of the calculations of the combined response for the Theoretical Methods Section and Supporting Information. In brief: at the steady state (i.e., after a short transient burst, see Figure 2e) the chemical activity of the PVPBMA wall is modeled as the release, with a constant rate *Q* that is uniform over the illuminated region, of *cis*‐isomers that are diffusing into the surrounding solution. The resulting chemical composition of the solution, denoted by *c*(s), with s the position vector in the solution (the origin is taken at the center of the illuminated area), is inhomogeneous due to the fact that the release happens only at the illuminated region at the wall, but not in the bulk. This gives rise to phoretic motion of a particle located at *h* above the wall with a phoretic velocity **V**_ph_ that is proportional to the local gradient of *c*(s); the proportionality constant is negative (“repulsion”), if the particle prefers a depletion of *cis*‐monomers and an excess of *trans*‐monomers (as expected for a hydrophobic surface, like PS), and positive (“attraction”) in the opposite (preference for excess of *cis*‐monomers) case. By assuming that the diffusion of the surfactant isomers is much faster than the convection by the hydrodynamic flow that the phoretic motion of the particle induces, and considering (for reasons of technical simplicity of calculation) a disk‐like shape of the illuminated area, *c*(**s**) and **V**_ph_ can be straightforwardly calculated (see the details in Theoretical Methods Section and in Supporting Information, Section S4).

**Figure 4:**
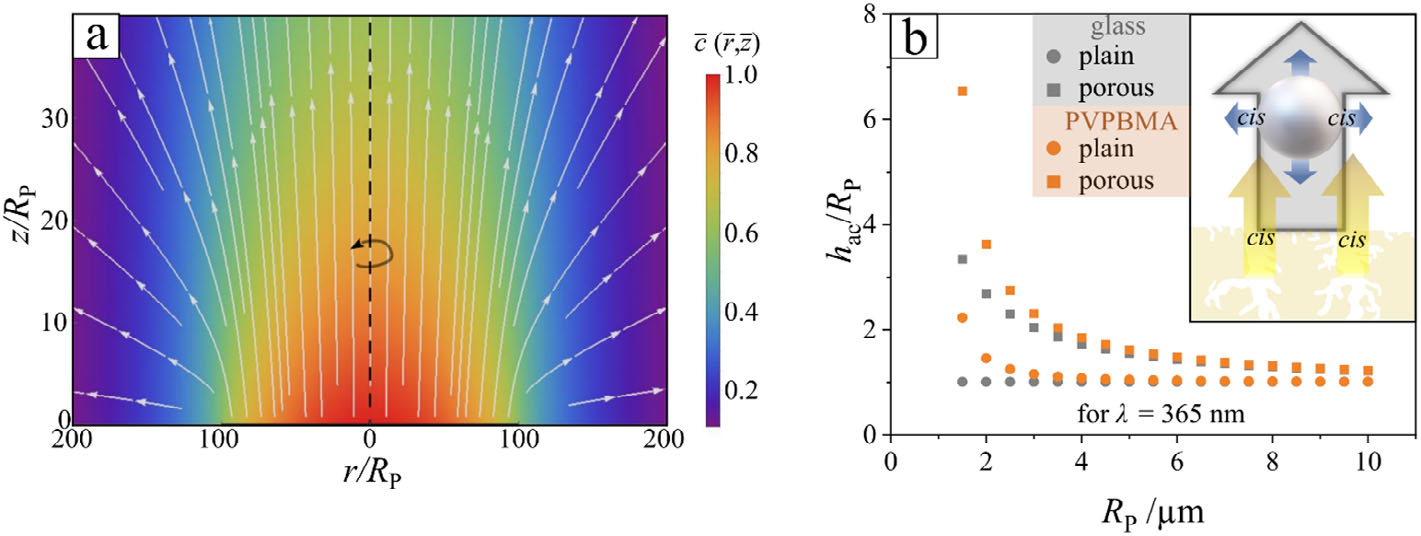
a) Theoretically calculated density of *cis*‐monomers (color coded) and streamlines of its gradient, i.e., the phoretic velocity response of a particle. The results correspond to a patch of radius 100 *R*_p_, which is similar to the size of the illuminated area in the experimental setup, and to repulsive interactions between the particle and the *cis*‐isomers. Note that near the wall the streamlines are basically vertical over the whole surface of the patch, i.e., the phoretic velocity is to a very good approximation along the direction of the sedimentation velocity. b) Theoretically calculated (see Theoretical Methods Section and Section S4, Supporting Information) elevation heights (under illumination) above a glass (gray symbols) or a PVPBMA (orange symbols) for plain (circles) or porous (squares) silica particles.

A typical result is shown in **Figure** **4**a for the case of “repulsion”, which is consistent with the similar assumption made in Ref. [47], in the case of the self‐phoretic response of particles, where the color codes the *cis*‐monomer density *c*(s) (in units of a characteristic density) while the white arrow show the streamlines of the gradient in *c*(s), i.e., the resulting phoretic velocity of the particle as a function of position. Note that in Figure 4a all the lengths are given in units of the particle radius *R*p. There are few features worth emphasizing. First is that for most of the region illuminated the induced phoretic response is exactly normal to the wall, as assumed and in agreement with the experimental observation discussed in Figure 3; this is due to the fact that the lateral extent of the illuminated region is much larger than the particle size, and thus the inhomogeneities in the lateral direction manifest only over large spatial length scales, and in particular near the edges of the illumination pattern. This also provides justification for neglecting any influence of osmotic flows induced by the activity of the wall on the motion of the particle (if at all, such flows would influence the motion at the edge of the illuminated region). Second, by the same token of large scales compared to the size of the particle, the vertical spatial gradients are actually relatively weak, although the rate of *cis* release might be large, and thus the response to an active wall is not necessarily much different in magnitude from the one due to the self‐activity (in the case of a porous particle), in agreement with the experimental observations discussed in Figure 2.

The theoretical predictions emerging from the modelling that accounts for both responses (to self‐activity and to the wall activity, see Theoretical Methods Section and Supporting Information) suggest that for the heavy silica particles the gain in elevation (hovering height) due to the wall activity of PVPBMA is very significant for small particles but quickly decays with increasing the size of the particle, as illustrated in Figure 7.

Returning to a point made earlier in the paper, while flow fractioning of equally sized particles slight differing in their surface morphology is feasible by using the strategy of phoretic response to chemical activity owing to the relatively significant self‐activity of porous particles,^[^ ^47^ ^]^ the results above clearly suggest that by using an active wall the same strategy can be extended toward an interfacial sensitive fractioning of microparticles with only weakly active particles (which is the rather generic case, since most compact particles are possessing very weak chemical self‐activities).

**Figure 5:**
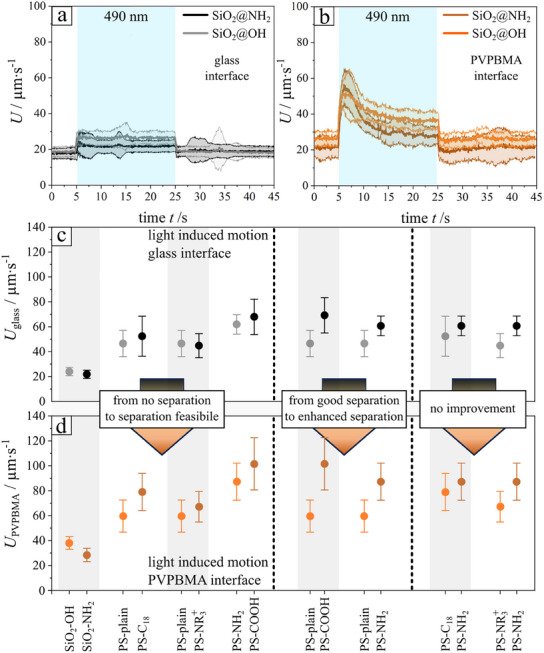
a,b) Mean velocity (thick lines) and the corresponding standard deviation (transparent area) as a function of time for compact SiO_2_ microparticles above a glass wall (a) and a PVPBMA active wall (b). The particles have different surface functionalization, being either unmodified, thus with a hydroxyl‐ (─OH) group, or modified with an amino‐group (─NH_2_). The blue area marks the time interval during which the light (*λ* = 490 nm) is on. Only the data in the last 5 s of illumination *(t* = 20–25 s) is used in the calculation of the average mean velocity and standard deviation shown in (c) and (d). c,d) Summary of the average velocity for example pairs of “weakly chemically active “ microparticles with different surface modification in the case of motion above a (c) glass and (d) PVPBMA wall, respectively. SiO_2_ = silica, PS = polystyrene, compact = plain = no surface modification (─OH group), Aminogroup = ─NH_2_, ternary functionalized amines = ─NR_3_^+^, Octyldecylgroup = C18, Carboxylgroup = ─COOH. The corresponding raw data (time resolved average velocity) is shown in the Figures S18–S20 (Supporting Information). Measurements were done by illuminating the region globally. Particle diameter is 5 µm.

We demonstrate experimentally that this indeed is feasible for a variety of binary mixtures of weakly active particles. For that we use the same microfluidic flow experimental setup as depicted in Figure 1, but we change the illumination profile from locally into a globally collimated light with a wavelength of 490 nm. This means that the entire bottom interface is illuminated. Moreover, guided by the theoretical insights, we restrict here to particles in the size range of 2–5 µm diameter, for which the active hovering effects are most pronounced (Figure 4b). In **Figure** **5**a,b (and the corresponding video Video S10, Supporting Information) we show the average drift velocity (bold line), together with standard deviation (transparent area), for compact silica particles differing only in their surface modification with hydroxyl‐ (─OH) or amino‐ (─NH_2_) groups, respectively. When the glass wall is used (Figure 5a), both types of particles show basically the same drift velocity, in the absence of light and when illuminated. However, for the same experimental setup but with the PVPBMA wall (Figure 5b), when illuminated the particles relax, after that previously discussed transient (≈10 s) spike related to the initial fast release of *cis*‐surfactant) to significantly different steady state average velocities, and thus fractionation along the flow direction is feasible.

**Figure 6:**
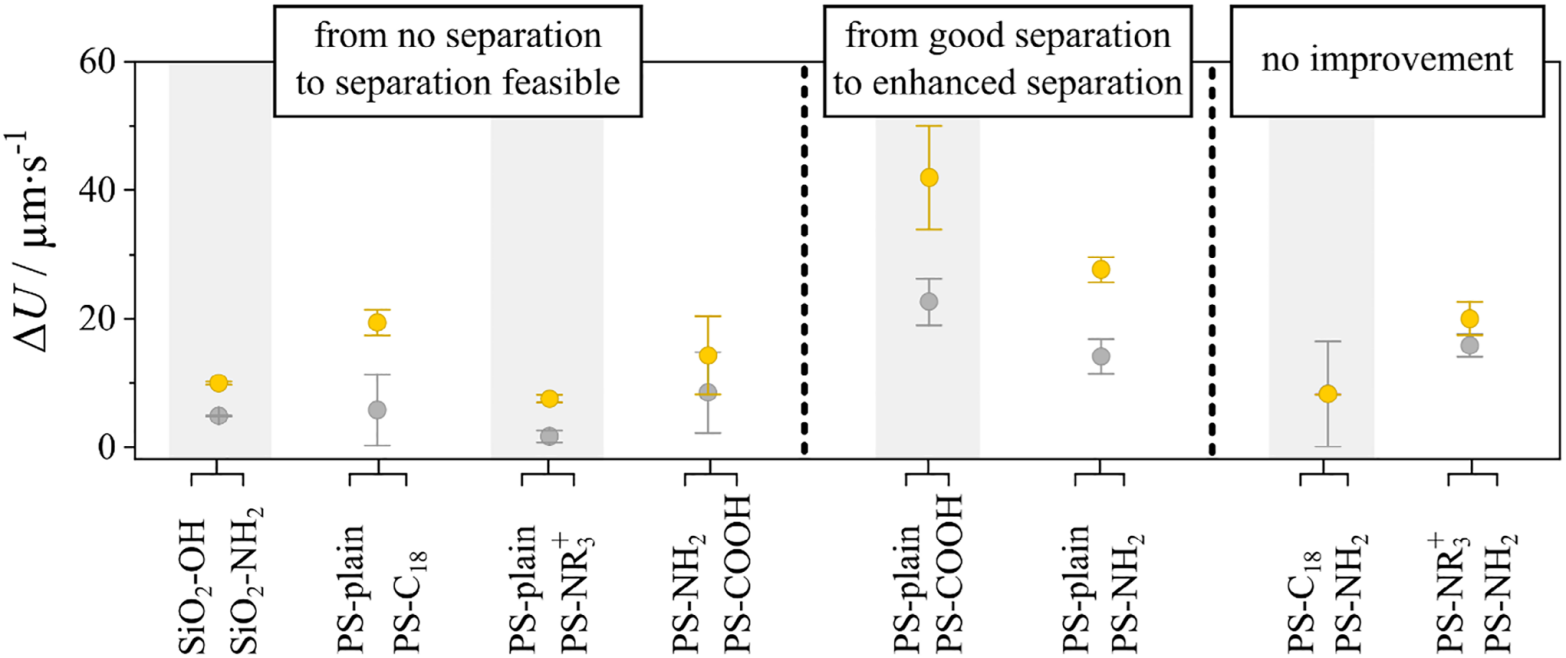
Summary of velocity difference from the data in Figure 5c,d as a function of the example pairs of “weakly chemically active “ microparticles with different surface SiO_2_ = silica, PS = polystyrene, plain = no surface modification (─OH group), Aminogroup = ─NH_2_, ternary functionalized amines = ─NR_3_^+^, Octyldecylgroup = C_18_, Carboxylgroup = ─COOH. The gray symbols are results in the case of an inactive glass wall, and the yellow symbols correspond to the results in the case of a chemically active PVPBMA wall. Measurements were done by illuminating the region globally. Particle diameter is 5 µm.

The results of similar experiments performed with other types of pairs of particles are summarized in Figure 5c,d, with the corresponding velocity difference (which is the crucial parameter for the chromatographic fractionation) achieved by the illumination‐induced activity of the wall displayed in Figure 5. For example, polystyrene particles with different surface modifications show (panels (c‐d), see also Video S11, Supporting Information) the same trend as for the silica particles (panels (a‐b), see also Video S10, Supporting Information) and corresponding Figure 5a,b), a conclusion that extends also to silica particles with a broad variety of surface coatings (Figures 5c,d and **6**).

These observations converge to the conclusion that separation of weakly active particles is now possible via the “phoretic elevation” response to the light induced activity of the wall. Rephrasing the summary above, one can also say that for particles pairs with varying surface coatings we distinguish 3 types of outcomes concerning the velocity differential Δ*U* = │*U*_1_−*U*_2_│ when from an inactive wall (glass) to a chemically active PVPBMA interface, as shown in Figure 5, 6: 1) from no separation to separation, 2) from good separation to enhanced separation, 3) no gain.

## 3 Properties of the PVPBMA and Limits of Proposed Separation Method

In the previous discussion section, we focused on the fundamental aspect of how the phoretic activity of the bottom planar substrate and that of the yields to levitation can improve the velocity difference on a particle slightly varying in interfacial morphology. In practice, separation is more complex and several points should be considered, likewise the separation time needed, particle size limit, throughput quantities, and other limitations. These are now classified as follows.

### 3.1 Separation Time Limit

By comparing the required separation time *t*_S_ with the total possible release time of the phoretically active bottom substrate (PVPBMA), it is essential to assess whether the activity at the PVPBMA interface remains effective or if the substrate depletes its fuel before the complete separation of the binary particle mixture is achieved. To verify this, we analyze the desorption time of the surfactant at the PVPBMA interface, as shown in Figure S2g (Supporting Information). The data indicates a total release duration of ≈3 h; however, a substantial release occurs predominantly within the initial phase of illumination. Specifically, we consider the period during which the surfactant concentration in Figure S2g (Supporting Information) decreases by a factor of e, corresponding to ≈4000 s (≈1.1 h). Beyond this point, the phoretically active interface is nearly depleted of fuel under illumination, leading to a significantly reduced, if not negligible, phoretic activity.

To estimate the separation time, we follow the approach outlined in previous discussions, assuming uniform particle motion along the streamline.^[^ ^64^ ^]^ The separation time is approximated using the equation from our previous publication; however, in the present case, the velocity difference arises from variations in height rather than size differences, as reported in reference.^[^ ^64^ ^]^ It is important to note that, in typical microfluidic separation processes, the expected separation time falls within the range of minutes. However, this duration depends on both the initial injected length of the particle mixture within the channel and the relative velocities of the two particle types.^[^ ^64^ ^]^ Given that microfluidic channels are generally short, as is the case in our experiments with a channel length of 1 cm, the separation time *t*_S_ can be determined by considering the velocity difference between the slow and fast particle fractions, *U*_S_ and *U*_F_, along with the initial distribution length *L* of the particle mixture (∼feed length) injected into the channel:

(1) $$t_{S}=\frac{L}{U_{F}-U_{S}}\;$$

This is demonstrated for a particle pair, typically faced according to well‐known silica chemistry for surface functionalization's (SiO_2_─OH, SiO_2_─NH_2_). Further particle pair type has the smallest measured velocity differences with *U*_F−_ *U*_S_  =  ∆*U*_diff._  =  18 µm s^−1^ (See Figures 5 and 6). Assuming the mixture feed length = channel length of 1 cm  =  10 000 µm (same microfluidic device used in all experiments), this yields a separation time of ≈9.3 min. The details of the calculations are given in Section S5 (Supporting Information). It is worth noting that reference ^[^ ^64^ ^]^ suggests, as a rule of thumb, that the separation length should be at least twice the channel length to achieve optimal separation performance. This is due to the influence of the lateral parabolic flow profile and the velocity distribution of individual particles, which can result in the broadening of particle fractions during motion. Factors such as particle activity and size distribution further contribute to this effect and should also be taken into account. Thus, the required time for such a particle pair type is 2∙9.3 min  =  18.6 – 20 min and will be shorter than all other demonstrated examples in Figures 5 and 6, as the data exhibit much bigger velocity differences. This means that the time to effectively separate *t*_S_ ≈ 20 min is much smaller before the phoretic active interface drains almost the entire fuel under illumination ≈1 h. In a similar fashion, one can minimize the mixture feed length to reduce the required separation time, too, of course, at the trade of reduced throughput quantity.

In other words, the activity of the PVPBMA remains sufficient to enable the complete separation of a binary particle mixture. If a longer release time is required, the PVPBMA interface can be further exposed to the aqueous solution of the photosensitive surfactant. The data presented in Figure S2d,f (Supporting Information) indicate that even after at least 3 h, the interface continues to adsorb surfactant at a significant rate, as saturation has not yet been reached. In practice, this can be achieved by extending the pre‐exposure time of the PVPBMA interface to the surfactant solution. Notably, no technical limitations are apparent that would prevent maintaining the interface in contact with the photosensitive surfactant until its final use in the separation process.

For example, such interfaces can be kept in the photosensitive surfactant for up to 9 days, but avoid longer durations due to the PVPBMA slowly peeling off in the surfactant solution (see Section S1.2.5, Supporting Information).

### 3.2 Particle Size Limit

**Figure 7:**
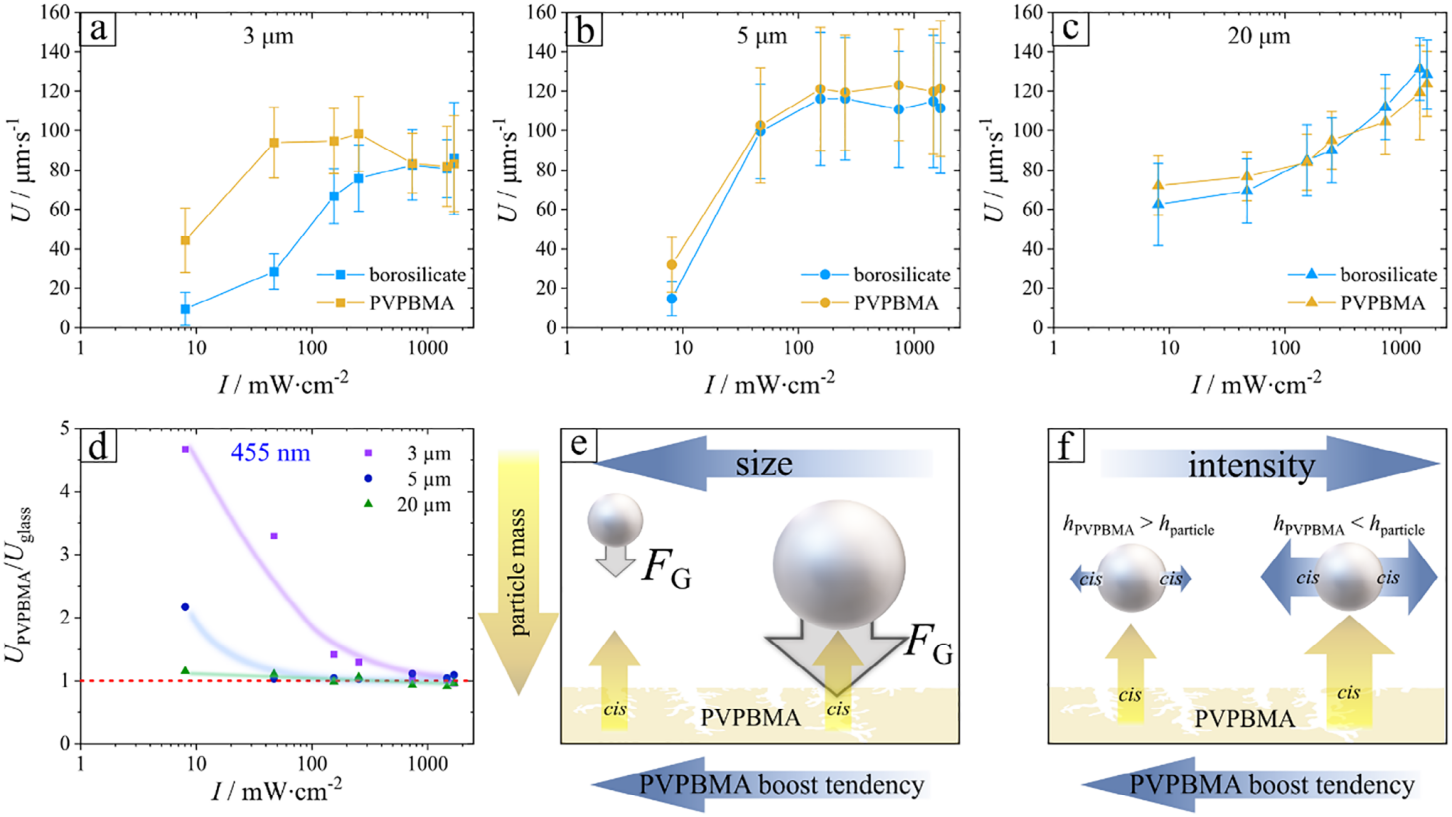
Mean velocity of porous silica microparticles (*P*‐SiO_2_) as a function of intensity (*λ*  =  455 nm) on glass (blue data) and polymer (orange data) surface at different particle sizes: a) *D*  =  3 µm, b) *D*  =  5 µm, c) *D*  =  20 µm. d) Relative mean velocity (*U*_PVPBMA_/*U*_glass_) as a function of intensity (*λ* = 455 nm). Illustration of measured trend in d for e) the particle mass and f) intensity dependence. Thus, the velocity of different sized porous particles (*D*  =  3, 5, and 20 µm) is recorded as a comparison between glass and PVPBMA in Videos S12–S14 (Supporting Information) at local blue light illumination (*λ*  =  455 nm, *I*  = 254 mW cm^−2^). Measurements were done with local light illumination. Video S12 (*D* = 3 µm), Video S13 (*D* = 5 µm), Video S14 (*D* = 20 µm).

Another important point to consider is the limit of boosting tendency for the effective particle size. Real samples normally contain particles with a distribution around an average particle size. This is important, due to the velocity of the flow streamline of sedimented particles is proportional to increasing particle size, while in contrast, the strength of hovering decreases with increasing size. This has already been demonstrated in our previous work.^[^ ^47^ ^]^ The same trend of hovering strength can be expected from the bottom wall and is validated from data displayed in Figure **7**, where we interpret that the mass (∼size) of the particle should be rather small. Here, the chemical activity of the porous microparticle can be sensitively controlled from the illumination intensity, where at a certain transition intensity, the height gain of the microparticle is dominated by the activity of the particle rather than by the activity of the PVPBMA. This can be interpreted by comparing the particl´s velocity for glass versus PVPBMA bottom substrate at fixed intensity. A summarized intensity dependence of *U* is displayed in Figure 7a–c and for a better visualization of the boosting tendency of the PVPBMA the normalized velocity gain *U*_PVPBMA_/*U*_glass_ is shown in Figure 7d.

Data exhibit in Figure 7d that the light‐induced motion boost from PVPBMA vanishes at critical intensity, where no significant boost (*U*_PVPBMA_/*U*_glass_ ≈1) mediated from the polymer layer. This appears at an intensity ≈200 mW cm^−2^ for *D*  =  3 µm and shifts toward lower intensities with increasing particle size. Furthermore, experimental data show that the impact of vertical displacement tendency of PVPBMA vanishes with increasing microparticle mass as the biggest particle have in average the same velocity on glass in comparison to PVPBMA. The sedimentation velocity reduced by drag in water scales much faster in comparison to the lift‐off velocity from the activity of the planar interface. This yields an equilibrium height as a function of the particle size as has been modelled in Figure 4b, where for values of *R*_P_ > 3 almost no boost is visible and with increasing tendency with increasing particle size (overlapping data points), in reasonable agreement with the experimental data in Figure 7.

### 3.3 Separation Quantity and Throughput

In order to achieve effective particle sedimentation, it is crucial to minimize the formation of multilayers. To prevent this, the particle concentration should be kept sufficiently low, not exceeding *c*_particles_ = 1.5 mg mL^−1^. As a result, the particle separation method leads to smaller quantities of particles being separated per unit time and volume. Under a flow rate of $\dot{V}=$150 µL min^−1^  =  0.15 mL min^−1^ (flow rate adjusted in this work) and maximum concentration as mentioned above yields a maximum throughput (mass of particles per time):

(2) $$\frac{m_{\mathrm{particles}}}{t}=c_{\mathrm{particles}}\cdot \dot{V}=1.5\frac{\mathrm{mg}}{\mathrm{mL}}\cdot 0.15\;\frac{\mathrm{mL}}{\min}=0.23\frac{\mathrm{mg}}{\min}$$

which is low, although better separation performance (less particle–particle interaction) should be done at lower particle concentrations. Based on empirical observations, we find that an optimal particle concentration is ≈0.5 mg mL^−1^. This concentration provides a simple and effective solution at the lab scale, particularly for applications such as the purification of surface‐coated microparticles post‐preparation. For higher throughputs, we do not foresee any technical limitations in stacking multiple flow cells in parallel. However, the separation process operates in a batch‐wise manner, as the PVPBMA substrate must be loaded with *trans*‐isomers after prolonged irradiation. This batch process may limit the feasibility of continuous operation, particularly when large quantities need to be separated, as would be required in industrial‐scale applications.

### 3.4 Recovery of the PVPBMA Interface

Note, that the photosensitive surfactant (Figure 1b) dynamically adsorbs at polymeric interfaces,^[^ ^51^ ^]^ then, after the separation, the photosensitive surfactant can be “recovered” by switching the isomer ratio into the *trans* dominating ratio to be ≈90% with green light (*λ* = 530 nm) ≈10 min of illuminated (depends on intensity)^[^ ^62^ ^]^ and subsequent immersing the PVPBMA interface with recovered *trans*‐isomer solution over longer time (≈1 h) the PVPBMA will be loaded again (see Figures S2–S4, Supporting Information). This is also supported by contact angle measurement, where a recovery could be achieved with green illumination, too (see Section S1.2.3 and Figure S6, Supporting Information).

### 3.5 Durability of the PVPVMA Interface

PVPBMA is completely insoluble in water and remains adhered to the glass substrate, whether in aqueous solutions containing dissolved surfactants or in pure Millipore water over extended periods. The adhesion durability of PVPBMA to the glass substrate has been validated, with experimental details provided in the Supporting Information (Section S1.2.5, Supporting Information). In brief, neither water nor an aqueous surfactant solution (*c*  =  1 mM) causes the PVPBMA to dissolve or detach from the glass over a prolonged period (≈5 days). After this time, the PVPBMA begins to peel off gradually from the edges of the glass substrate when exposed to surfactant solution. However, a sufficiently large area remains adhered to the substrate, adequate for coating a microfluidic channel.

### 3.6 Practical Implications

We would like to emphasize that the separation process requires minimal and inexpensive equipment, including an LED light source (wavelength range 365–490 nm), a pump, a low‐concentration surfactant solution (*c*  =  1 mm), and the PVPBMA interface. It is important to note that the PVPBMA interface is not a limiting factor, as the polymer is cost‐effective, and the preparation procedure involves simple spin coating onto disposable glass substrates. Only small quantities (∼mg) of the polymer are needed for coating, making the process both economical and efficient. As a result, such coated glass slides can be considered disposable and can be easily and quickly fabricated on demand. Furthermore, the PVPBMA dissolves in many organic solvents. Thus, prior to separation, particles should be dispersed in organic‐free solvents and must be transferred into ionic‐free water (MilliQ) with dissolved photo surfactant. The *l*‐LDDO very sensitively depends on the ionic strength and breaks down already at a salt concentration of 1 mm NaBr,^[^ ^65^ ^]^ therefore, avoid salt concentration by washing the particles multiple times. Note that the surfactant concentration should be 1–2 mm minimum.

### 3.7 Temperature Limitations

Usually, adsorption enthalpy of surfactants is negative (∆*H*_ads_ < 0), which means that adsorption releases heat. In consequence the negative ∆*H*_ads_ causes a decrease in the adsorption with increasing temperature. Thus, we expect the activity of PVPVMA to decrease with increasing temperature. Yet, in our previous publication, we demonstrated that separation by using phoretic activity to levitate microparticles is at least possible up to temperatures of 45 °C.^[^ ^66^ ^]^ Thus we assume that small temperature fluctuations (∆*T*  =  ±2 °C) in laboratory will not have great impact on the sensitivity of separation.

## 4 Conclusion

In previous work,^[^ ^47^, ^48^ ^]^ we have shown that an efficient chromatographic‐like microfluidic flow separation by surface properties of a binary particle mixture is possible for particles that load an azobenzene‐containing surfactant undergoing a reversible photo‐isomerization under suitable light. The feasibility depended crucially on: i) the particles (at least one species) loading relatively large amounts of surfactant in order to achieve a significant chemical activity; and ii) the phoretic mobility (the surface‐properties dependent response to the local chemical‐composition of the solution) being sufficiently different between the particles. Here we demonstrate that the scope of the method can be dramatically improved by removing the strong limitation expressed in i) above by shifting the loading with the photosensitive surfactant to the wall of the channel, i.e., making a chemically active wall.

This key component is obtained by spin coating the (glass) wall with a rough, micro‐ and nano‐porous PVPBMA layer, which absorbs (and stores) in its hydrophobic pores massive amounts of the more surface active *trans*‐isomer and, under suitable wavelength illumination, exhibits a sustained steady release of the more hydrophilic *cis*‐isomers into (while reloading trans‐isomers from) the bulk solution. We have demonstrated this active wall feature by vertically displacing, for significant elevations above the wall, sedimented compact particles that otherwise (i.e., at a non‐active wall, such as a glass one) would not exhibit vertical displacements owing to their minute loading of the surfactant. Furthermore, we have also shown that the chemical activity (rate of release of *cis*‐isomers) of the PVPBMA can be precisely controlled through the intensity and wavelength of the applied illumination.

Since in general both the particle and the wall will turn chemically active under illumination, the vertical displacement of the particles will synergistically combine this double induced chemical activity (particle interface and the bottom PVPBMA interface), which, we argued theoretically and verified experimentally, should yield an amplified hovering height against the sedimentation tendency. This translates into exploring the larger velocity region of the shear microfluidic flow, and thus provides the necessary means for a significantly improved (especially important for weakly active microparticles) separation, as we demonstrated experimentally.

In fact, we have obtained a superior technical tool (simple in performance and operational cost), with a much improved versatility (in comparison to what has been recently reported in Ref. [47]) toward interfacially sensitive separation of microparticles. The PVPBMA coating enables a very versatile method for the separation of a large variety of “rather inactive” microparticles, which on average is more probable in comparison to the separation of “active” versus “inactive” microparticle pairs.^[^ ^47^ ^]^ Our method offers the means for interfacial‐sensitive separation technologies suitable for particles that are almost identical in size and in the core, but not in the interfacial properties. Moreover, it benefits from a simple, practical, low‐cost operation only requiring an LED light source, a pressure‐driven flow within a microfluidic channel, the photosensitive surfactant, and the spin‐coated PVPBMA wall. Future work will explore several avenues to optimize key aspects of the system. This may include, under localized light collimation: 1) the use of different illumination patterns, 2) the exploration of various photosensitive surfactants to enhance the loading and release capacity and rate of the PVPBMA, and 3) the investigation of alternative polymer bottom substrates. Particularly, the latter holds significant potential for improving light‐switchable active interfaces.

## 5 Experimental Section

### Materials

*Chemicals*: Methanol (standard grade) was purchased from Sigma–Aldrich Chemie GmbH and used without any further purification. Poly (4‐vinylpyridine‐co‐butyl methacrylate) was purchased from Sigma–Aldrich Chemie GmbH, Germany, and contains a 90% 4‐vinylpyridine content. No details about the molecular weight are given by the supplier, thus, the molecular weight is declared as unknown.

*Meso Porous silica colloids* in different sizes of *D* = (3 ± 1) µm,*D*  =  (5 ± 1) µm, and *D* = (20 ± 5) µm, and nonporous (so‐called compact) silica colloids of *D*  =  (4.0 ± 0.24) µm or (5.0 ± 0.2) µm are purchased from Micromod and micro‐Particles GmbH (Germany). Mesoporous silica colloids of average (N5 ± 1) µm diameter with amino functionalization (─NH_2_) were purchased from the company Micromod. Polystyrene particles with a diameter *D*  =  (5.0 ± 0.10) µm, either with interfaces of plain, amino functionalized with primary amines (─NH_2_) and quaternary amines (─NR_3_^+^), octadecyl (─C_18_) and carboxy (─COOH) surface functionalization morphology were purchased from micro‐Particles GmbH (Germany).

The aqueous dispersion of colloids was mixed with surfactant stock solution at a concentration of 1 mm and equilibrated for at least 24 h before measurements. A microfluidic flow chamber µ‐slide^VI^ with a glass bottom cover slip or no bottom cover slip (Ibidi GmbH) of a volume of 40 µL was used in order to provide a closed environment. The channel with no bottom cover slip was closed by gluing a PVPBMA (on a glass support) cover slip. When an aqueous dispersion of azobenzene containing surfactant and porous or nonporous silica colloids was placed in a chamber, the colloids sedimented down to a glass or PVPBMA surface. The chamber was connected to a syringe pump (Harvard Apparatus Company, PhD Ultra). All samples were kept in the dark or in red light to prevent unwanted photo‐isomerization. The measurements are conducted at a room temperature of *T * =  23 °C.

### Sample Preparation

Aqueous dispersions of plain (SiO_2_) and porous silica (PSiO_2_; pore diameter: 6 nm) microparticles of *D* = 3 µm (acquired from micromod Partikel Technologie GmbH, Rostock. Germany) (see Figure 1) were mixed with 1 mm photosensitive surfactant solution above cmc (cmc_surfactant_ = 0.5 mM).^[^ ^67^ ^]^ Millipore water was used to prepare the aqueous solutions. Samples were prepared at 0.3 mg mL^−1^ and kept for at least 24 h for equilibration. A µ‐slide^VI^ open chamber is used (ibidi GmbH, Gräfelfing. Germany, *η_D_* = 1.523). The PVPBMA bottom cover slip is glued from the bottom to provide a closed environment. The chamber is connected to a syringe pump from Harvard Apparatus Company (PhD Ultra). All samples were kept in the dark or red light (*λ* = 625 nm) to prevent undesired photo‐isomerization. The flow rate used in all the experiments was 150 mL min^−1^.

### Azobenzene Containing Surfactant

The azobenzene containing trimethyl‐ammonium bromide surfactant, full name 6‐[4‐(4‐Hexylphenylazo)‐phenoxyl‐butyl‐trimethylammoniumbromide and abbreviated C_4_‐Azo‐OC_6_TMAB, was synthesized as described elsewhere.^[^ ^68^ ^]^ The surfactant **(Figure** 1b) consisted of a spacer of 6 methylene groups between the positively charged trimethyl‐ammonium bromide head group and the azobenzene unit with butyl tail attached. Water (Milli‐Q system) with a specific resistance larger than 18 MΩ·cm was used to prepare aqueous solutions. Optical and isomerization properties of the same substance are reported in detail elsewhere.^[^ ^62^ ^]^

### PVPBMA Interface Preparation

The polymer, Poly (4‐vinylpyridine‐co‐butyl methacrylate) (90% 4‐vinylpyridine content) was dissolved in methanol in a quantity 1 g of polymer in 10 mL of methanol. After fully dissolving for over 1 day, a glass slide (microscope glass slide) was coated with the PVPBMA Polymer Films: The Films were prepared via spin casting (spin coating) onto the planar glass interface (micro glass slide).^[^ ^69^ ^]^ The coating parameters were chosen according to achieve typical film thicknesses of 0.1 mm. The commercial PVPBMA (1 g) was dissolved in methanol (10 mL). The viscous solution of ≈250 µL was first deposited over the entire planar support and then spin‐cast at 1000 rpm (∼angular velocity ω  =  105 rad s^−1^) for 1 min. The ramping rate was set at 1000 rpm s^−1^ (∼angular acceleration α  =  105 rad s^−2^). Then the planar substrate was spin‐cast at 3000 rpm (∼ω  =  315 rad s^−1^, ramping rate  =  3000 rpm ^−1^s (∼α  =  315 rad s^−2^)) for 1 min to dry the PVPBMA. Then the coated glass slides were kept in the air for at least 1 h to ensure the final evaporation of the remaining methanol, and the glass slide were used for further investigations. For microfluidic experiments the coated PVPVBMA‐glass slide was directly glued as a bottom substrate for the commercial microfluidic chamber.

Spin coating was performed at a room temperature of *T*  =  23 °C. This yielded a transparent coated glass slide, with ≈1 mm‐thick PVPBMA layer. Then the coated glass slide was placed on a commercial µ‐slide^VI^ (Ibidi) without bottom cover. The homogeneity of porosity distribution from the center toward the edge of the glass slide is demonstrated in Video S15 and details are Section S1.2.2 (Supporting Information). Characterization of the surfactant adsorption tendency is given in Section S1.2 (Supporting Information) and following.

### Quartz Crystal Microbalance with Dissipation (QCM‐D)

QCM‐D measurements were performed with a single window chamber Q‐sense instrument (Biolin Scientific) using commercial Q‐sense crystals coated with borosilicate (QSX336, LOT Quantum Design GmbH). For the poly (4‐vynilpyridine‐co‐butyl methacrylate) coating, the borosilicate crystal was spin coated following the procedure described in the previous subsection. A typical measurement was performed both in an aqueous solution (for removing the artifact “light induced detuning” from UV light illumination, for plain borosilicate interface)^[^ ^70^ ^]^ and in an aqueous solution of azo‐benzene containing surfactant (*c* = 1 mm). Prior to each measurement, a flow (50 µL min^−1^) of degassed Millipore water was injected into the measurement chamber until a stable baseline for the values of the frequency shift (Δ*f*) and the dissipation shift (Δ*D*) was reached for 15 min. Subsequently, the baseline was reset by starting a new measurement, where data monitoring of Δ*f* and Δ*D* was done for third to ninth overtone number *n*. While changing the injection solution, the flow was shortly interrupted to avoid any potential air bubble soaking. When performing measurements under light illumination, the flow was switched off. The light source was perpendicularly collimated into the window chamber (the details of the measurement setup are described in Ref.[70]). The data analysis was done on the third overtone number, the recorded values of Δ*f(t)* and Δ*D(t)* being converted into an areal mass density (*m*/*A*) via

(3) $$\frac{m\left(t\right)}{A}=-\frac{C}{n}\cdot \left(\Delta f\left(t\right)+\frac{f_{C}\cdot \Delta D\left(t\right)\cdot 10^{6}}{2}\right)$$

where *C* = 17.7 ng cm^−2^ is the apparent crystal constant and *f*_c_ the eigenfrequency of the quartz crystal (*f*_c_  =  4.95 MHz). The mass density was converted into an areal number density (*n*/*A*) using the known molecular weight *M*_W_ of the adsorbent and Avogadro constant *N*_A_ via:

(4) $$\frac{n}{A}=\frac{m\cdot N_{A}}{A\cdot M_{W}}\;$$

For the experiments involving light illumination, the artifact “light induced detuning” (LID) was removed by correcting the areal mass density to^[^ ^70^ ^]^:

(5) $${\left[\frac{m\left(t\right)}{A}\right]}_{\mathrm{corrected}}={\left[\frac{m\left(t\right)}{A}\right]}_{A\mathrm{zo},\mathrm{light}}\;-{\left[\frac{m\left(t\right)}{A}\right]}_{H2O,\mathrm{light}}$$

where ${\left[\frac{m(t)}{A}\right]}_{A\mathrm{zo},\mathrm{light}}$ and ${\left[\frac{m(t)}{A}\right]}_{H2O,\mathrm{light}}\;$ are the areal mass densities from the surfactant solution and Millipore water, respectively.

### UV–Vis Measurements

UV–vis spectroscopy was performed using a Cary 5000 UV–vis–near‐infrared (NIR) spectrophotometer instrument (Agilent Technologies, USA). For liquid solutions, a rectangle quartz cuvette (from Helma Analytics) was placed in the monitoring beam. For PVPBMA interface, the glass slide with the coated interface was placed in the monitoring beam. A light source of different wavelengths was placed perpendicular near the sample holder with the illumination light facing toward the sample holder. Data were recorded for three different wavelengths (*λ*  =  490 nm, *λ*  =  455 nm, *λ*  =  365 nm). To demonstrate adsorption of the azobenzene containing surfactant on the polymer surface, a glass slide was spin coated with a PVPBMA solution. The polymer coated slide was submerged in an azobenzene containing surfactant solution (*c*  =  0.5 mm) and then washed with double distilled water. The absorption spectrum of the pure polymer coated slide, and of the polymer coated with azobenzene containing surfactant solution slide is shown in Figure S7b (Supporting Information). The spectra corresponding to the azobenzene containing surfactant solution before the immersion of the polymer coated slide and after the withdraw of the slide, respectively, are shown in Figure S7c (Supporting Information).

### Atomic Force Microscopy

AFM measurements were carried out using an NTEGRA AFM (NT‐MDT) operating in tapping mode with a scan rate of 1 Hz per frame in air. The pixel resolution was set to a value of 512 × 512 pixel with a scan size of (20 × 20) µm. Commercial tips (Nanoworld‐Point probe) with a resonance frequency of 320 kHz and a spring constant of 42 N m^−1^ were used. The experimental raw data was analyzed using the software Gwydion (version 2.58), with the data processed in the following order: I) mean plane subtraction, II) align rows using a polynomial algorithm with polynomial degree of 2, III) shifting minimum height value to zero, and IV) setting imaging height scale range from 0 to 100 nm. To calculate the cross‐section height the AFM micrographs are analyzed over a thickness of 4 pixel.

### Scanning Electron Microscopy, SEM

SEM was carried out for the characterization of both the porous and the compact SiO_2_ microparticles. A scanning electron microscope Ultraplus 4061, Zeiss, Germany, with magnification of 20 000×, was used.

### Contact Angle Measurements

For contact angle measurements, the surface (PVPBMA on a micro glass slide or micro glass slide) and aqueous droplet solution were observed (either pure MilliQ‐water or MilliQ‐AzoC_6_ solution (*c*_Azo_  =  1 mm)) simultaneously from the top and the side. The top view was used to observe the circularity of the drop footprint and overall contact line motion during the experiment, indicating pinning and wetting defects that possibly influence the measurement. The telecentric side view (0.50 × SilverTL Telecentric Lens, Edmund Optics Ltd., U.K., and a 4.2 MPix CMOS Camera MQ042MG‐CM XIMEA GmbH, Germany) images the cross section of the droplet and gives a distortion‐free depiction of the droplet contour. The image acquisition was done at red light (*λ*_top_  =  660 nm, *λ*_side_  =  680 nm), which caused no photoisomerization of the photosensitive surfactant. The typical drop sizes were set below capillary length to neglect the influence of gravity. In such a way, the contact angle could be determined with a typical error of ≈ ± 0.1°. To minimize evaporation effects, the sample chamber was kept under a laminar flow of saturated N_2_/H_2_O vapor. Every contact angle measurement was dynamically measured (the droplet volume was slightly increased with the connected syringe till the contact line moved, and then the advancing contact angle was measured, and the equilibrium value was taken. The contact angle was determined by extracting and fitting the contour with an elliptical fit and excluding the influence of the connected syringe using a home‐built software. Additionally, for the surfactant solution, the contact angle was measured without light illumination, under UV light irradiation with a wavelength of 365 nm (at an intensity of 10 mW cm^−2^), and under irradiation with green light @ 520nm.

### Optical Microscopy

An inverted microscope Olympus IX73 equipped with a LED light source (Thorlabs GmbH) of various wavelengths, UV (*λ* = 365 nm), blue (*λ* = 455 nm), (*λ* = 490 nm), and red (*λ* = 625 nm), was used in all the measurements. LED light sources, if locally collimated, were guided from collimation through the objective, and if globally, through the ocular. Details of experimental setup can be extracted from Supporting Information for local collimation^[^ ^71^ ^]^ and for global collimation.^[^ ^72^ ^]^ The illumination power was directly measured for each intensity power by using an optical power meter PM100D with a sensor S170C (Thorlabs GmbH, Germany). The intensities applied for this study were:

‐ UV (*λ* = 365 nm): from 98.8 to 0.18 mW cm^−2^;

‐ blue (*λ* = 455 nm): from 1691.5 to 8.0 mW cm^−2^; and

‐ green (*λ* = 490 nm): from 339.8 to 0.2 mW cm^−2^.

Time resolved micrographs were recorded with a CCD camera (Hamamatsu ORCA‐Flash4.0 LT (C11440) at 30 frames per second.

### Video Microscopy Data Analysis

The recorded videos were decomposed into single‐frame images and converted into binary pixel information (known as thresholding), subjected to a subsequent particle detection involving a complex thresholding and analyzing procedure described elsewhere.^[^ ^47^ ^]^ In short, contour extraction was done over a series of three thresholding steps, where the first two steps refer to a brightness thresholding in order to separate background from particles, and in the last step, the modified images were processed to detect the center of mass of particle contours by their area. The center of mass served as a location coordinate for object tracking frame‐to‐frame via retrieval of the minimum distance for each individual particle to calculate the trajectory and momentum velocity by multiplying the travelled distance by the frame‐rate. The average velocity $\bar{U}$ (mean, median) and the velocity standard deviation (see Statistical Analysis Section) were then calculated from individual trajectories to display the data as a function of time. The image processing, the object tracking, and the statistical analysis were carried out in Python by using the software packages Bokeh, Numpy, OpenCV‐python, Matplotlib, Openpyxl, Pandas, and SciPy.

Data plotting, data fitting were done with commercial software Origin 2019b.^[^ ^73^ ^]^

### Voronoi Diagram

The recorded video was decomposed into an image series, and the data were calculated into a binary pixel information (black and white) by the standard “thresholding algorithm” already implemented in the image software Fiji. Then the polygons were calculated for Voronoi diagrams emerging from the locations of centers of particles using the Voronoi algorithm from the Fiji software. The images with calculated Voronoi tessellations contain a gray scale. Thus, for better visualization, the images were calculated into black and white and the image series was converted as a recorded video file.

### Statistical Analysis—*Time resolved average velocity* $\bar{U}$

The sample size *n* (number of particles) of individual velocity *U*_i_ for each frame‐to‐frame was averaged using the arithmetic mean to obtain $\bar{U}$:

(6) $$\bar{U}=\frac{1}{n}\;\left(\sum_{i=1}^{n}U_{i}\right)$$

the corresponding standard deviation *σ* was calculated from the square root of the variance for the sample size of *n−*1:

(7) $$\sigma =\sqrt{\frac{1}{n-1}\sum_{n=i}{\left(U_{i}-\bar{U}\right)}^{2}}\;$$

The average sample size per frame was calculated from a number of trajectories in the frame it as mentioned in the figure caption or tabulated in the Supporting Information.

### Theoretical Modeling—Model system

The system of interest was modeled as a dilute, ideal solution (solvent and solute molecules) occupying the half space *z*  >  0 above a planar wall located at *z*  =  0. The system was in contact with a reservoir which fixes the temperature and the bulk chemical potentials of the components; under the assumption of an ideal solution, this implied that the average density of solute far from the wall (in the bulk) was fixed to a constant value that we denote by *C*_∞_. On the wall, there was an infinitesimally thin, chemically active patch (see Figure 4a). The chemical activity of the patch was modeled as a source (or sink) of solute, i.e., at the patch, solute molecules were released (removed) from the solution. The solute was assumed to diffuse freely in the solution with a diffusion constant *D*, and to be well described as an ideal gas. It was further assumed that the solute was efficiently transported away from the patch toward the bulk solution, i.e., any possibility of a patch poisoning was disregarded. Accordingly, the activity of the patch is characterized by a time‐independent rate *Q* (strength of the source/sink) of release of solute molecules per unit area of the patch; for simplicity, *Q* was further assumed to also be spatially constant over the area of the patch.

In the current experiments, the patch refers to the illuminated area of the surfactant‐releasing polymer film, the solvent comprises the water and the *trans* molecules of surfactant, and the “solute” refers to the *cis* molecules of surfactant, which were released from the illuminated patch. It was assumed that the *trans* molecules of surfactant were present in abundance in the solution, and thus their absorption into the polymer while the illumination is on is neglected, i.e., the *cis* isomer remained the only species of interest for the activity of the wall and the phoretic response of the particles. (Same assumptions were applied regarding the eventual self‐activity of the particles, in particular for porous ones.)

Because of the presence of the active patch, which perturbed the distribution of solute in solution from the equilibrium one dictated by the reservoir (the bulk solution, which maintains a homogeneous *C*_∞_ far from the wall), the system was driven out of equilibrium. In the following, it was assumed that a steady state of the fluid solution, characterized by a distribution *c*(**s**) of solute (where **s** denotes a point within the solution, see Figure 4a), emerged, and it was aimed at being determined. The analysis of the case that the patch has circular symmetry was restricted and, thus, the whole system had axial symmetry; accordingly, an axisymmetric solution (which, in terms of the cylindrical coordinates, will depend on *r* and *z*, but not on the azimuthal angle) was looked for. A colloidal particle immersed in this solution will experience, in addition to the sedimentation due to its apparent weight, a phoretic drift

(8) $$V_{ph}\left(s\right)\;≔b\nabla c\left(s\right)$$

which is due to being exposed to the inhomogeneous distribution *c*(**s**) of solute.^[^ ^74^, ^75^ ^]^ The *z*‐component of this phoretic drift will induce a vertical displacement (away from/toward the wall if the phoretic coefficient *b*  > /  <  0) when the patch was active; this will manifest in the particle experiencing a different region of the externally driven shear flow compared with the one it experiences in the absence of activity. In other words, the activity of the wall will manifest into a different drift velocity along the *x* direction. This is discussed and calculated in the next section.

### Theoretical Modeling—Solute Distribution

Since the solute was conserved in the solution at *z* > 0, its distribution at steady state obeys ∇ · **j**  =  0 in terms of the particle current **j**. Under the assumption that the diffusion of the solute was fast while the hydrodynamic flow was slow, such that the transport of the species by diffusion dominates over the one by advection (i.e., the Péclet number is very small), the current of solvent was diffusive, **j**  =   − *D*∇*c*. After taking the divergence, one infers that the distribution of solute at steady state obeys the Laplace equation.

(9) $$\nabla^{2}c\left(s\right)=0$$

The solute distribution was subjected to the boundary conditions at infinity (bulk solution),

(10) $$c\left(\left|s\right|\to \infty \right)=C_{\infty }$$

and on the wall, located at *z*  =  0, that contains the chemically active patch,

(11) $$\left\{n\cdot \left[-D\nabla c\left(s\right)\right]\right\}{|}_{z=0}=QI\left(r\right)$$

respectively. In Equation (11), **n** = **e_z_** denotes the inner normal (into the fluid) at the wall, while *I*(**r**) denotes the in‐plane indicator step function (unity at the patch, vanishing outside the patch) which described the position, shape, and extent of the patch. Equation (11) defines the chemical activity of the patch (release/removal of solute) as a diffusion current, along the normal direction, into the solution, the value of which is prescribed to be equal to the rate *Q* of the chemical activity of the patch (accordingly, *Q*  >  0 corresponds to a source, while *Q*  <  0 to a sink).

Although in the experiment the illuminated area is a rectangular‐shaped one, here the case of the patch being a circular disk of radius R is focused, the center of which was picked as the origin of the system of coordinates (see Figure 4a), i.e.,

(12) $$I\left(r\right)=\left\{\begin{array}{c} 1,\;r\;\leq \;R\\ 0,\;r\;>\;R \end{array}\right.$$

There were no major differences to be expected, with the condition that only the plane of symmetry *z* − *x* was considered in the analysis of the drift by the shear flow and the vertical displacement of the particle by phoresis.

For the above choice of shape, the solution *c*(**s**) of the boundary‐value problem in **Equations** ((9), (10), (11)) was obtained by adapting the solution to an electrostatic problem with a similar mathematical structure from Ref.[76] (Ch. 3.2.3–4), as

(13) $$c\left(r,z\right)=C_{\infty }+C_{0}\int_{0}^{\infty }\frac{d\xi }{\xi }J_{1}\left(\xi \right)J_{0}\left(\xi \frac{r}{R}\right)\exp\left(-\xi \frac{z}{R}\right):=C_{\infty }+C_{0}\bar{c}\left(r,z\right)$$

where J_0,1_ denote Bessel functions of the first kind^[^ ^77^ ^]^ and
(14) $$C_{0}:=\frac{QR}{D}$$

is a characteristic number density. The last equality in Equation (13) defines the dimensionless density $\bar{c}\left(r,z\right)$, which involves only the lengthscale *R*, the radius of the patch; this is shown in Figure 4a, where, for convenience of comparing with experimental recordings, the spatial coordinates are measured in units of particle radii.

### Theoretical Modelling—Phoretic flow

The phoretic velocity experienced by a particle follows from Equations (8) and (13) as

(15) $$\begin{array}{ccc} V_{\mathrm{ph}}\left(s\right)=b\nabla \bar{c}\left(r,z\right) & = & -V_{0}\beta [e_{r}\int_{0}^{\infty }d\xi J_{1}\left(\xi \right)J_{1}\left(\xi \frac{r}{R}\right)\exp\left(-\xi \frac{z}{R}\right)\\ & & +e_{z}\int_{0}^{\infty }d\xi J_{1}\left(\xi \right)J_{0}\left(\xi \frac{r}{R}\right)\exp\left(-\xi \frac{z}{R}\right)] \end{array}$$

where β :   =  *b*/|*b*| and the velocity scale is given by

(16) $$V_{0}:=\frac{Q\left|b\right|}{D}$$

based on the experimentally observed velocities for active porous silica particles, the expectation is that the magnitude |*V*_0_| is in the order of a few micrometers per second. Note that both *V*_0_ and **V**_ph_(**s**) are independent of the radius of the particle; the dependence on the particle is only in terms of material properties encoded by the phoretic coefficient *b*. In the following β  =   − 1, is used, which corresponds to motion against the gradient in density of solute (away from region of large density), and thus to an effective repulsion of the particle by the active wall. The motion of a particle experiencing solely the phoretic drift by Equation (15) is shown by the white streamlines in Figure 4a.

### Theoretical Modelling—Sedimentation and Hovering

The sedimentation velocity of a spherical colloidal particle of radius *R_p_* with its center located at *z* was obtained from the Stokes formula (overdamped motion of colloids in a Newtonian fluid at zero Reynolds number) as,

(17) $$\begin{array}{ccc} V_{s}\left(z\right)=-e_{z}\frac{\frac{4\pi }{3}\left(\rho -\rho_{W}\right){R_{p}}^{3}g}{6\pi \mu R_{p}}\gamma \left(z\right) & & =-e_{z}\frac{2\left(\rho -\rho_{W}\right){R_{0}}^{2}g}{9\mu }{\left(\frac{R_{p}}{R_{0}}\right)}^{2}\gamma \left(z\right)\\ & & =:-e_{z}{V_{s}}^{\left(0\right)}{\left(\frac{R_{p}}{R_{0}}\right)}^{2}\gamma \left(z\right) \end{array}$$

where *µ* denotes the viscosity of the solution, *ρ* and ρ_*W*_ denote the mass densities of the particle and of the solution, respectively, *g* is the gravitational constant, *R*_0_ is some auxiliary particle radius (its sole purpose is to define the velocity scale *V_s_* ^(0)^), which here is taken *R*_0_ =  1 µm, and γ(*z*/*R_p_*) is the height‐dependent mobility for motion in the direction normal to the wall. An analytical expression for γ(*z*) as a series in (*z*/*R_p_*) is available;^[^ ^56^ ^]^ for the discussion here, the important thing is that it is a monotonically increasing function, with the limiting values γ(*z* → *R_p_*)  → 0 and γ(*z* ≫ *R_p_*)  → 1, and that the increase from zero is steep: γ(*z*  =  10*R_p_*) ≃ 0.98, i.e., by *z*/ *R_p_* =  10 the mobility function is within 2% of its value far from the wall. The sedimentation velocity scale,

(18) $${V_{s}}^{\left(0\right)}=\frac{2\left(\rho -\rho_{W}\right){R_{0}}^{2}g}{9\mu }$$

depends only on the material properties of the particle (its mass density). For the particle of interest, silica (*ρ * =  2300 kg m^−3^) and porous silica (*ρ * =  2000 kg m^−3^), and with *R*_0_  =  1 µm, it renders

(19) $${V_{s}}^{\left(0\right)}\approx \left\{\begin{array}{c} 2.9\;\mu m\;-1s,\;silica\;particles\\ 2.2\;\mu m/s,\;porous\;silica\;particles \end{array}\right.$$

### Hovering for Compact Particles

For a compact particle, the phoretic repulsion from the active wall and the sedimentation toward the wall were the only components for the motion along the vertical direction; for porous particles, additional contributions can occur due to the self‐generated gradients in the *cis* surfactant, as discussed and analyzed in the previous paper.^[^ ^47^ ^]^ Here, the compact particles were focused on, and the porous particles case was left for the next subsection.

Because the sedimentation velocity vanishes at the wall (*z*/ *R_p_* =  1), where the normal component of the phoretic velocity is non‐zero, while the phoretic velocity vanishes far from the wall, where the sedimentation velocity is non‐zero, there will always be a hovering position (the point *z*/*R_p_* of mechanical equilibrium between the phoretic and sedimentation velocities). Because the phoretic velocity is independent of the particle size, while the sedimentation does depend strongly on the size (it increases quadratically with the radius, see Equation 16), the hovering height will be farther from the wall for the smaller particles than for the larger ones. Furthermore, the sedimentation velocity is independent of the lateral position *r*, while the vertical component of the phoretic velocity depends on the lateral position and is maximum (by symmetry arguments, see Figure 4a,b) along the axis of symmetry *Oz*. (Note though, as shown in Figure 4a, for patches of a size much larger than the size of the particle at the relevant heights *z*/*R*p ≈1–10 the phoretic velocity is quasi‐vertical almost everywhere above the patch; thus for the experimental realization in which we are interested the arguments below hold at all relevant *r*, not only at *r* = 0) Accordingly, the equation is looked at for the hovering height only along this axis, where calculations are greatly simplified by the fact that a closed form expression for ***V***_ph_ is available. Along the *Oz* axis, **V**_ph_ has only a z component, which is given by

(20) $$\begin{array}{ccc} -V_{ph}\;\left(r=0,z\right) & = & V_{0}\;\int_{0}^{\infty }d\xi J_{1}\left(\xi \right)\;\exp\left(-\xi \;\frac{z}{R}\right)=\\ & & V_{0}\;\left(1-\frac{1}{\sqrt{1+{\left(R/z\right)}^{2}}}\right)=:\;V_{0}u_{ph}\left(z\right) \end{array}$$

Combining this with Equation (14) renders the following equation for the hovering heigh *z*  = *h_ac_* :

(21) $$u_{ph}\;\left(h_{ac}\right)=\frac{{V_{s}}^{\left(0\right)}}{V_{0}}\;{\left(\frac{R_{p}}{R_{0}}\right)}^{2}\gamma \left(h_{ac}\right)\Rightarrow \frac{{V_{s}}^{\left(0\right)}}{V_{0}}\gamma \left(h_{ac}\right)\approx {\left(\frac{R_{0}}{R_{p}}\right)}^{2}$$

where in the last equation we used that *h_ac_*/*R* is expected to be very small, of the order of 0.1, for patches of large size *R*, and thus *u_ph_* (*h_ac_*) ≈ 1.

### Hovering for Porous Particles

In the case of porous particles, an additional contribution *V*_*sm*_ to the motion along the vertical direction occur due to the fact that the particle itself was chemically active, which led to self‐motility in the vicinity of a wall. This contribution was calculated by Ref. [47] in terms of two functions, *f_p_* and *f_w_*, of the height *z* above the wall (in units of the particle radius) as

(22) $$V_{sm}\;\left(z\right)=-e_{z}V_{0}^{\prime }\left(\beta f_{p}\left(z\right)+\beta_{w}f_{w}\left(z\right)\right)$$

Here β_*w*_ denotes the phoretic mobility of the wall (for the glass wall it is expected that β_*w*_ =  β (and equal to −1), but for the polymer‐coated wall it is more probable that β_*w*_  ≠  β, though still remaining negative), while and the characteristic velocity *V*′_0_ is given by an expression similar to that for *V*_0_, Equation (16), but with a possibly different rate *Q*′  ≠  *Q* of solute release. Thus, the self‐motility contribution brings in two more unknown parameters, *V*′_0_ and β_*w*_, in the analysis of the emerging active hovering. The hovering height above an inert wall (e.g., the case of the glass wall) is then obtained as the solution of

(23) $$-\;\left[\beta f_{p}\left(h_{ac}\right)+\beta_{w}f_{w}\left(h_{ac}\right)\right]=\frac{{V_{s}}^{\left(0\right)}}{{V^{\prime }}_{0}}\;{\left(\frac{R_{p}}{R_{0}}\right)}^{2}\gamma \left(h_{ac}\right)$$

while above an active wall (e.g., the porous polymer‐coated wall) was obtained as the solution of

(24)

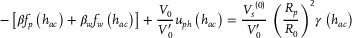




The solution of Equation (18) is a function of various parameters; the results by using experimentally motivated estimates for *V_s_* ^(0)^, *V*′_0_ and *V*_0_, and by fixing β  =  β_*w*_  =   − 1 (see the Section S4, Supporting Information) are illustrated in Figure 4b.

The solution of this equation for *V*′_0_  =  10 *V*_0_ ≫ *V*_0_ and *k* = 1 is shown in Figure 4b.

## Conflict of Interest

The authors declare no conflict of interest.

## Author Contributions

N.L. synthesized surfactant. D.V.M., A.S., S.L., F.R., I.M., J.R.B, S.E. and P.O. performed measurements and data analysis. M.B. conceived and organized the project. M.N.P. developed the theoretical modeling and suggested the possibility of fractionation for generically weakly active particles. D.V.M., S.S., M.N.P., and M.B. wrote and edited the manuscript. All the authors were involved in the preparation of the manuscript. All the authors have read and approved the final manuscript.

## Supporting information



Supporting Information

Supplemental Video 1

Supplemental Video 2

Supplemental Video 3

Supplemental Video 4

Supplemental Video 5

Supplemental Video 6

Supplemental Video 7

Supplemental Video 8

Supplemental Video 9

Supplemental Video 10

Supplemental Video 11

Supplemental Video 12

Supplemental Video 13

Supplemental Video 14

Supplemental Video 15

## Data Availability

The data that support the findings of this study are available from the corresponding author upon reasonable request.
