# Supplementary material for: Surface‐Sensitive Fractioning of Flowing Colloidal Suspensions Sedimented at a Photochemically Active Wall
Source: Small. 2025 May 12;21(24):2500012. doi: 10.1002/smll.202500012 (PMC12177868; doi:10.1002/smll.202500012)
Supplement: Supplementary file 1 — Supporting Information [file SMLL-21-2500012-s008.pdf]

NANO · MICRO small

# Supporting Information

for *Small*, DOI 10.1002/smll.202500012

Surface-Sensitive Fractioning of Flowing Colloidal Suspensions Sedimented at a Photochemically Active Wall

*Daniela Vasquez-Muñoz, Mihail Nicola Popescu, Anjali Sharma, Fabian Rohne, Isabel Meier, Phillip Ortner, Sarah Loebner, James Robert Benson, Nino Lomadze, Stephan Eickelmann, Svetlana Santer and Marek Bekir**

# Supporting Information

## Surface-sensitive fractioning of flowing colloidal suspensions sedimented at a photochemically active wall

*Daniela Vasquez-Muñoz, Mihail N. Popescu, Anjali Sharma, Fabian Rohne, Isabel Meier, Phillip Ortner, Sarah Loebner, James R. Benson, Nino Lomadze, Stephan Eickelmann, Svetlana Santer, Marek Bekir**

### S1 Characterization of the PVPBMA polymer coating film

### S1.1 Preparation and characterization of the polymer (PVPBMA) coating film

The viscous poly (4-vinylpyridine-co-butyl methacrylate) (PVPBMA, **Figure S1a**) solution (1 g PVPBMA in 10 mL methanol) was deposited on a glass slide and subsequently spin coated to yield a macroscopic, uniform, transparent PVPBMA layer with thickness of approximately 0.1 mm. The polymer layer thus obtained adheres strongly to the glass. It exhibits a rather rough interface and a bulk structure containing randomly distributed micropores (**Figure S1f** and **j**) of depths up to 0.4 µm (**Figure S1c,d**) and nanopores with depths of up to 20 nm (0.02 µm) (**Figure 1g,h**), as measured utilizing Atomic Force Microscopy (AFM) (see **Figure S1**, and the **Video S15**). Owing to this very porous structure, the PVPBMA layer can adsorb large amounts of surfactant molecules when put in contact with the aqueous solution of surfactant.

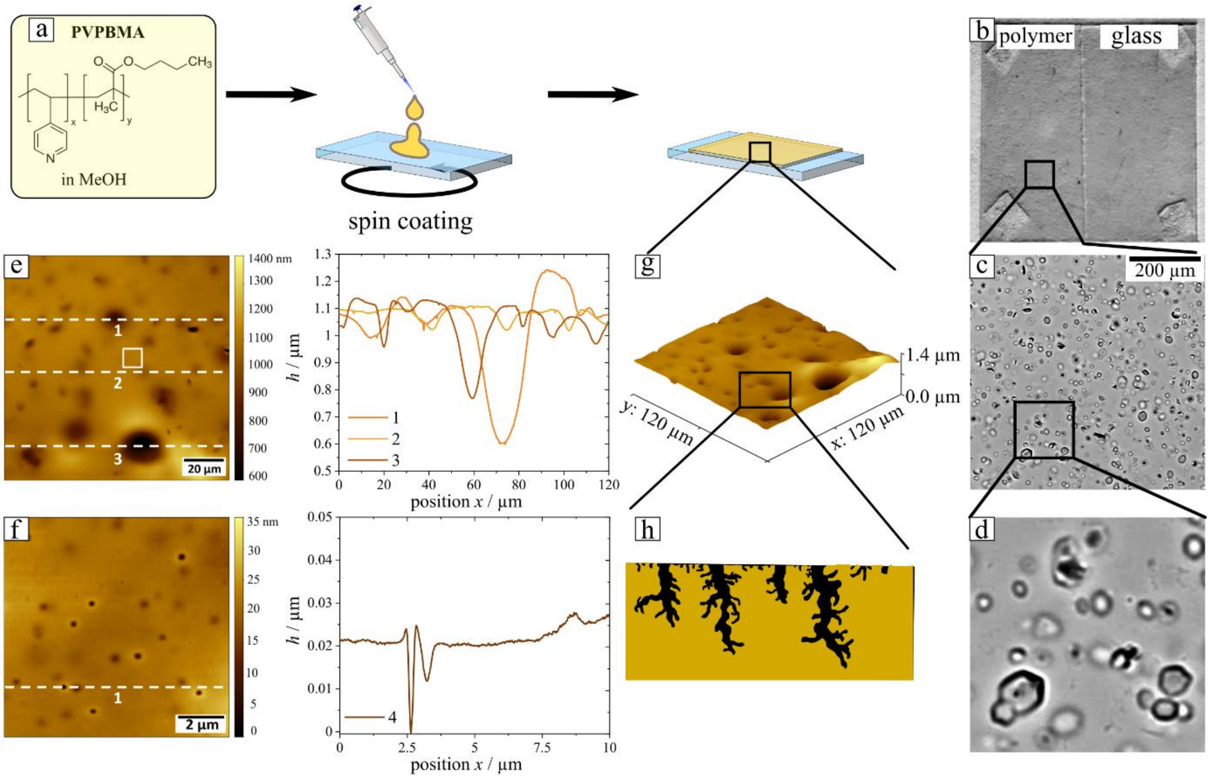


**Figure S1**. (a) Chemical structure of the PVPBMA polymer and a scheme of the polymer-film preparation. (b) Optical micrograph of borosilicate glass (right) and the polymer coated glass (left) surfaces. (c, d) Zoomed-in optical microscopy images of the polymer surface marked by the black rectangle in (b). (e) Contact mode AFM image of a scan of the polymer surface (left) and the corresponding cross-section along the three lines (1, 2, 3) marked in the scan (right). (f) Similar as (e) but for a smaller area (see the scale bar). (g) 3D AFM image of the polymer surface and (h) a heuristic depiction of a possible geometry of the pores within the film.

## S1.2 Supporting experimental data for absorbance and desorption via QCM-D studies

***Summary of the studies on the adsorption and desorption on PVPBMA interface***

By the use of a photo-sensitive surfactant (**Figure S2a**) this interface strongly adsorbs the more hydrophobic *trans*-isomer; while under UV light irradiation it effectively converts all *trans* to the more hydrophilic *cis* isomer, which gives rise to an initial burst and continuing release over several hours of the *cis*-isomer into the bulk solution (**Figure S2b** and **c**). This observation is demonstrated *via* quartz crystal microbalance with dissipation (QCM-D) measurements; they are displayed in **Figure S2d–g** for a PVPBMA coated borosilicate crystal, and they are compared with those corresponding to when a plain borosilicate interface is used. Data in **Figure S2d**,**f** exhibits fast saturation of glass interface at 1 mM surfactant concentration with a final molecular density of 4 molecules per $nm^2$, while for PVPBMA after a critical covering of 5 molecules per $nm^2$ there is a slower continuing adsorption, which does not approach saturation in the recorded time frame of 8000 s, up to a density of 140 molecules per $nm^2$. The *trans*-isomer adsorption kinetics on PVPBMA is best described by a double- exponential fitting (**Figure S3)**. Data indicate a fast multilayer formation (I) (**Figure S2b, d**) at 1mM surfactant concentration on the surface similar to the one on glass (5 molecules/$nm^2$),[1] and the continuing slower adsorption most likely results from the lower diffusivity of the molecules inside the polymeric matrix of the porous PVPBMA interfaces/layer (II). Further, the strong adsorbing *trans*-isomer tendency is also supported by the results of UV-Vis experiments (see details in **Supporting Information Section S1.2.4**). The entire amount of adsorbed *trans*-isomers is released under UV-irradiation ($\lambda$ = 365 nm, $I$ = 20 mW $cm^{-2}$) over a time of ~ 3 h. The releasing rate data is fitted by a tri-exponential mass desorption relation (**Figure S4**) indicating a *cis* isomer burst at the outer layer (III) (**Figure S2c**, **e**), presumably due to a high diffusivity of the molecules in water, followed by a slower rate from lower diffusion of the molecules inside the mesoporous structure of PVPBMA as well as slow diffusion rate though the polymer matrix (IV) (see details in **Supporting Information**, **Section S1.2.1**).

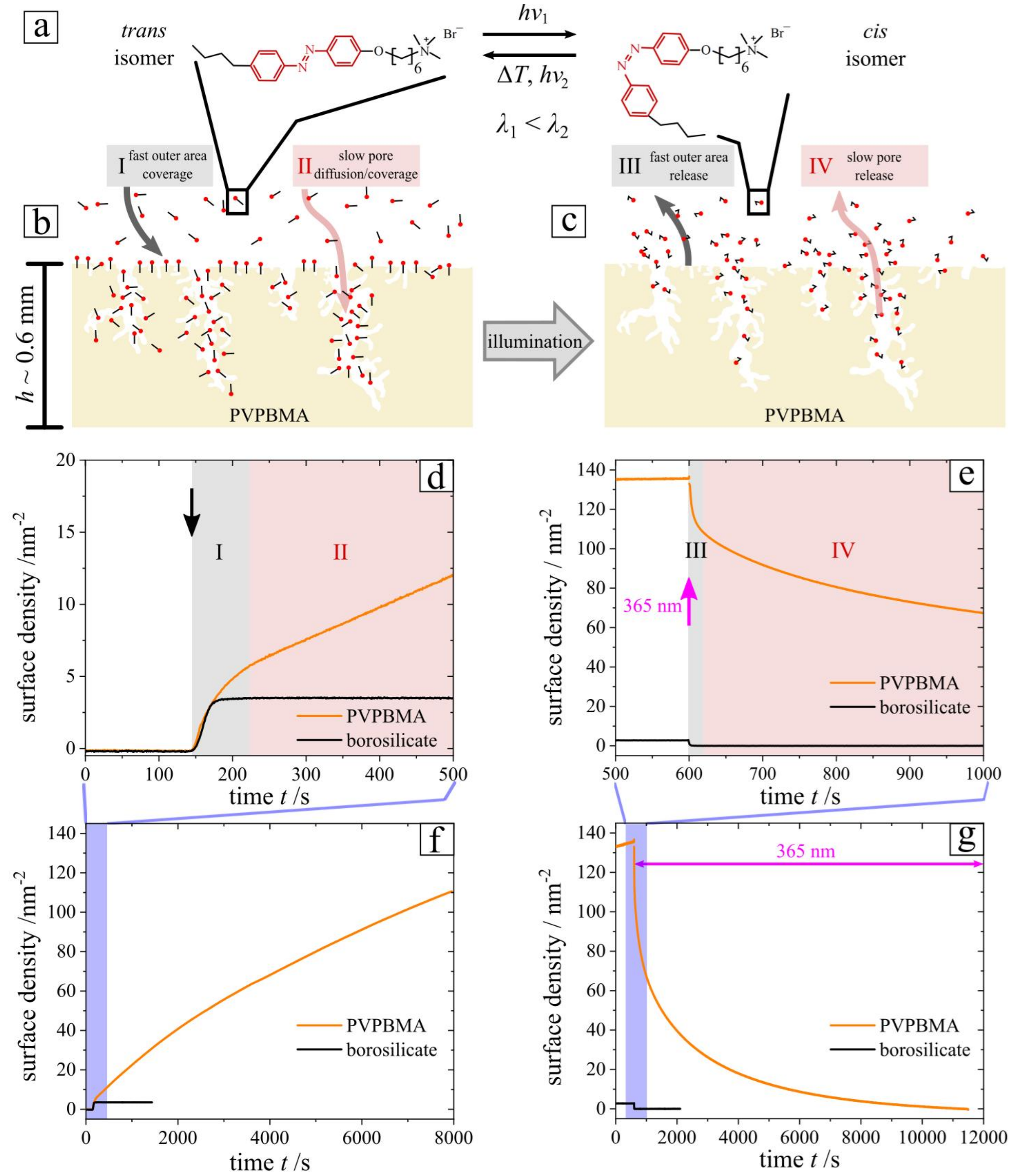


**Figure S2**. (a) Chemical structure of azobenzene containing surfactant and their photoisomerization states. (b) Cartoon of the hypothesized scenarios of surfactant adsorption on the polymer surface without illumination and (c) desorption during illumination. (d to g) Change of the surface density of surfactant at the two interfaces of interest as a function of irradiation and time. The surface density is calculated using QCM results as described in the text. (d, f) During adsorption the coverage of the surfaces with surfactant increases (region I) for both polymer surface (yellow curve) and glass (black curve). On the glass surface the number of adsorbed molecules saturates after ca. 30 seconds, while for the polymer it increases further (region II). (e, g) Desorption of the surfactant during irradiation with UV light. In the case of the glass surface, the complete desorption is achieved within 20 seconds (region III), while continuous release of the molecules out of the polymer surface takes place over more than 3 hours.

### S1.2.1 Estimation of the adsorption kinetics

The adsorbed and desorbed mass is calculated via the quartz crystal microbalance with dissipation, where frequency and dissipation shifts are recorded for the 5$^{th}$ overtone number.

We assume the PVPBMA layer as a big porous network of hydrophobic, multilayered and entangled individual polymer chains at nanometer scale, where the more hydrophobic *trans*-isomer may penetrate the interior of the polymer (similarly to the phenomenology concerning physiosorbed polymers and end-grafted polymer brushes).[2]

On a microscopic scale, the PVPBMA layer may be approximated as a diffuse interface, where the adsorption kinetics in comparison to the one at “hard” interfaces is different due to the difference of diffusion of isomers in the PVPBMA interior. For “hard” interfaces the rate of adsorption is proportional to the adsorption rate constant $k$ and the surface coverage $\theta$, $k\cdot\theta$, where the integrated rate law of the surface coverage predicts a mono-exponential relation for hard interfaces. The same is also valid for the desorption. Although the rate law of adsorption and desorption for a diffuse layer is similar, there are up to three different adsorption and desorption rates, expressed by three different values of $k$ that originate from three different diffusivities of the isomers:

1. A fast process at the outer interface covering or release from fast diffusion of isomer from or towards the bulk solution. (fast decay time)
2. A slower process at the inner interface of microsized pores covering and release from reduced diffusion of isomer in the pore interior. (medium decay time)
3. A very slow adsorption and release process resulting from lowest diffusivity through the polymer network. (slow decay time)

In this way, the integrated rate law of the surface coverage predicts also an exponential relation, where the surface coverage ~ adsorbed mass, $\Delta m$, may be approximated by up to a triple-exponential relation with different decay times. The consistency of the hypothesis of the different adsorption rates with the experimental data in **Figure S3** and **Figure S4** was checked via fitting with a mono-exponential:

$$\Delta m(t) = A \cdot \exp\left(-\frac{t}{\tau}\right) + y_0, \qquad \text{S1}$$

a double-exponential:

$$\Delta m(t) = A \cdot \exp\left(-\frac{t}{\tau_1}\right) + B \cdot \exp\left(-\frac{t}{\tau_2}\right) + y_0, \qquad \text{S2}$$

and a triple-exponential:

$$\Delta m(t) = A \cdot \exp\left(-\frac{t}{\tau_1}\right) + B \cdot \exp\left(-\frac{t}{\tau_2}\right) + C \cdot \exp\left(-\frac{t}{\tau_3}\right) + y_0, \qquad \text{S3}$$

with $y_0$, time $t$, decay time $\tau$, with $\tau = 1/k$ and $A$, $B$, $C$ as individual scaling factor are fitting parameters.

For fitting the data, the software Origin version 2019b[3] is used.

*Data for adsorption*

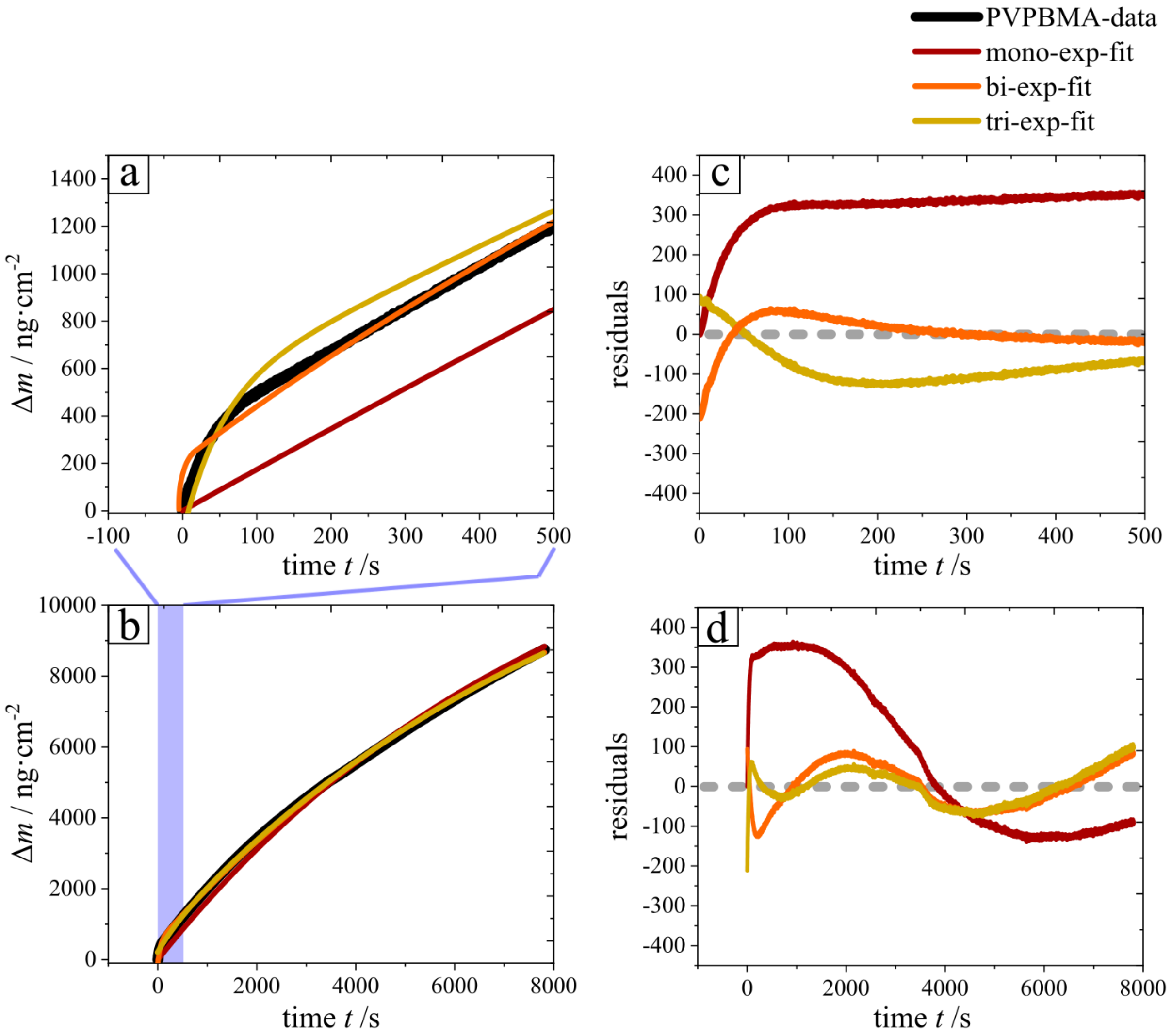


**Figure S3**. (a, b) Comparison of adsorption on PVPBMA experimental data with the fits. Data measured without photo-illumination. The blue rectangle illustrates the region shown in detail in the panel (b). (c,d) residuals.

**Table S1**. Fit values from data displayed in **Figure S3**.

| | mono | | bi | | tri | |
|---|---|---|---|---|---|---|
| | value | err | value | err | value | err |
| $y_0$ | 14419.40 | 0.00 | 14262.78 | 0.00 | 14419.40 | 0.00 |
| A | -14419.22 | 45.32 | -589.91 | 18.68 | -391.08 | 6.08 |
| $\tau_1$ | 8230.85 | 4.25 | 51.12 | 16.32 | 467.24 | 13.70 |
| B | — | — | -13766.60 | 17.50 | -6900.15 | 121.13 |
| $\tau_2$ | — | — | 8682.79 | 17.97 | 8948.57 | 18.56 |
| C | — | — | — | — | -6916.61 | 134.32 |
| $\tau_3$ | — | — | — | — | 8748.58 | 19.13 |

*Data for desorption*

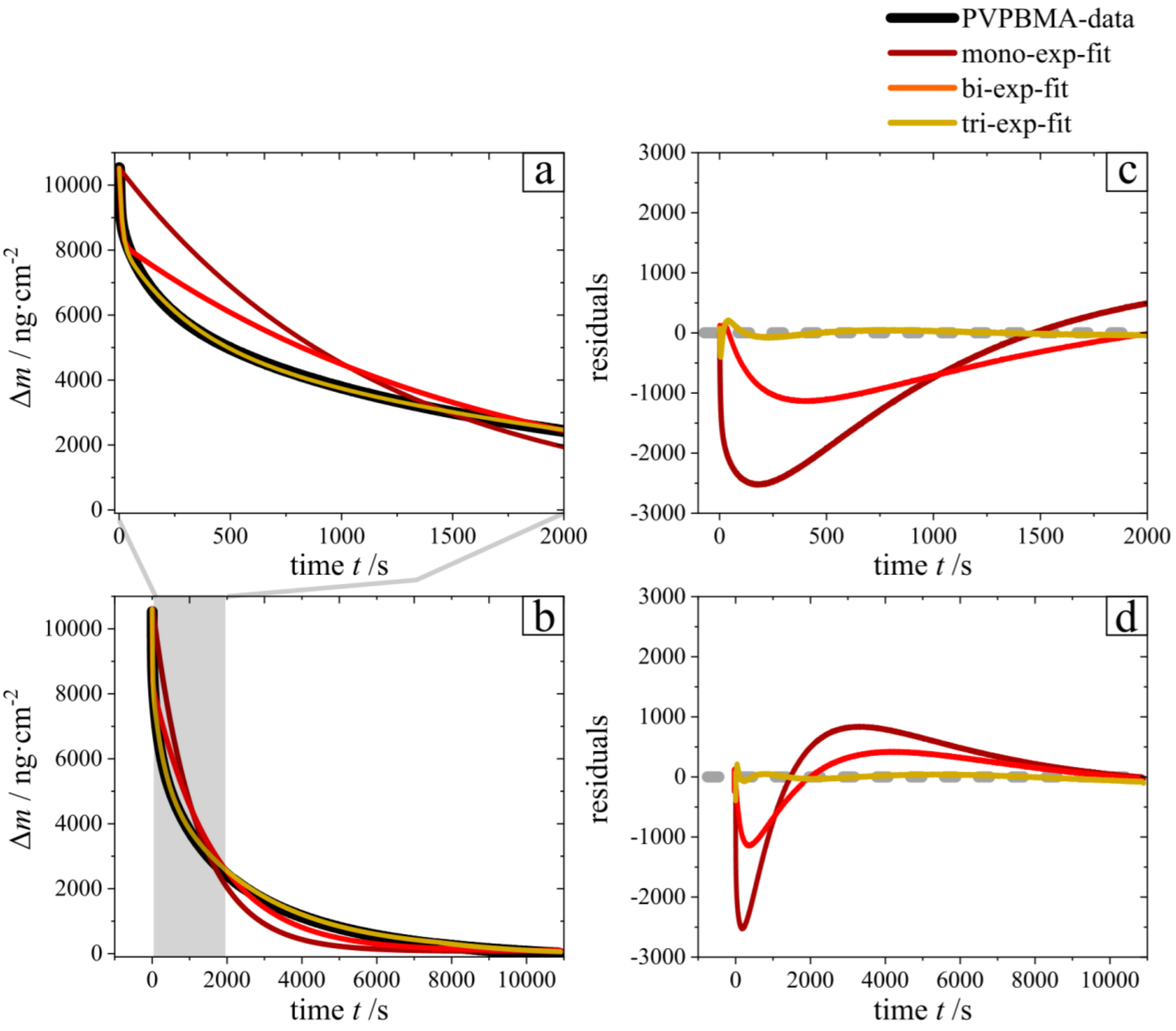


**Figure S4**. (a, b) Comparison of the experimental data for desorption from PVPBMA during photo-illumination with UV light ($\lambda$ = 365 nm) with the exponential fits. The gray rectangle in (b) illustrates the zoomed-in region shown in (a). (c, d) residuals.

**Table S2**. Fit values from data displayed in **Figure S4**.

| | mono | | bi | | tri | |
|---|---|---|---|---|---|---|
| | value | err | value | err | value | err |
| $y_0$ | 0.00 | 0.00 | 0.00 | 0.00 | 0.00 | 0.00 |
| A | 10527.00 | 117.20 | 2288.00 | 55.32 | 2288.30 | 55.32 |
| $\tau_1$ | 1181.97 | 1.90 | 9.52 | 0.41 | 12.82 | 0.05 |
| B | — | — | 8239.00 | 93.21 | 5379.82 | 89.43 |
| $\tau_2$ | — | — | 1645.94 | 1.51 | 2560.21 | 0.30 |
| C | — | — | — | — | 2862.34 | 18.26 |
| $\tau_3$ | — | — | — | — | 298.91 | 0.19 |

### S1.2.2 Additional PVPBMA interface analysis: Axial Porosity Distribution

To calculate the pore distribution as a function of position (measured from center of the radial spin coating) we record a 45 s video scan. Position change was done with a precise optical table step motor with movement velocity of 0.0225 cm/s. During the position change the interface of the PVPBMA was recorded via optical video microscopy measured in air (**Supporting Video S15**). For the image data analysis, the software Fiji is used. The images are transformed to a binary pixel map (via thresholding) with the default settings of the auto detection algorithm, resulting into a pixelated image of black regions (the holes) on a white background. The images are treated with fill hole algorithm (Fiji software) and the covered area % area is calculated as a function of frame number. By using the known framerate of 30 frames per seconded to convert the time to displacement, the % area is plotted as a function of center displacement displayed in **Figure S5**.

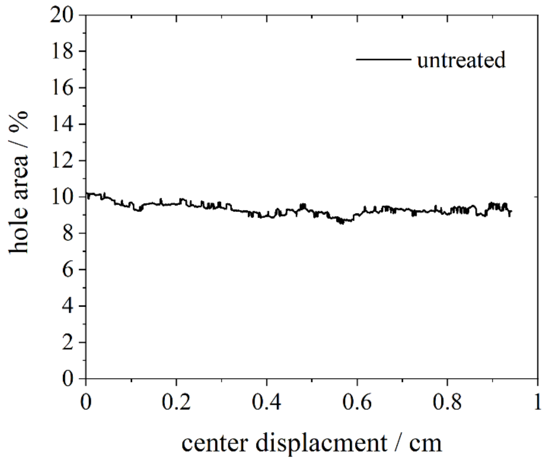


**Figure S5**. Calculated area occupied from porosity of PVPBMA. Data calculated from **Video S15**.

### S1.2.3 Additional PVPBMA interface analysis – Contact angle measurements

Principle measurements:

To measure the wettability of the PVPBMA polymer, we measured a series of contact angles on the PVPBMA interface with droplets of both MilliQ water (blue data points, **Figure S6a**) and MilliQ water with dissolved photosensitive surfactant in concentration of 1 mM (yellow data points, **Figure S6a**). We measured the contact angle on an "unloaded" the PVPBMA (plain PVPBMA), where the interface does not contain any adsorbed *trans*-surfactant isomers. Similarly, we repeated same measurements, where the PVPBMA interface prior have been immersed in an aqueous solution with dissolved photosensitive surfactants ($c$ = 1 mM) for 20 minutes and thus do contain adsorbed *trans*-surfactant isomers. The such "loaded" PVPBMA is labelled with blue marked area in rectangular shape displayed Figure S6a. We measured the contact angle for the "loaded PVPBMA" at three illuminates states: (1) no light illumination, which only contains *trans*-isomers in PVPBMA and in droplet; (2) with UV light ($\lambda$ = 365 nm), which contains mainly *cis* isomers (*trans*/*cis* ratio ~5/95) in PVPBMA and in droplet; as well as last (3) with green light ($\lambda$ = 520 nm), which contains mainly *trans* isomers (*trans*/*cis* ratio ~95/5) in PVPBMA and in droplet.

Note, each advancing contact angle (displayed as a hollow sign) has been measured 6 times, and the average is displayed as full sign with the minimal error shown in red brackets.

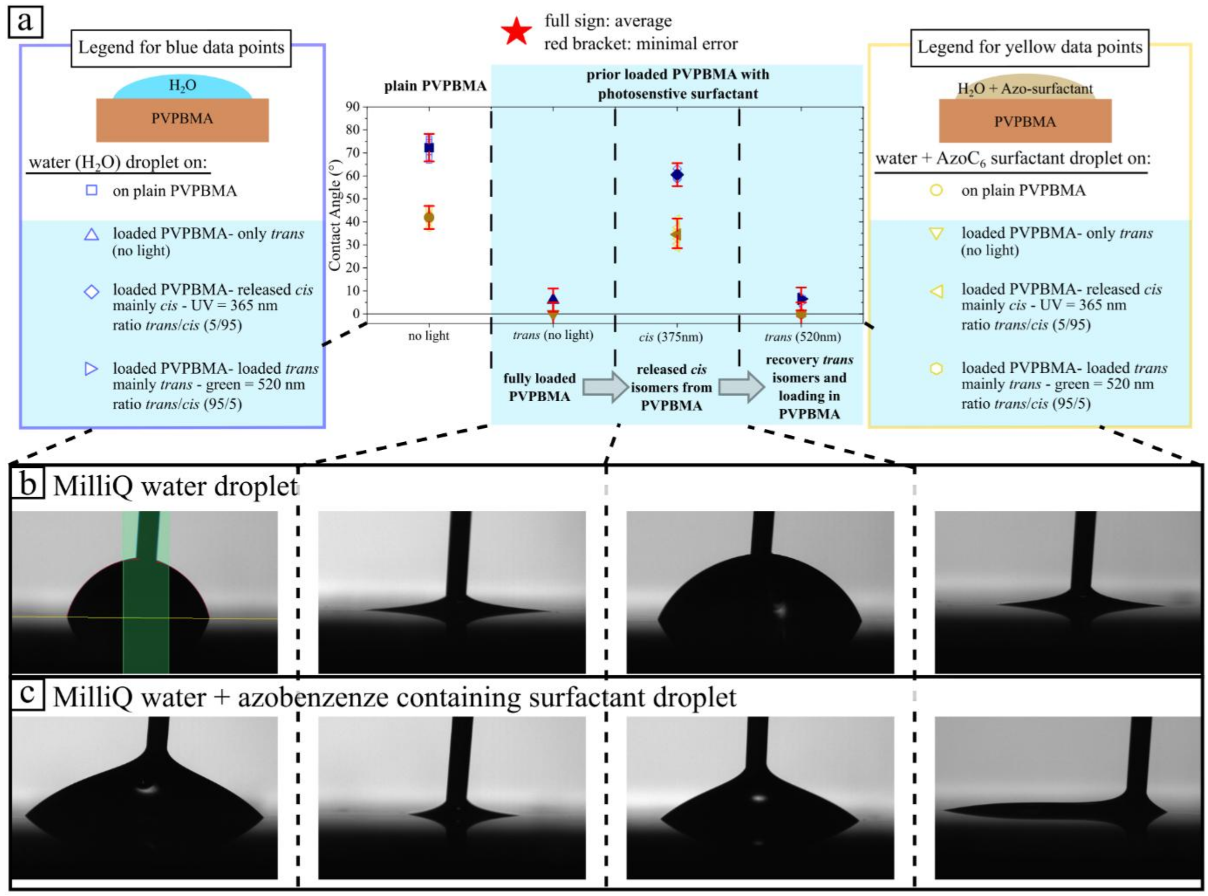


**Figure S6**. (a) Measured advancing contact angle for the plain (“unloaded”) and *trans* isomer (“loaded”) PVPBMA interface deposited on a typical microscope glass substrate. The (“loaded”) PVPBMA was exposed to different light illuminations. (b,c) example contact angle micrographs on PVPBMA with (b) MilliQ-water and (c) MilliQ-azobenzene surfactant solution ($c$ = 1 mM).

Data interpretation:

The PVPBMA is a hydrophobic polymer, which cannot be dissolved in water. This is also in good agreement with the contact angle measurements of both MilliQ water and surfactant solution ($c$ = 1 mM) on plain PVPVMA interface (**Figure S6a**). Data exhibits very hydrophobic interface confirmed from a measured contact angle of 72.4° for MilliQ and less for the surfactant solution ($\theta$ = 41.8°). In general, the contact angle of a droplet composed of MilliQ water is always bigger than for a droplet containing surfactant solution. This results from positive charging the PVPBMA due to adsorption the of positively charged *trans*-isomer surfactant on the interface, in good agreement with data shown in **Figure S2**.

When the PVPBMA is loaded with *trans*-isomers the original hydrophobic PVPBMA becomes very hydrophilic, proofed from a full wetting of droplets with measured contact angles close to zero ($\theta_{H2O}$ = 6.2°, $\theta_{surfactant\text{-}solution}$ = 0°). This supports the strong adhesion tendency of the surfactant in the PVPBMA and the resulting change in interface properties from strong hydrophobic into strong hydrophilic interface properties via surfactant loading. The surfactant can be released upon illumination with UV light ($\lambda$ = 365 nm dominant *cis*-isomer concentration), turning back the interface properties from hydrophilic into more hydrophobic ($\theta_{H2O}$ = 60.7°, $\theta_{surfactant\text{-}solution}$ = 34.6°). In contrast, when the “loaded” PVPBMA is exposed on

green illumination the isomers are dominantly in *trans*. Thus, the *trans*-isomers remain adsorbed and therefore the interface again is very hydrophilic $\theta_{H2O}$ = 6.3°, $\theta_{surfactant\text{-}solution}$ = 0°).

Experiments clearly show that the hydrophobic/hydrophilic character of the PVPBMA can be altered through the loading and releasing photosensitive surfactants from the polymer matrix interior. Especially the *cis*-isomer is more polar than the *trans* isomer, which prefers to solvate by water molecules rather than by in the hydrophobic matrix of the PVPBMA layer. This causes on illumination an expel *cis*-isomers into the water solution, and therefore on a macroscopic scale a change in the polarity of the PVPBMA. Thus, the PVPBMA (originally hydrophobic) become on *cis*-release hydrophobic again.

**S1.2.4 Additional PVPBMA interface analysis: characterization of the azobenzene containing surfactant storage via UV-Vis spectroscopy**

An additional measurement employing UV-Vis spectroscopy provides more support to the assumption that the *trans*-isomer is adsorbed within the PVPBMA porous structure.

A glass slide coated with PVPBMA is immersed into an aqueous solution of the azobenzene containing surfactant ($c$ = 0.5 mM; this particular concentration is chosen on purpose because the changes in the UV-Vis spectra in this case are clearly noticeable). Both the coated glass slide and the supernatant surfactant solution are measured before and after immersing the glass slide into the surfactant solution. After five hours, the glass slide is removed, gently washed with Millipore water, and then blow dried with $N_2$. Data corresponding to the dried PVPBMA before and after the exposure with surfactant solution is displayed in **Figure S7b**. The data corresponding to the surfactant solution before and after the exposure to the PVPBMA coated glass slide is displayed in **Figure S7c**. The data clearly demonstrates that the surfactant is strongly adsorbed from the PVPBMA interface, in good agreement with the findings in **Section 1.2.1**.

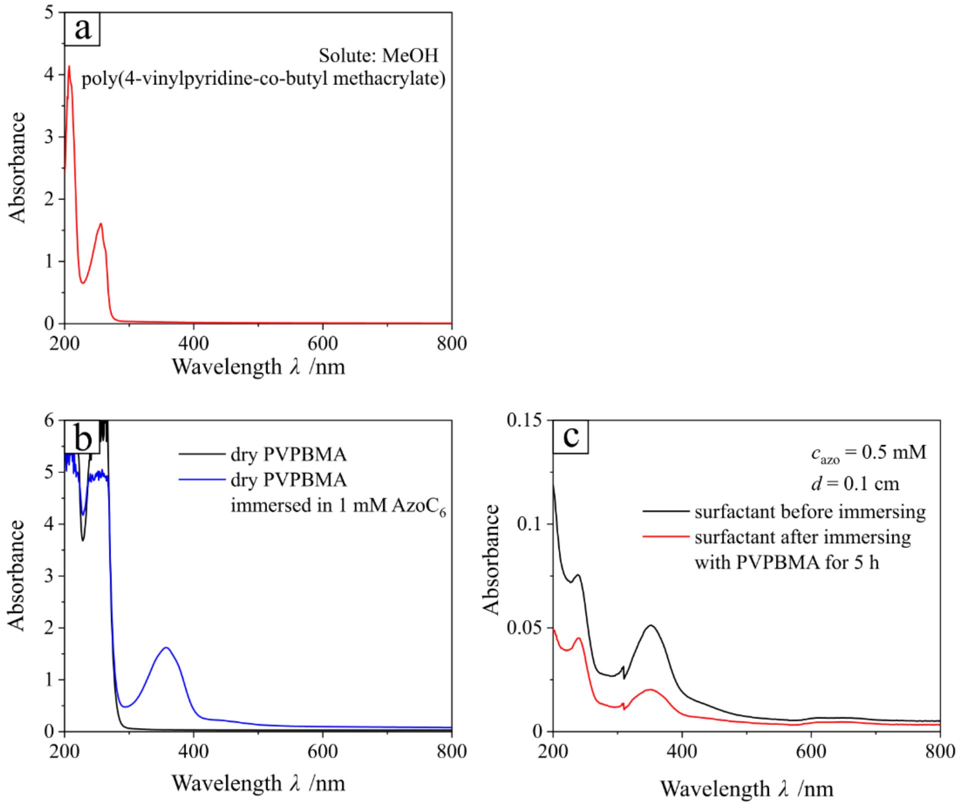


**Figure S7**. Absorption spectra of (a) PVPBMA fully dissolved in methanol (c = 0.1 wt%) (b) PVPBMA absorbed on glass interface (microscope glass slide) before and after immersing in azobenzene containing surfactant solution ($c$ = 0.5 mM). (c) Supernatant aqueous solution of azobenzene surfactant before and after the polymer coated slide is submerged.

### S1.2.5 Adhesion durability of the PVPVMA interface

To validate the adhesion durability of the PVPBMA at the glass substrate, we measured from optical inspection the adhesion tendency as a function of the exposer time in water and water-surfactant solution (*c* = 1 mM). To do that, we prepared the PVPBMA-glass-substrate and stored them in air environment (reference), in Millipore water (second reference) and the surfactant solution until the snapshot recording. Then we removed the PVPBMA-glass substrate from the solution, gently dried with nitrogen blow drying and took the snapshot. Afterwards the interface is placed into the solution. The recorded images are displayed **in Figure S8** as a time series from the top of the PVPBMA. Even after 9 days in the surfactant solution the interfaces remain adsorbed in the center of the glass where in all experiments the microfluidic channel was glued. At the edges of the glass slide the PVPBMA slowly peels off beginning at the 5$^{th}$ day and continuously detaching with a slow momentum from the edge into the center of the glass slide with increasing time. Furthermore, a strong color change from transparent into deep yellow can be seen and supports a huge loading capacity of the active interface constantly increasing with increasing time.

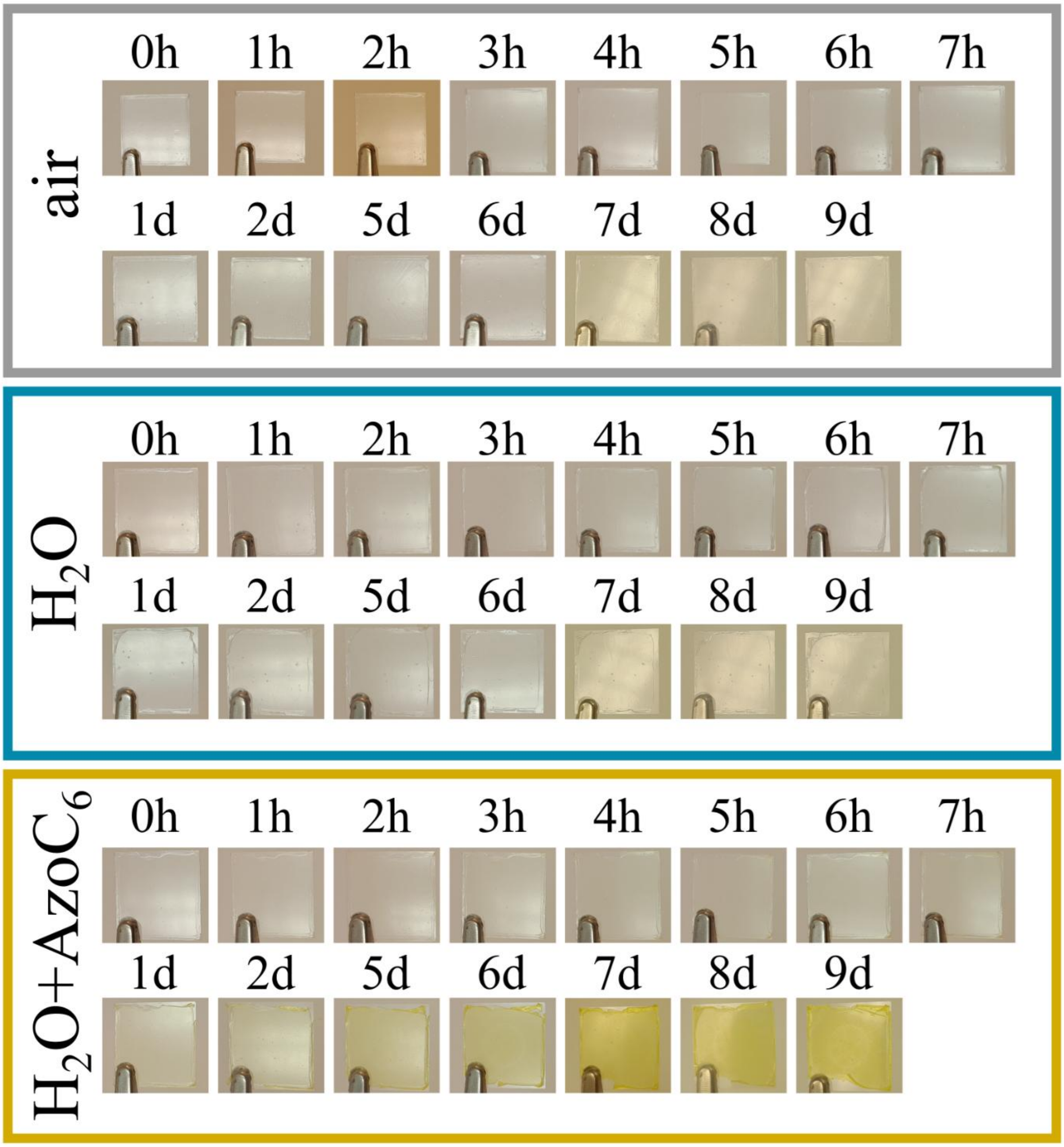


**Figure S8**. Snapshots as a function of the time for PVPBMA-glass-substrate immersed in (top) "air", (middle) Millipore water, (bottom) aqueous photosensitive surfactant solution ($c$ = 1 mM). A glass slide of 1 $cm^2$ (1 cm · 1 cm) is used.

**S2 Scanning Electron Microscopy (SEM) images typical compact and porous silica microparticles.**

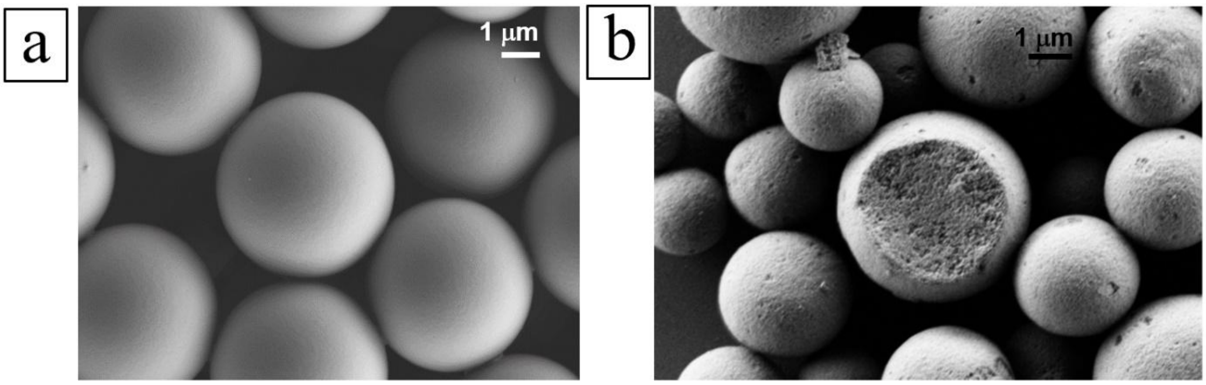


**Figure S9**. SEM micrograph of typical (a) compact (non-porous) $SiO_2$ and (b) porous $PSiO_2$ microparticles of diameter $D = 5$ $\mu$m.

**S3 Additional experimental data for particle velocity under microfluidic flow with and without illumination and for inert (glass) and chemically active (PVPBMA) bottom walls.**

**S3.1 Experimental data - Time-resolved average velocity dependency**

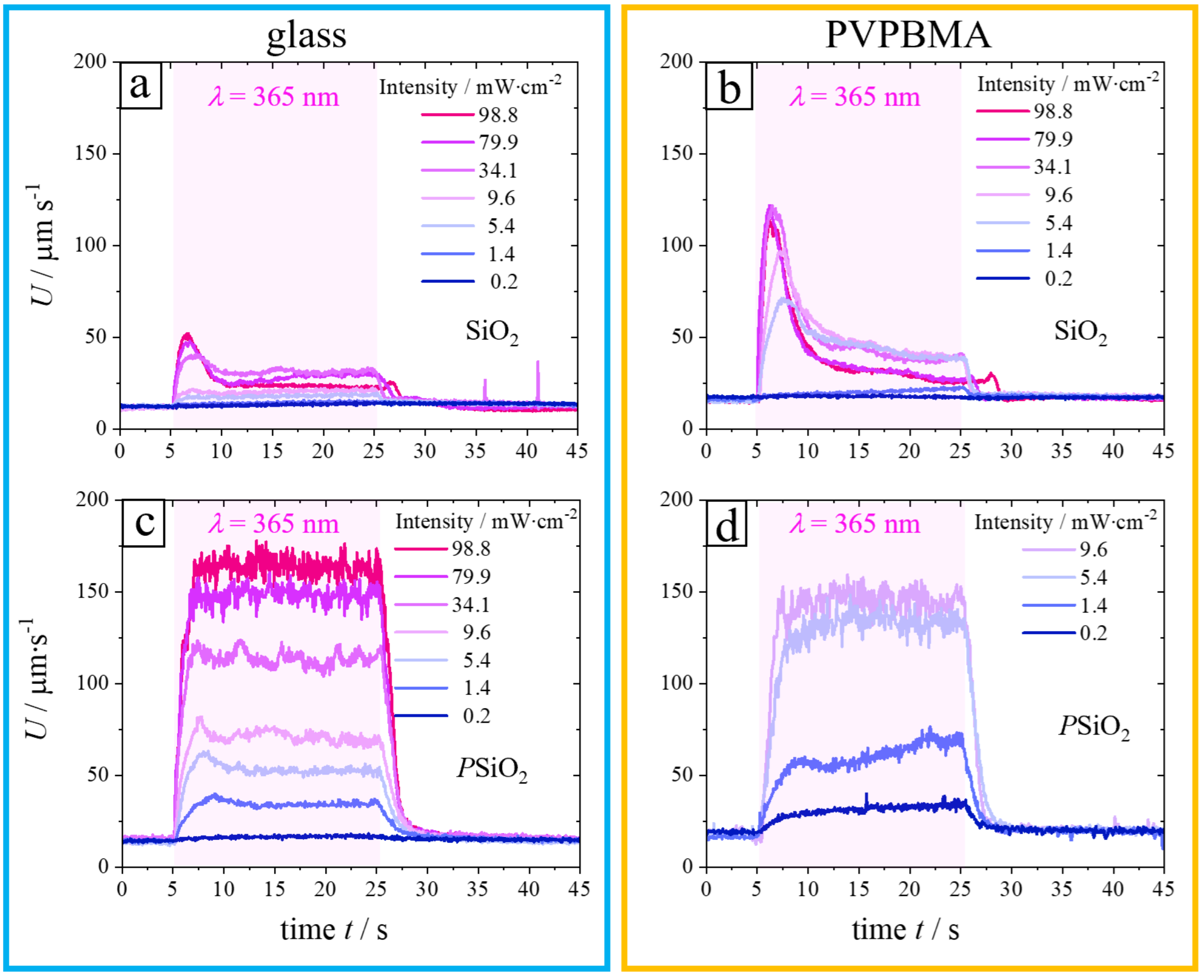


**Figure S10**. The effect of the active wall on the mean velocity for (a, b) compact ($SiO_2$) and (c, d) porous ($PSiO_2$) silica microparticles as a function of time under localized UV ($\lambda$ = 365 nm) illumination; the light is on during the time interval from 5s to 25s. (a, c) microparticles above a glass wall; (b, d) microparticles above a PVPBMA polymer coated wall. For data shown in **Figure 2** (**main article**) the average velocity is displayed from 20 to 25 s. Particle diameter is of compact particles is (4.0 ± 0.2) $\mu$m of porous particles is (3.0 ± 1.0) $\mu$m.

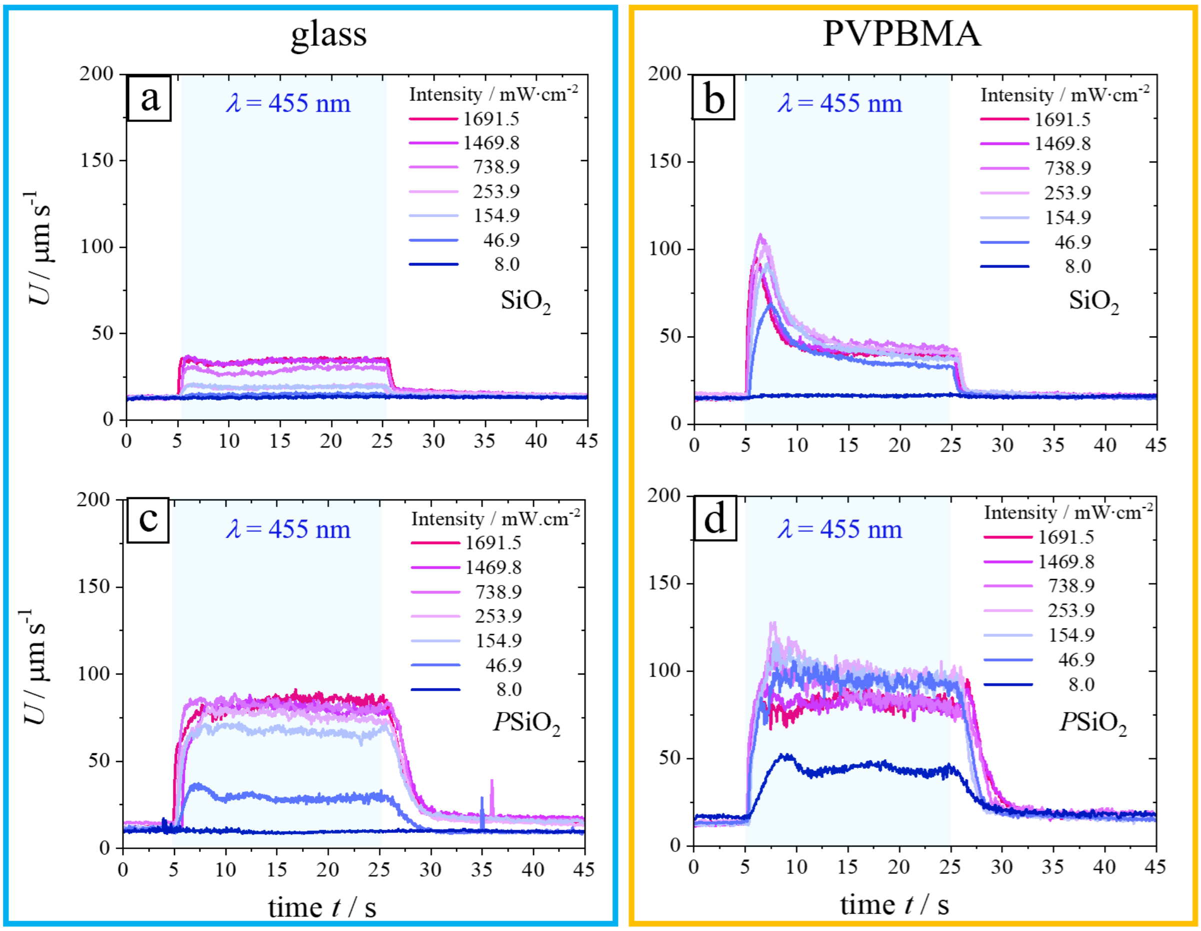


**Figure S11**. The effect of the active wall on the mean velocity for (a, b) compact silica particles ($SiO_2$) and (c, d) porous silica microparticles ($PSiO_2$) as a function of time under localized blue ($\lambda$ = 455 nm) illumination. (a, c) microparticles on glass surface; (b, d) microparticles on PVPBMA surface. For the data shown in **Figure 2** (**main article**) the average velocity is displayed from 20 to 25 s. Particle diameter is of compact particles is (4.0 ± 0.2) $\mu$m of porous particles is (3.0 ± 1.0) $\mu$m.

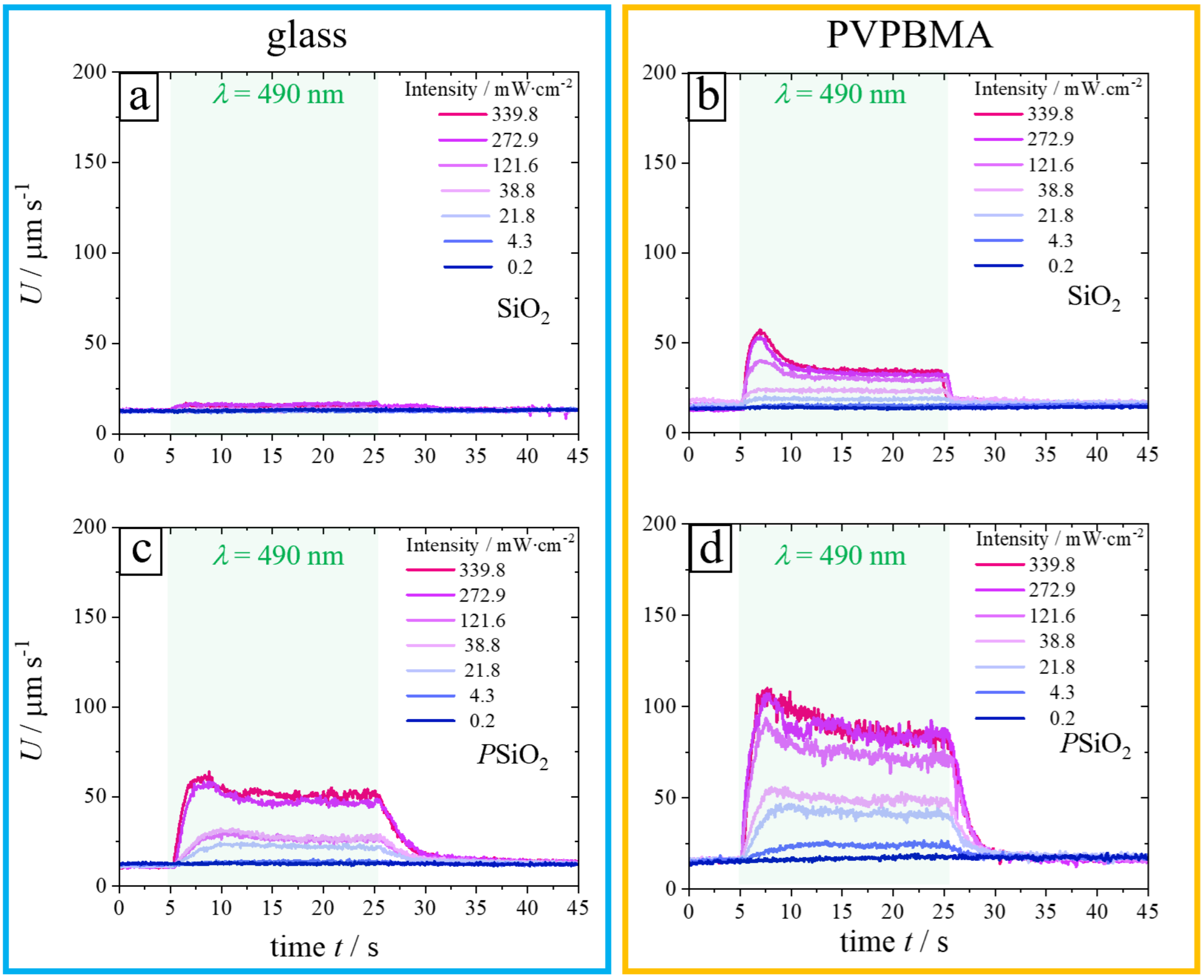


**Figure S12**. The effect of the active wall on the mean velocity for (a, b) compact silica particles ($SiO_2$) and (c, d) porous silica microparticles ($PSiO_2$) as a function of time under localized green ($\lambda$ = 490 nm) illumination. (a, c) microparticles on glass surface; (b, d) microparticles on PVPBMA surface. For the data shown in **Figure 2** (**main article**) the average velocity is displayed from 20 to25 s. Particle diameter is of compact particles is (4.0 ± 0.2) $\mu$m of porous particles is (3.0 ± 1.0) $\mu$m.

**S3.2 Experimental data – Comparison time-resolved average velocity dependency for different wavelengths**

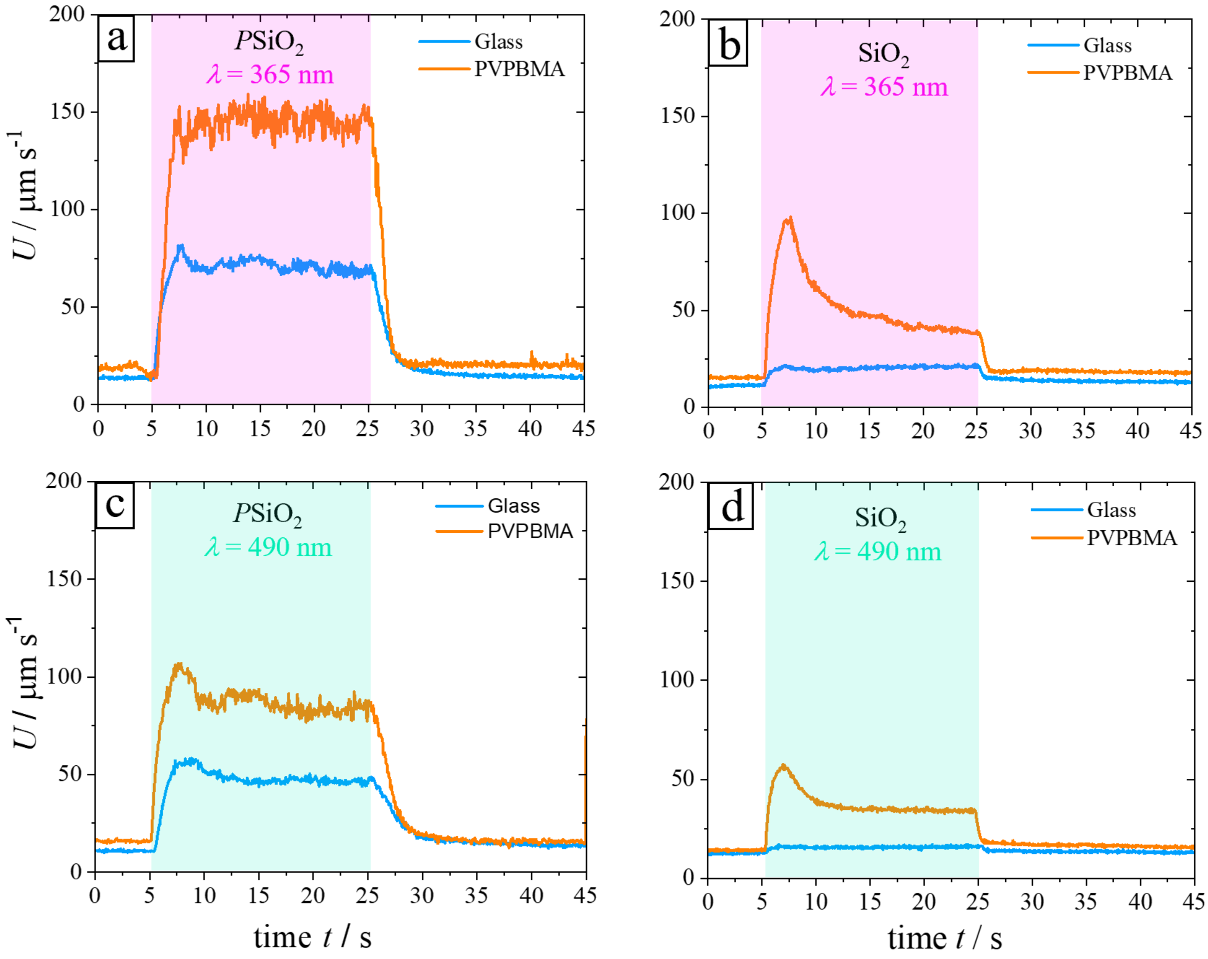


**Figure S13**. Mean velocity as a function of time of porous (a, c) and compact (b, d) $SiO_2$ on glass and polymer surfaces at two different light illumination: (a, b) $\lambda = 365$ nm ($I = 9.6$ mW·cm$^{-2}$) and (c, d) $\lambda = 490$ nm ($I = 40$ mW·cm$^{-2}$). Particle diameter is of compact particles is (4.0 ± 0.2) $\mu$m of porous particles is (3.0 ± 1.0) $\mu$m.

**S3.3 Experimental data – time resolved statistical distribution**

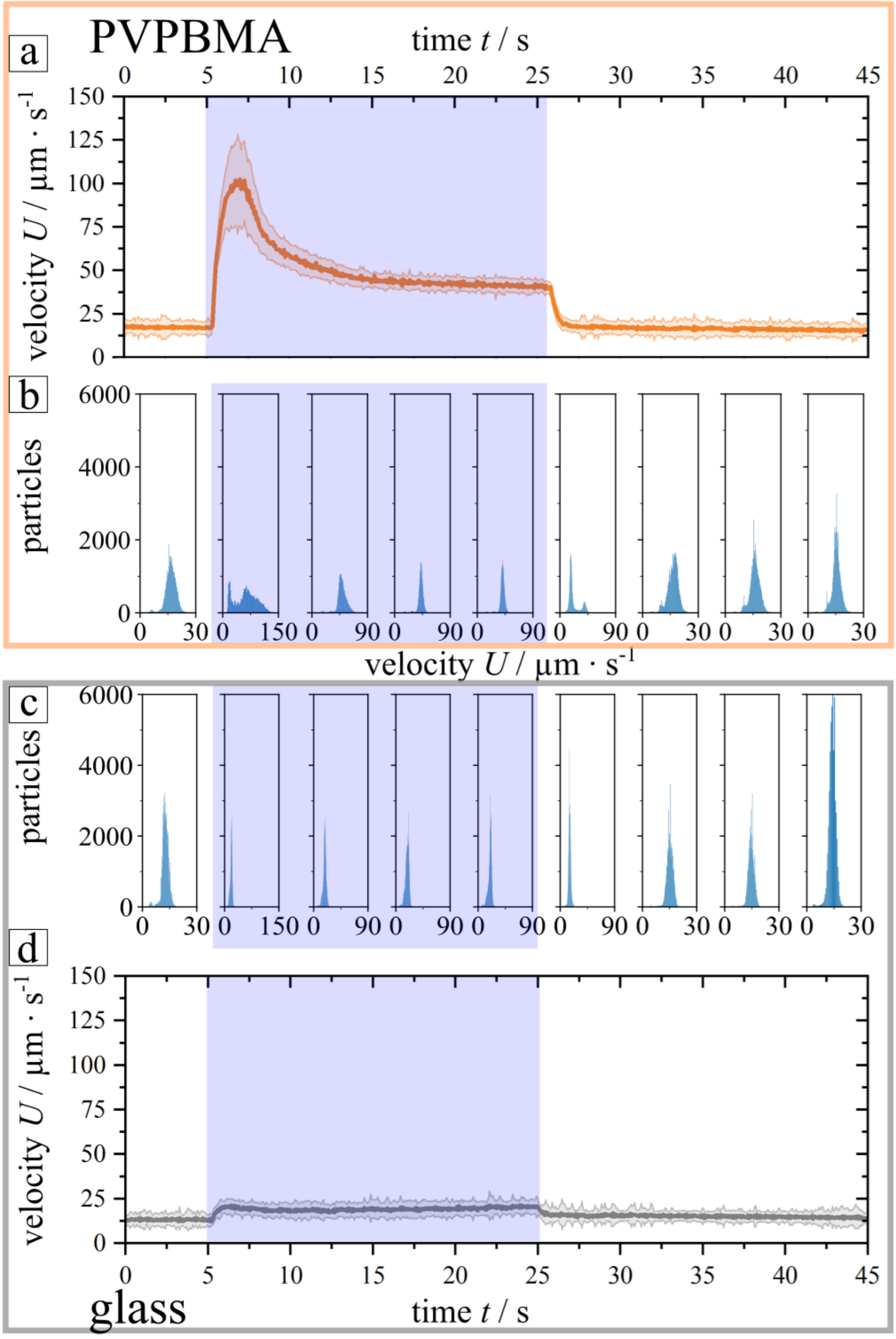


**Figure S14**. Visualization of the statistical distribution of compact particles ($SiO_2$) as a function of the time. Data displayed for compact particles ($SiO_2$) classified between active (PVPBMA) and inactive (glass) interface. (a, d) Mean drift velocity (thick line) and standard deviation (filled transparent area) as a function of time. (b,c) Corresponding velocity histograms intervals of 5 seconds. Data calculated from summing up tracks of particles over a 5 second time range. Data (a,b) is for the active interface (PVPBMA). Data (c,d) is for the inactive interface (glass). Data taken from **Figure 2c** (**main article**). The blue rectangles indicate the illumination range ($\lambda$ = 455 nm). Particle diameter is of compact particles is (4.0 ± 0.1) $\mu$m of porous particles is (3.0 ± 1.0) $\mu$m.

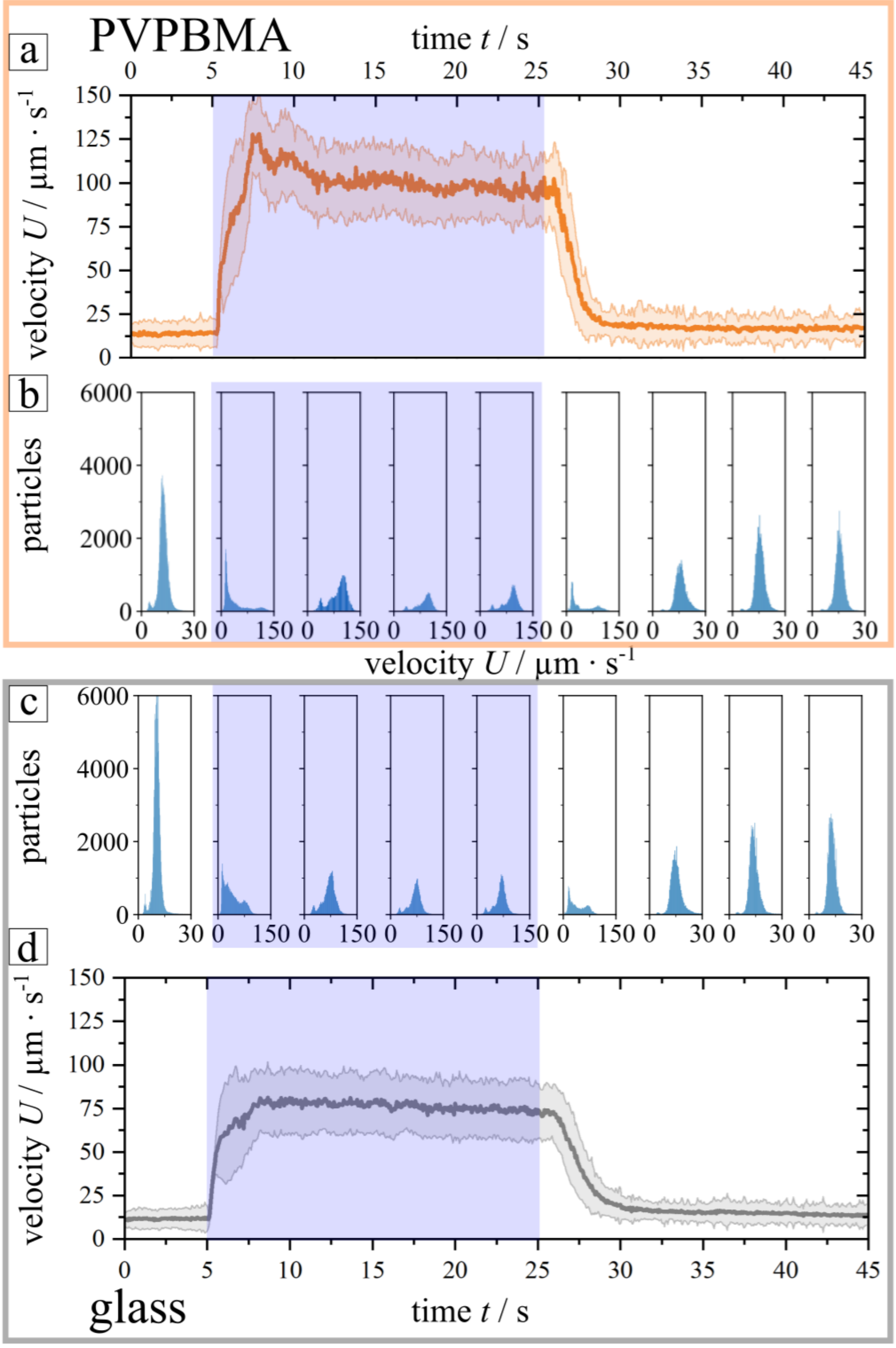


**Figure S15**. Visualization of the statistical distribution of particles as a function of the time. Data displayed for porous particles classified between active (PVPBMA) and inactive (glass) interface. (a, d) Mean drift velocity (thick line) and standard deviation (filled transparent area) as a function of time. (b,c) Corresponding velocity histograms intervals of 5 seconds. Data calculated from summing up tracks of particles over a 5 second time range. Data (a,b) is for the active interface (PVPBMA). Data (c,d) is for the inactive interface (glass). Data taken from **Figure 2d** (**main article**). The blue rectangles indicate the illumination range ($\lambda$ = 455 nm).

**S3.4 Experimental data – Voronoi tessellation**

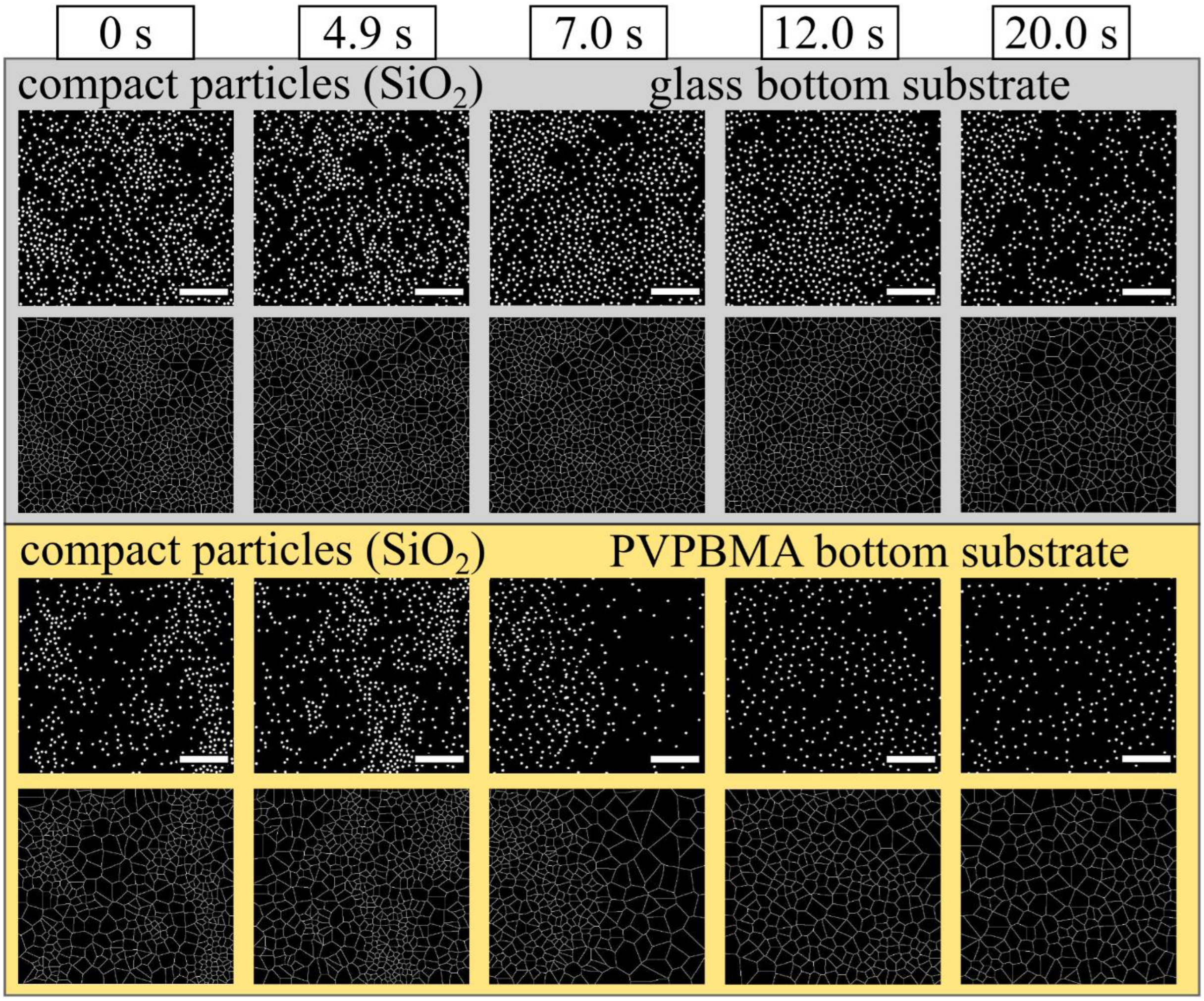


**Figure S16**. Measurements for compact particles ($SiO_2$). Snapshot series of optical micrographs (above) and corresponding Voronoi diagrams (below) for different times and classified by glass and PVPBMA interface. Scale bar (white stripe) is 50 µm. Data taken from **Video S5**. The time is displayed on top of the image series. Note, that the data correspond to the in original videos in the illuminated area on glass (Video S1) and PVPBMA (Video S2).

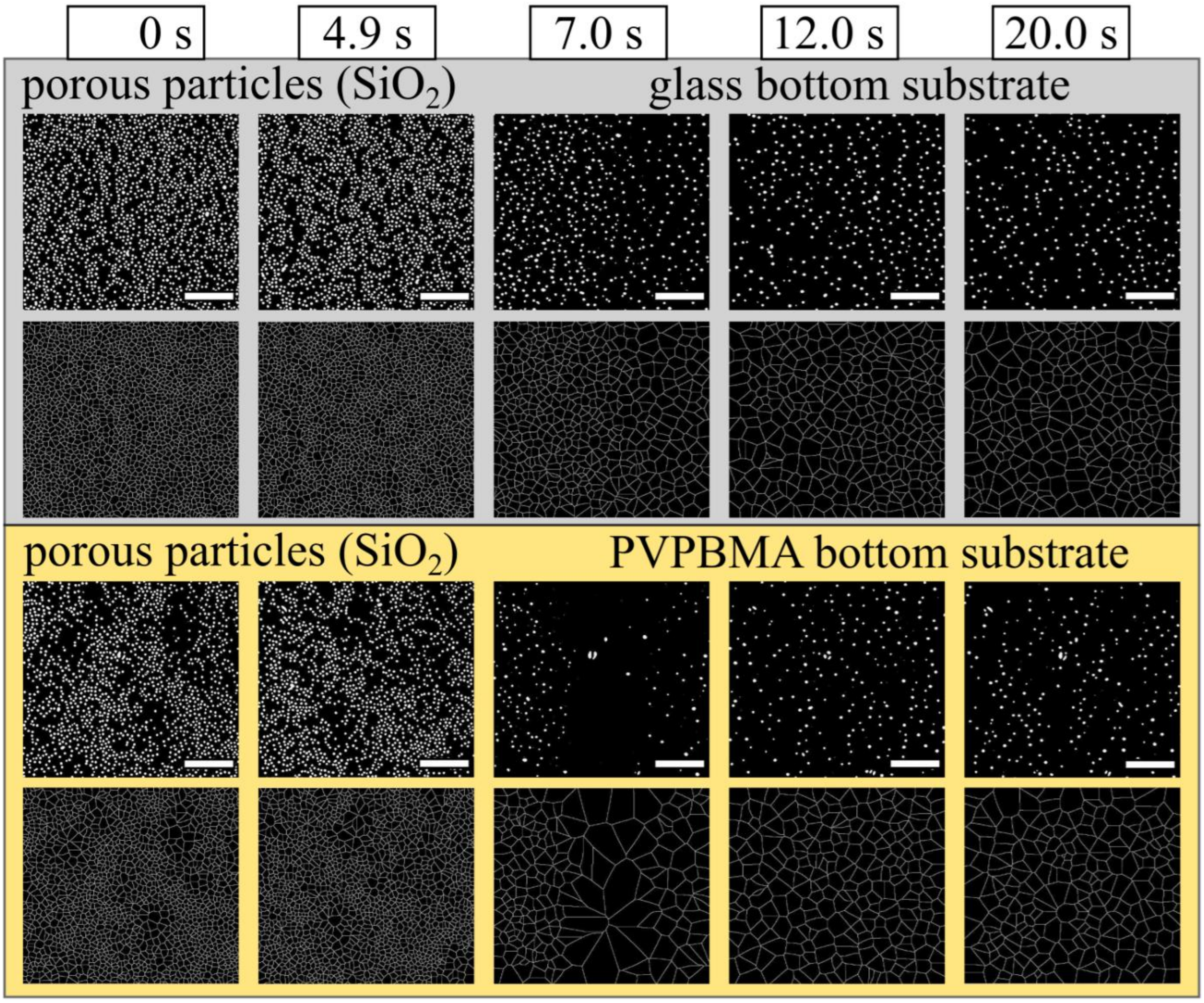


**Figure S17**. Measurements for porous particles ($PSiO_2$). Snapshot series of optical micrographs (above) and corresponding Voronoi diagrams (below) for different times and classified by glass and PVPBMA interface. Scale bar (white stripe) is 50 µm. Data taken from **Video S6**. The time is displayed on top of the image series. Note, that the data correspond to the in original videos in the illuminated area on glass (Video S3) and PVPBMA (Video S4).

## S3.5 Sample size for measured samples

**Table S3**. The average sample size per frame during light illumination. (a) The entries display, for each wavelength used, the adjusted intensity. (b) The entries (below headings glass and PVPBMA) represent the value of the sample size and the standard deviation (err). All sample sizes are summarized from the data shown in **Figure S10-S13** and **Figure 2** (main article).

| a | | | b | | | | | | | | | | | |
|---|---|---|---|---|---|---|---|---|---|---|---|---|---|---|
| for | for | for | glass | | | | | | PVPBMA | | | | | |
| **365 nm** | **455 nm** | **490 nm** | **plain silica ($SiO_2$), $D$ = 4 µm** | | | | | | | | | | | |
| ***I* [mW/cm²]** | ***I* [mW/cm²]** | ***I* [mW/cm²]** | **365 nm** | | **455 nm** | | **490 nm** | | **365 nm** | | **455 nm** | | **490 nm** | |
| | | | | err | | err | | err | | err | | err | | err |
| 100.0 | 1690.0 | 340.0 | 218.2 | 62.2 | 202.5 | 10.4 | 299.7 | 19.5 | 143.7 | 32.9 | 252.5 | 61.7 | 224.3 | 93.1 |
| 80.0 | 1470.0 | 273.0 | 192.8 | 46.5 | 218.2 | 10.2 | 336.0 | 20.1 | 136.2 | 39.6 | 183.1 | 51.0 | 309.0 | 75.6 |
| 34.1 | 740.0 | 120.0 | 214.0 | 77.2 | 209.4 | 10.0 | 416.4 | 24.3 | 115.4 | 29.2 | 188.7 | 88.4 | 305.3 | 59.3 |
| 9.6 | 253.0 | 40.0 | 295.4 | 62.8 | 317.1 | 17.0 | 385.0 | 25.7 | 192.6 | 65.5 | 163.0 | 60.6 | 292.9 | 39.3 |
| 5.4 | 154.0 | 21.0 | 231.2 | 48.4 | 439.3 | 7.6 | 372.9 | 24.8 | 203.9 | 63.3 | 248.4 | 98.6 | 368.1 | 44.1 |
| 1.4 | 47.0 | 4.0 | 220.7 | 65.1 | 384.9 | 21.5 | 370.1 | 24.5 | 297.3 | 138.7 | 309.8 | 75.6 | 481.6 | 62.5 |
| 0.2 | 8.0 | 0.2 | 265.1 | 62.5 | 451.3 | 20.0 | 264.3 | 19.8 | 268.3 | 127.0 | 548.5 | 18.0 | 601.9 | 15.3 |
| **365 nm** | **455 nm** | **490 nm** | **porous silica ($PSiO_2$), $D$ = 3 µm** | | | | | | | | | | | |
| ***I* [mW/cm²]** | ***I* [mW/cm²]** | ***I* [mW/cm²]** | **365 nm** | | **455 nm** | | **490 nm** | | **365 nm** | | **455 nm** | | **490** | |
| | | | | err | | err | | | | err | | err | | err |
| 100.0 | 1690.0 | 340.0 | 45.7 | 9.6 | 126.5 | 0.9 | 189.2 | 4.0 | 86.2 | 1.9 | 134.8 | 15.6 | 118.3 | 1.9 |
| 80.0 | 1470.0 | 273.0 | 53.2 | 9.2 | 152.8 | 2.6 | 182.0 | 4.2 | 43.8 | 1.4 | 158.8 | 17.0 | 119.8 | 2.6 |
| 34.1 | 740.0 | 120.0 | 64.4 | 10.8 | 157.4 | 1.8 | 310.4 | 8.7 | 55.8 | 1.3 | 122.6 | 22.6 | 133.4 | 2.8 |
| 9.6 | 253.0 | 40.0 | 120.5 | 24.2 | 167.1 | 2.2 | 304.4 | 7.6 | 55.5 | 1.3 | 135.5 | 17.4 | 189.1 | 4.5 |
| 5.4 | 154.0 | 21.0 | 151.7 | 35.4 | 155.1 | 3.3 | 356.0 | 11.2 | 60.8 | 1.3 | 122.7 | 22.6 | 208.6 | 5.6 |
| 1.4 | 47.0 | 4.0 | 243.0 | 81.9 | 303.6 | 16.0 | 534.1 | 15.0 | 104.3 | 2.7 | 110.2 | 17.7 | 355.9 | 8.7 |
| 0.2 | 8.0 | 0.2 | 390.2 | 143.5 | 524.2 | 10.6 | 525.2 | 17.4 | 245.1 | 7.0 | 231.6 | 32.0 | 486.5 | 11.8 |
| **365 nm** | **455 nm** | **490 nm** | **porous silica ($PSiO_2$), $D$ = 5 µm** | | | | | | | | | | | |
| ***I* [mW/cm²]** | ***I* [mW/cm²]** | ***I* [mW/cm²]** | **365 nm** | | **455 nm** | | **490 nm** | | **365 nm** | | **455 nm** | | **490 nm** | |
| | | | | err | | err | | err | | | | err | | err |
| 100.0 | 1690.0 | 340.0 | 48.9 | 8.2 | 74.6 | 9.8 | 90.5 | 16.1 | 32.0 | 2.0 | 74.2 | 8.0 | 64.5 | 11.7 |
| 80.0 | 1470.0 | 273.0 | 40.5 | 7.5 | 77.7 | 8.2 | 84.0 | 13.7 | 20.7 | 5.1 | 74.1 | 9.2 | 61.7 | 10.3 |
| 34.1 | 740.0 | 120.0 | 49.1 | 12.0 | 73.6 | 9.8 | 119.5 | 15.3 | 29.0 | 6.9 | 68.4 | 12.6 | 70.2 | 14.7 |
| 9.6 | 253.0 | 40.0 | 61.3 | 9.8 | 80.0 | 10.0 | 131.1 | 30.1 | 35.6 | 6.5 | 56.2 | 9.2 | 85.3 | 14.3 |
| 5.4 | 154.0 | 21.0 | 92.9 | 24.8 | 85.6 | 6.9 | 182.9 | 43.5 | 42.7 | 9.5 | 51.7 | 12.8 | 107.4 | 18.8 |
| 1.4 | 47.0 | 4.0 | 94.8 | 23.8 | 86.2 | 11.0 | 291.4 | 99.1 | 72.6 | 19.2 | 62.4 | 9.3 | 123.5 | 22.7 |
| 0.2 | 8.0 | 0.2 | 231.2 | 74.0 | 434.7 | 65.8 | 304.3 | 65.7 | 203.4 | 50.2 | 171.5 | 33.9 | 292.2 | 55.1 |
| **365 nm** | **455 nm** | **490 nm** | **porous silica ($PSiO_2$), $D$ = 20 µm** | | | | | | | | | | | |
| ***I* [mW/cm²]** | ***I* [mW/cm²]** | ***I* [mW/cm²]** | **365 nm** | | **455 nm** | | **490 nm** | | **365 nm** | | **455 nm** | | **490 nm** | |
| | | | | err | | err | | err | | | | err | | err |
| 100.0 | 1690.0 | 340.0 | – | – | 28.0 | 5.7 | 26.5 | 5.7 | – | – | 49.1 | 10.4 | 41.4 | 7.3 |
| 80.0 | 1470.0 | 273.0 | – | – | 16.9 | 6.1 | 29.9 | 5.2 | – | – | 45.4 | 10.0 | 73.5 | 13.0 |
| 34.1 | 740.0 | 120.0 | – | – | 35.0 | 6.6 | 36.2 | 8.4 | – | – | 58.3 | 11.6 | 77.4 | 12.8 |
| 9.6 | 253.0 | 40.0 | – | – | 47.7 | 10.0 | 41.3 | 8.6 | – | – | 52.3 | 8.6 | 71.1 | 14.3 |
| 5.4 | 154.0 | 21.0 | – | – | 40.3 | 5.9 | 50.4 | 9.8 | – | – | 53.8 | 9.4 | 31.7 | 6.2 |
| 1.4 | 47.0 | 4.0 | – | – | 40.3 | 5.9 | 44.5 | 8.5 | – | – | 53.8 | 11.8 | 36.9 | 11.2 |
| 0.2 | 8.0 | 0.2 | – | – | 44.7 | 9.3 | 49.1 | 7.5 | – | – | 53.5 | 7.0 | 76.9 | 19.0 |

**S3.6 Figure legend abbreviation for "weakly active" micro particles**

We define the particle type by two major factors.

1. The particle bulk material
2. The surface coating.

1. For the particle bulk material, we have two major substances used.
    *1.1.* **Silica** here abbreviated with **$SiO_2$**
    *1.2.* **Polystyrene** here abbreviated with **PS**

2. For surface coatings there are several coatings used. Below there is listed table.

**Table S4**. Fit values from data displayed in

| **Surface functionalization** | **Functional group's name** | **Chemical Structure** |
|---|---|---|
| Plain (for Polystyrene) | Partially sulfonated | 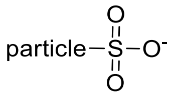 |
| Plain (for silica) | Hydroxyl | 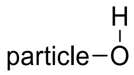 |
| $NH_2$ | Primary amine | 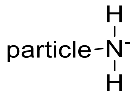 |
| $NR_3^+$ | Tertiary amine | 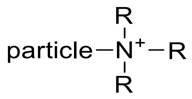 |
| COOH | Carboxylic acid | 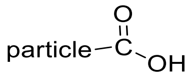 |
| $C_{18}$ | Octadecyl hydrocarbon | 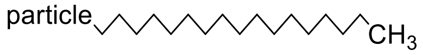 |

**S3.5 Additional experimental data for dispersions of differently surface modified particles flowing above a PVPBMA-coated wall and a glass wall, respectively**

<u>**Data for "from no separation" to "feasible separation"**</u>

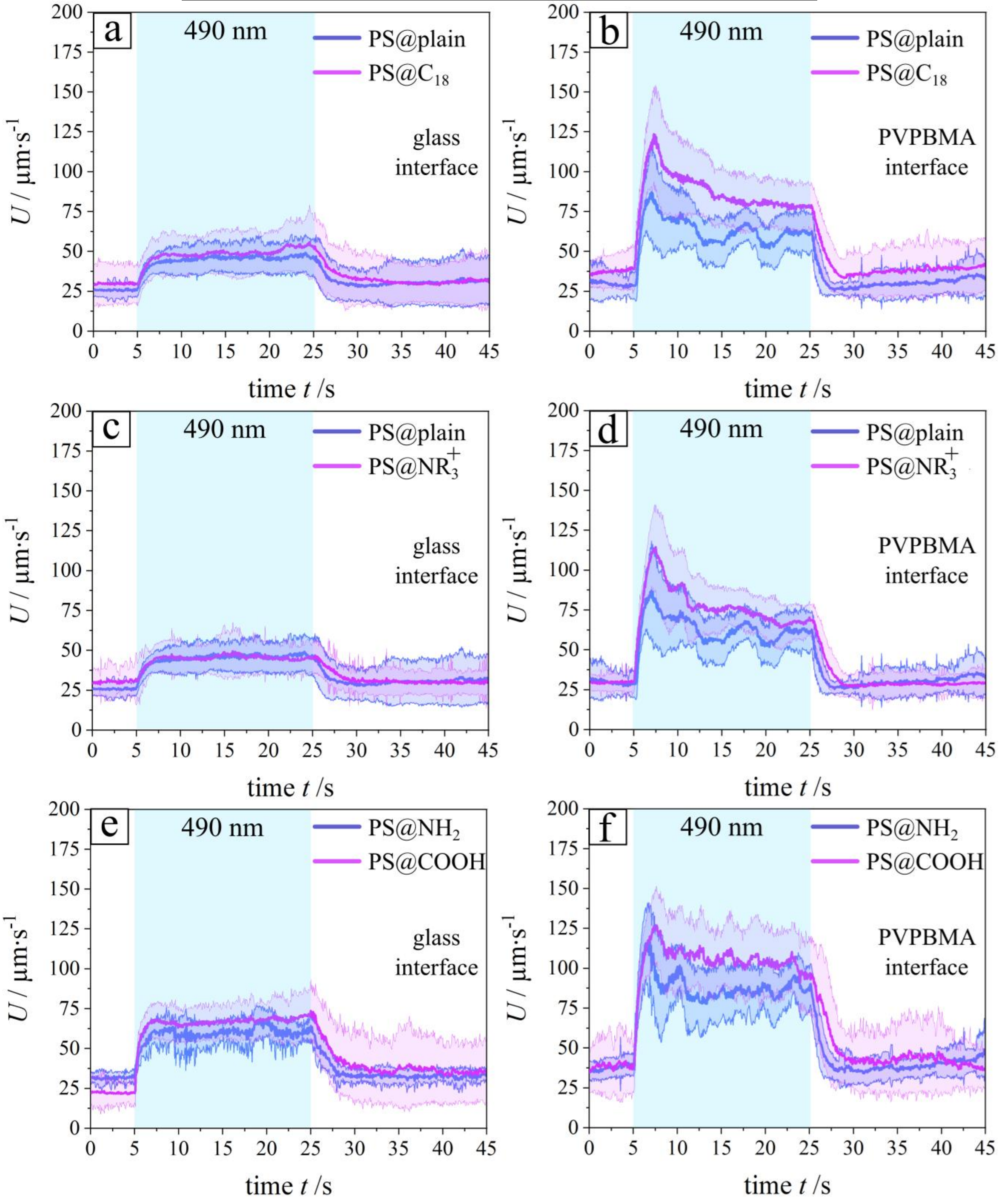


**Figure S18**. Mean velocity as a function of time. The measured experimental data corresponds to a glass wall (a,c,e) and a PVPBMA wall (b,d,f). The data shown corresponds to different particles pairs: for (a,b) PS@plain [glass, 73 ± 21], [PVPBMA, 76 ± 19] and PS@$C_{18}$ [glass, 89 ± 21], [PVPBMA, 76 ± 14], for (c,d) PS@plain [glass, 73 ± 21], [PVPBMA, 76 ± 19] and PS@$NR_3^+$ [glass, 83 ± 12], [PVPBMA, 87 ± 19], for (e,f) PS@$NH_2$ [glass, 93 ± 12], [PVPBMA, 98 ± 19] and PS@COOH [glass, 82 ± 21], [PVPBMA, 86 ± 26]. Please note that the average sample size per frame is displayed in the rectangular brackets. In all images the rectangular area illustrates time period of light illumination with $\lambda$ = 490 nm ($P$ = 11.5 mW·cm$^{-2}$). Particle diameter is (5 ± 0.1) $\mu$m.

**Data for "good separation" to "enhanced separation"**

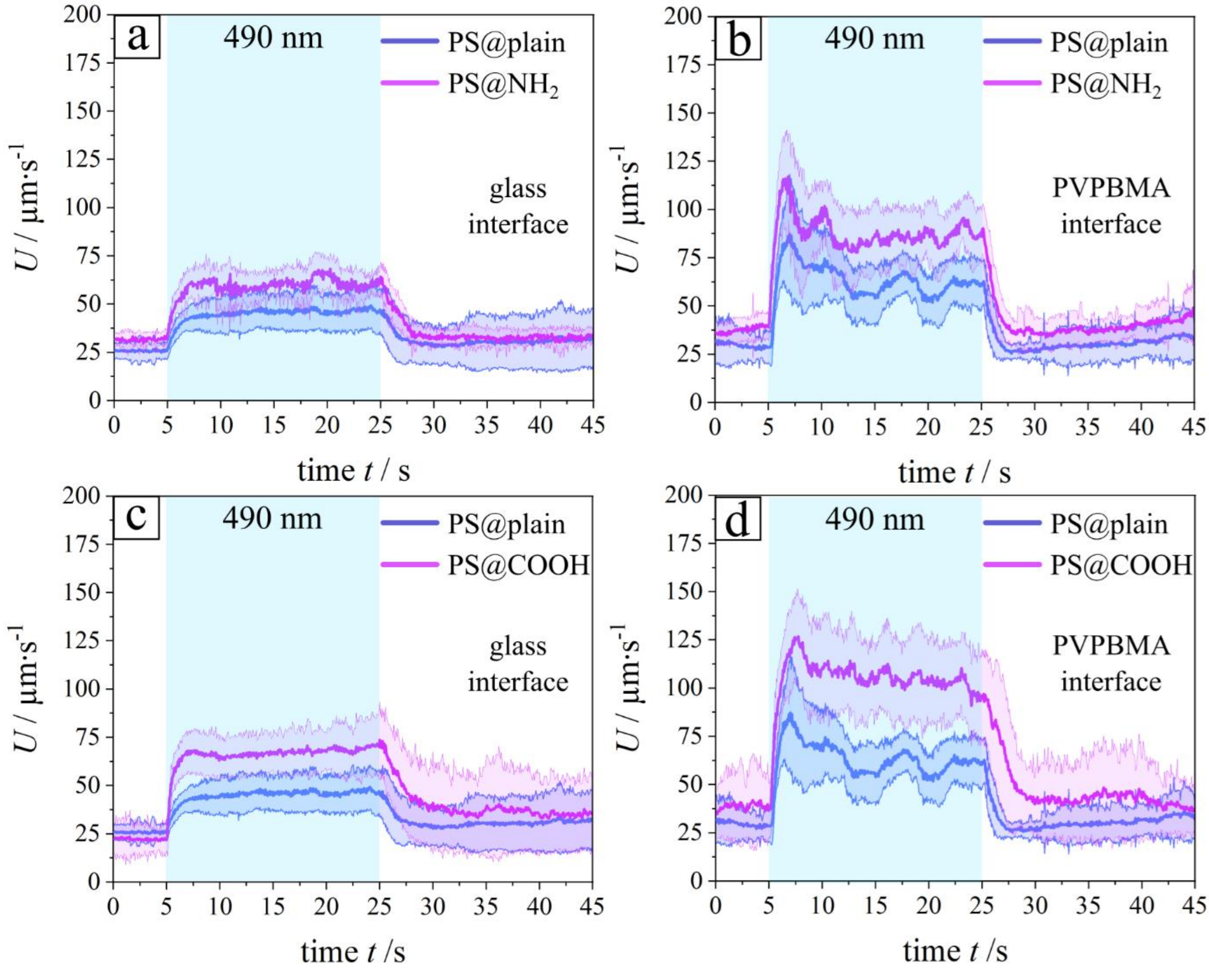


**Figure S19.** Mean velocity as a function of time. The measured experimental data corresponds to a glass wall (a,c) and a PVPBMA wall (b,d). The data shown corresponds to different particles pairs: for (a,b) PS@plain [glass, 73 ± 21], [PVPBMA, 76 ± 19] and PS@$NH_2$ [glass, 93 ± 12], [PVPBMA, 98 ± 19], for (c,d) PS@plain [glass, 73 ± 21], [PVPBMA, 76 ± 19] and PS@COOH [glass, 82 ± 21], [PVPBMA, 86 ± 26]. Please note that the average sample size per frame is displayed in the rectangular brackets. In all images the rectangular area illustrates time period of light illumination with $\lambda$ = 490 nm ($P$ = 11.5 mW·cm$^{-2}$). Particle diameter is (5 ± 0.1) $\mu$m.

**Data for "good separation" to "no improvement"**

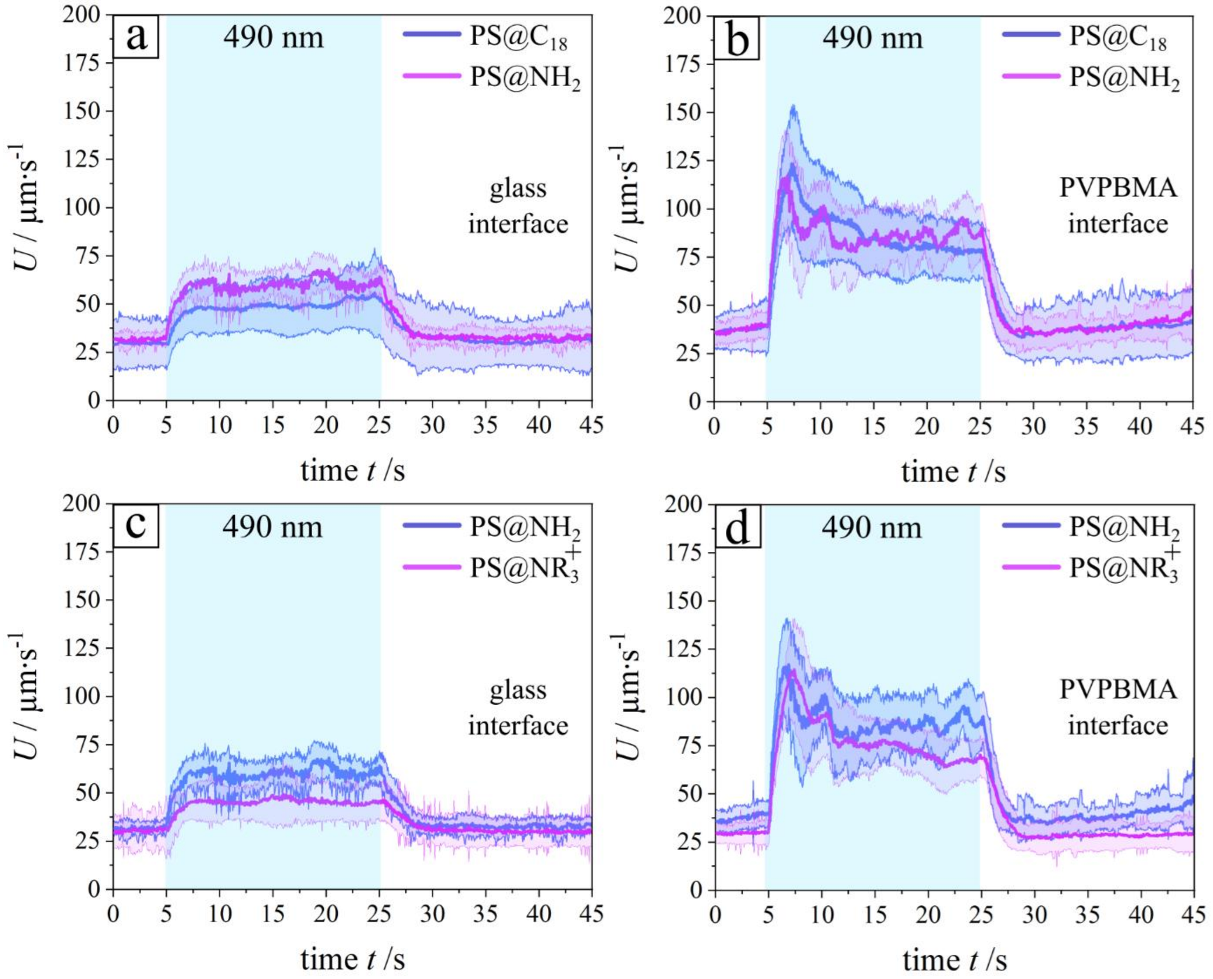


**Figure S20.** Mean velocity as a function of time. The measured experimental data corresponds to a glass wall (a,c) and a PVPBMA wall (b,d). The data shown corresponds to different particles pairs: for (a,b) PS@$NH_2$ [glass, 93 ± 12], [PVPBMA, 98 ± 19] and PS@$C_{18}$ [glass, 89 ± 21], [PVPBMA, 76 ± 14], for (c,d) PS@$NH_2$ [glass, 93 ± 12], [PVPBMA, 98 ± 19] and PS@$NR_3^+$ [glass, 83 ± 12], [PVPBMA, 87 ± 19]. Please note that the average sample size per frame is displayed in the rectangular brackets. In all images the rectangular area illustrates time period of light illumination with $\lambda$ = 490 nm ($P$ = 11.5 mW·cm$^{-2}$). Particle diameter is (5 ± 0.1) $\mu$m.

**S4 Additional theoretical interpretation of the experimental data.**

In the Theoretical methods section of the main text we have arrived at a model description that depends only on a few parameters: $V_0$ (or, alternatively, $k = V_s^{(0)}/V_0$), $V'_0$ (or, alternatively, $k' = V_s^{(0)}/V'_0$), and $\beta_w$; the phoretic mobility of the particle is expected — based on the observed tendency of moving away from the source of solute — to be negative, and thus $\beta = -1$. Although in principle the phoretic coefficient $b$ could be different for the two types (compact and porous) particles, the reasonable expectation is that it doesn't differ much between the two; for simplicity, here we will assume it to be the same. The size of the illuminated area (the "patch" radius) is fixed to $R = 150\ \mu$m.

For the phoretic mobility of the wall, which in one case is made of glass and in the other case is made of the polymer PVPBMA, we will use $\beta_w = -1$ (which, for the glass wall, is a reasonable expectation). The velocity scales $V_0$ and $V'_0$ depends on the phoretic mobility of the particles and, most importantly, on the rate $Q$ (or $Q'$, for the porous particles) of solute release (but they do not depend on the sizes of either the particle or the patch). From the experimentally observed dependencies of the drift velocity by the shear flow on the intensity and wavelength of the illumination, one infers that the release rates $Q$ and $Q'$, and thus $V_0$ and $V'_0$, do also depend on the intensity and wavelength of the illumination. Accordingly, the interpretation of experiments to extract $V_0$ and $V'_0$ (and the follow up theoretical predictions, based on these parameters, of the drift by the shear flow for particles of various sizes) is done for given parameters (wavelength and intensity) of the light. Afterwards, we will touch upon the experimentally observed dependence of the active hovering on the light intensity parameter $I$, in terms of inverting it into a dependence of $V_0$ and $V'_0$ on $I$.

With this program in aim, one turns to the results in **Figure S13**; they correspond to particles of size $R_p/R_0 = 1.5$, which provide the necessary information to determine $V_0$ and $V'_0$. In all cases, we disregard any active hovering occurring for the compact particles (the experiments clearly suggest that any such tendency is anyway very small, see, e.g., **Figure S13d**).

**S4.1 Illumination at $\lambda$ = 490 nm (Figure S13 (c) and (d))**

Starting with the compact particles (**Figure S11d**), for which the phoretic response to the activity of the wall is the only contribution, the drift velocity $V \approx 38\ \mu$m/s converts, via the **Eq. S4** (see the next section), to $h_{ac}/R_p \approx 1.8$. For this value, **Eq. 21** in the Theoretical methods section of the main text renders $1/k \approx 0.93$; i.e., with $V_s^{(0)}$ from **Eq. 19**, the velocity scale $V_0$ is $V_0 \approx 2.8\ \mu$m/s.

For the porous particles (**Figure S11c**), the hovering above a glass wall involves only the self-motility contribution, **Eq. 23** in the Theoretical methods section of the main text. The drift velocity $V \approx 45$ $\mu$m/s converts, via the **Eq. S4**, to $h_{ac}/R_p \approx 2.1$. For this value, **Eq. 23** renders $1/k' \approx 5.7$, i.e., with $V_s^{(0)}$ from **Eq. 19**, the self-motility velocity scale is $V'_0 \approx 12.5$ $\mu$m/s! This is almost 5 times as large as $V_0$, and — since the phoretic coefficient of the compact and porous silica particles are not expected to be very different — most likely reflect a significantly larger release rate $Q'$ from the porous particle then from the porous polymer coating of the wall. Turning to the second set of data in **Figure S11c**, for the hovering of the porous particles above the polymer coated active wall, the drift velocity $V \approx 80$ $\mu$m/s converts, via the **Eq. S4**, to $h_{ac}/R_p \approx 3.6$. Plugging this, together with $k'$ determined above, into **Eq. 24** renders $1/k \approx 1.2$, and thus for the velocity scale $V_0$ the value $V_0 \approx 2.7$; this is the same as the value estimated from the data in the case of compact particles above an active wall. The result further argues that the phoretic mobilities (the only particle-type dependent parameter entering the definition of $V_0$) of the compact and porous particles are similar, and, consequently, the difference between $V_0$ and $V'_0$ is indeed to be attributed to different fluxes $Q$ and $Q'$ from porous particles and from the polymer coating.

**S4.2 Illumination at λ = 365 nm (Figure S13 a and b)**

The reasoning is the same as the one employed above. From the data in panel (b), one converts the drift velocity of the compact particles above the active wall, $V \approx 40$ $\mu$m/s, to the hovering height $h_{ac}/R_p \approx 1.9$, from which it follows $1/k \approx 0.98$ and thus $V_0 \approx 2.8$ $\mu$m/s. From the data for porous particles above a glass wall in panel (a) one converts the drift velocity $V \approx 75$ $\mu$m/s to a hovering height $h_{ac}/R_p \approx 3.4$. From this, it follows that $1/k' \approx 22$, and thus a quite large $V'_0 \approx 48$ $\mu$m/s. (Note, though, that a Janus particle with half the surface active would move with velocity $\sim V'_0/4 \approx 12$ $\mu$m/s, which is not unusual when compared to experimental reports.)

Finally, the drift velocity $V \approx 150$ $\mu$m/s for porous particles above the active polymer wall converts to $h_{ac}/R_p \approx 6.7$; together with the value above for $V'_0$, it leads (as noted, via **Eq. 24**) to $1/k \approx 1.5$ and thus $V_0 \approx 3.3$ $\mu$m/s, which is again (i.e., as also in the case of the illumination at $\lambda$ = 490 nm), close to the estimate from the compact particles above the active wall.

**S4.3 Predictions for the drift velocity of particles of other sizes**

Having determined the parameters $V_0$ and $V'_0$, the reasoning above can be reversed and used to predict the drift velocity by the shear flow due to the active hovering for particles of different sizes. This proceeds as follows: for a set of $V_0$ and $V'_0$, i.e., a given illumination (wavelength and intensity), one uses one of the **Eqs. 21, 23**, or **24**, according to which of the three situations (type of particles and type of wall) discussed above, to find the hovering height $h_{ac}$ for a particle of radius $R_p$; with this available, **Eq. S4** is then used to calculate the corresponding prediction $U_{sh}$. This procedure is exemplified using the data shown in **Table S3** and it is shown in **Figure 4**) and **Figure S21** for $\lambda$ = 365 nm and for the illumination parameters analyzed in the previous subsections.

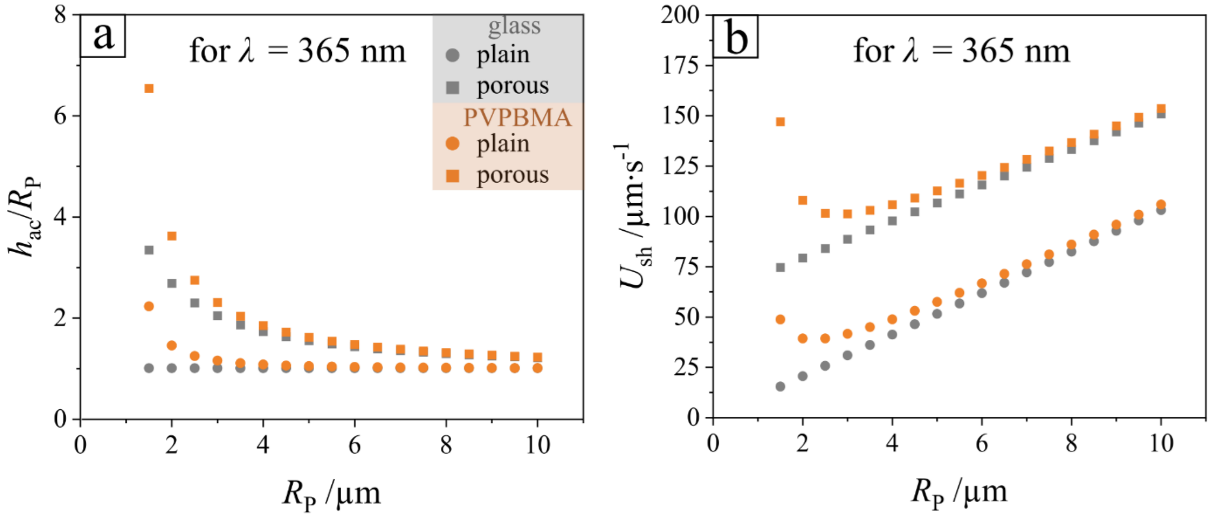


**Figure S21**. Theoretically predicted hovering heights and the corresponding drift velocities by the shear flow, from **Eq. S4** as functions of the particle radius $R_p$. A value of $R = 150R_0$ is used for the radius of the active patch; for the reference case of inert particles and inert wall the value $h/R_p = 1.01$ is used at all $R_p$.

Finally, from the data of drift velocity vs light intensity $I$ at a given wavelength one can extract the dependence of the parameters $V_0$ and $V'_0$ (thus of the surfactant release rate) on the intensity of the illumination. (This could be useful if a model exists to relate the rate of *cis* release to the wavelength and the intensity.) As an example, the data in **Figure S8c**, corresponding to active porous particles of radius $R_p = 1.5$ $\mu$m above an inert glass wall, is interpreted below. The raw data, shown in **Figure S22a**, is transformed into a list of hovering heights by using **Eq. S4** (inset of **Figure S22a**). By using **Eq. 23** and the value $V_s^{(0)}$ corresponding to porous particles, **Eq. 16** in the main text, the $V'_0$ corresponding to each hovering height, i.e., to each intensity, is then obtained. The resulting $V'_0\,(I)$ is shown in **Figure S22b**.

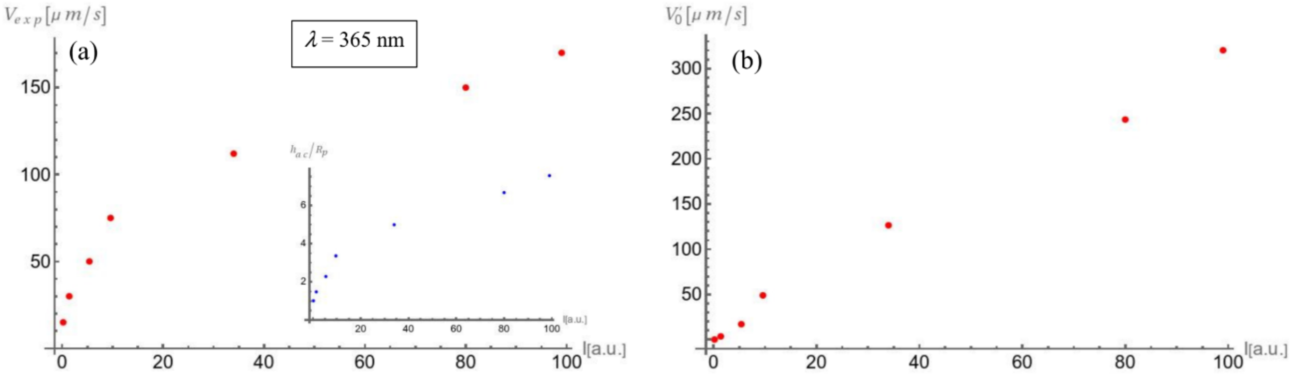


**Figure S22**. (a) Raw data from **Figure S10c**; inset shows the corresponding theoretically predicted hovering heights. (b) Theoretically predicted dependence of the velocity scale $V_0^{\prime}$ of the active porous particles at illumination with $\lambda$ = 365 nm as a function of the light intensity.

### S4.4 Conversion of the measured velocities in shear flow to elevation above the wall

The conversion of the measured velocities into the elevation above the wall is done by approximating near the wall the pressure-driven flow in the microfluidic-channel with a linear shear flow and by employing: (i) for the small particles, the large $z \gg R_p$ asymptotic form (**Eq. 4.8a** in Ref. 4)

$$U_{sh}\left(\bar{z} = z/R_p\right) \simeq \left(S\, R_p\right) \underbrace{\frac{z}{R_p}\left[1 - \frac{5}{16}\left(\frac{R_p}{z}\right)^3\right]}_{:= g(\bar{z})} := \; U_{sh}{}^{(0)} \frac{R_p}{R_0} g(\bar{z}); \qquad \text{S4}$$

for simplicity, we will extrapolate the use of this equation down to $z/R_p \gtrsim 1$, keeping aware that it may lead to some quantitative discrepancies in that range; (ii) for the large particles ($R_p$ = 10 µm/s), the small $z \gg R_p$ asymptotic form (**Eq. 4.8b** in Ref. 4)

$$U_{sh}\left(\bar{z} = z/R_p\right) \simeq \frac{1}{2} U_{sh}{}^{(0)} \frac{R_p}{R_0} \frac{z}{R_p}. \qquad \text{S5}$$

The shear rate $S$ is known to be $S \approx 15\ s^{-1}$ from the calculation of the flow within the channel (based on knowing the cross section of the channel and the volumetric flow rate),[5] and thus the characteristic velocity scale $U_{sh}{}^{(0)} = 15\ \mu$m/s. From **Eqs. S4** and **S5**, the corresponding $z/R_p$ values are obtained simply by reading off from the plot in **Figure S23** the coordinate of the corresponding velocity (as shown there, as an example, for a velocity $U_{sh} = 100\ \mu$m/s).

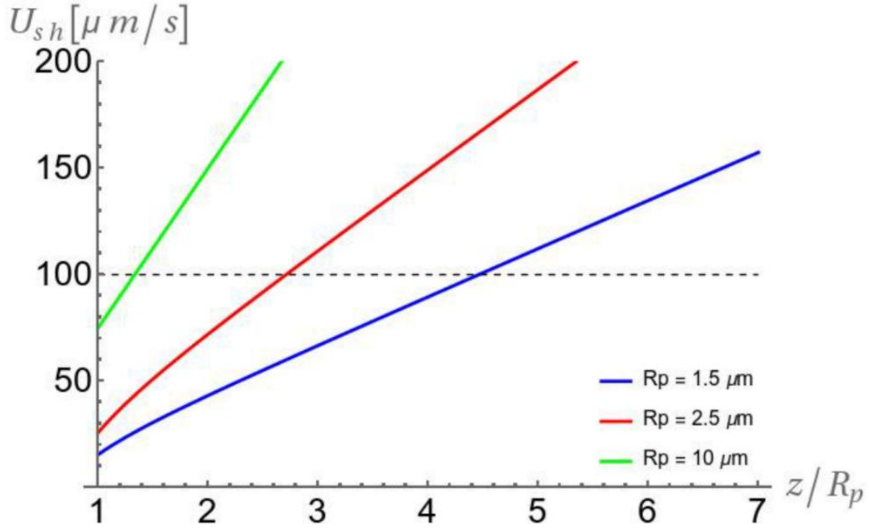


**Figure S23**. The shear flow velocity, **Eqs. S4** and **S5** as a function of $\bar{z} = z/R_p$.

**S5 Passing time calculation**

The principle of separation relies on particles who experience a different shear proportional to their hovering height as a consequence of light induced activity. Then particles will have different velocities, where in first approximation the velocity can be assumed as uniform motion along the streamline. Then the required time until the binary mixture of fast and slow particles separates in the channel can be calculated via:[6]

$$t = \frac{L}{U_{\mathrm{F}} - U_{\mathrm{S}}} = \frac{10000\ \mu\mathrm{m}}{38\ \frac{\mu\mathrm{m}}{\mathrm{s}} - 20\ \frac{\mu\mathrm{m}}{\mathrm{s}}} = 556\ \mathrm{s} = 9.26\ \mathrm{min}\ . \qquad \text{S6}$$

with $U_F$ and $U_S$ as the velocity of fast and slow fraction and $L$ as the length of the initial binary mixture injected in the channel and sedimented on the bottom substrate. We assume this length to be 1 cm. Furthermore, for a particle pair, which can be commonly faced according to preparation procedure ($SiO_2$-OH: [$U_F$ = 38 µm/s], $SiO_2$-$NH_2$: [$U_S$ = 20 $\mu$m/s]) and measured to be the smallest difference (see **Figure 5** and **6 main article**) we calculated a time of 556 s = 9.26 minutes. Note, that in reference [6] is given as a rule of thumb at least a double length for the time of separation/channel length just to have a much better separation performance. Thus, the required time for such particle pair type is 2·9.26 min = 18.52 min ~ 20 min and will be shorter with all other demonstrated examples, as data exhibit much bigger velocity differences.

With set parameters:

Si-OH → $U_F$ = 38 $\mu$m/s

Si-$NH_2$ → $U_S$ = 20 $\mu$m/s

$L$ = 1 cm = 10000 $\mu$m

equation S6 yields for the time 556 s = 18.26 min.

**S6 Description of Supplementary Videos**

**Supplementary Video S1**: Video of inactive-compact particles ($SiO_2$, *D* = [4 ± 0.1] µm) – active wall – PVPBMA at $\lambda$ = 455 nm (*I* = 254 mW $cm^{-2}$) – illuminated and non-illuminated area.

**Supplementary Video S2**: Video of inactive-compact particles ($SiO_2$, *D* = [4 ± 0.1] µm) – inactive wall – glass at $\lambda$ = 455 nm (*I* = 254 mW $cm^{-2}$) – illuminated and non-illuminated area.

**Supplementary Video S3**: Video of active-compact particles ($PSiO_2$, *D* = [3 ± 1] µm) – active wall – PVPBMA at $\lambda$ = 455 nm (*I* = 254 mW $cm^{-2}$) – illuminated and non-illuminated area.

**Supplementary Video S4**: Video of active- porous particles ($PSiO_2$, *D* = [3 ± 1] µm) – inactive wall - glass at $\lambda$ = 455 nm (*I* = 254 mW $cm^{-2}$) – illuminated and non-illuminated area.

**Supplementary Video S5**: Video of non-active compact particles ($SiO_2$, *D* = [4 ± 0.1] µm) – PVPBMA and glass wall at $\lambda$ = 455 nm (*I* = 254 mW $cm^{-2}$). Included Voronoi tessellation.

**Supplementary Video S6**: Video of active porous particles ($PSiO_2$, *D* = [3 ± 1] µm) – PVPBMA and glass wall at $\lambda$ = 455 nm (*I* = 254 mW $cm^{-2}$). Included Voronoi tessellation.

**Supplementary Video S7**: inactive particles ($SiO_2$, *D* = [4 ± 0.1] µm) – glass-PVPBMA-wall at $\lambda$ = 365 nm (*I* = 10 mW·$cm^2$) – static (no flow on).

**Supplementary Video S8**: active particles ($PSiO_2$, *D* = [3 ± 1] µm) – glass-PVPBMA-wall at $\lambda$ = 365 nm (*I* = 10 mW·$cm^2$) – static (no flow on).

**Supplementary Video S9**: active particles ($PSiO_2$, *D* = [5 ± 1] µm) – glass-PVPBMA-wall at $\lambda$ = 365 nm (*I* = 10 mW·$cm^2$) – static (no flow on).

**Supplementary Video S10**: inactive silica particles [$SiO_2$, *D* = (5 ± 0.2) µm] – coating –OH and –$NH_2$ -on glass and PVBMA interface at global illumination with light of $\lambda$ = 490 nm (*P* = 11.5 mW).

**Supplementary Video S11**: inactive polystyrene particles [PS, *D* = (5 ± 0.1) µm] – coating –plain and –$C_{18}$ on glass and PVBMA interface at global illumination with light of $\lambda$ = 490 nm (*P* = 11.5 mW).

**Supplementary Video S12**: Example measurement for comparison glass and PVPBMA – porous silica particle ($PSiO_2$, [3 ± 1] µm) at 455 nm (*I* = 254 mW $cm^{-2}$).

**Supplementary Video S13**: Example measurement for comparison glass and PVPBMA – porous silica particle ($PSiO_2$, [5 ± 1] µm) at 455 nm (*I* = 254 mW $cm^{-2}$).

**Supplementary Video S14**: Example measurement for comparison glass and PVPBMA – porous silica particle ($PSiO_2$, [20 ± 5] µm) at 455 nm (*I* = 254 mW $cm^{-2}$).

**Supplementary Video S15**: Scanning of PVPBMA interface from center position towards outer layer to display porosity distribution.

**References (Supporting Information)**

[1] M. Umlandt, D. Feldmann, E. Schneck, S. A. Santer, M. Bekir, *Langmuir*. **2020**, 36, 46, 14009.

[2] M. Bekir, S. Loebner, A. Kopyshev, N. Lomadze, S. Santer. *Processes*. **2023**, 11, 3, 773.

[3] Origin(Pro), Version *Number* (Version **2022**). OriginLab Corporation, Northampton, MA, USA.

[4] A. Goldman, R. Cox, H. Brenner, *Chem. Eng. Sci*. **1967**, 22, 653.

[5] M. Bekir, M. Sperling, D. Vasquez-Muñoz, C. Braksch, A. Böker, N. Lomadze, M. N. Popescu, S. Santer, *Adv Mater*. **2023**, 35, 2300358.

[6] A. Sharma, F. Rohne, D. Vasquez-Muñoz, S.H. Jung, N. Lomadze, A. Pich, S. Santer, M. Bekir, *Small Methods* **2024**, 8, 2400226.
